# Rotational Motion in Bispidines: A Conformational Study

**DOI:** 10.1021/acs.orglett.5c02839

**Published:** 2025-08-26

**Authors:** Francesco Migliano, Luca Pozzi, Andrea Citarella, Giovanni Macetti, Leonardo Lo Presti, Daniele Passarella, Valerio Fasano

**Affiliations:** † Department of Chemistry, 9304 Università degli Studi di Milano, Via Golgi 19, 20133 Milano, Italy

## Abstract

A detailed conformational analysis of *N*-substituted bispidines has been performed to determine the factors governing the restricted rotational motion induced by the substituents. This investigation combines computational studies of the transition state involved in the rotation with experimental characterization of the rotamers arising from the restricted rotation.

## Graphical abstract

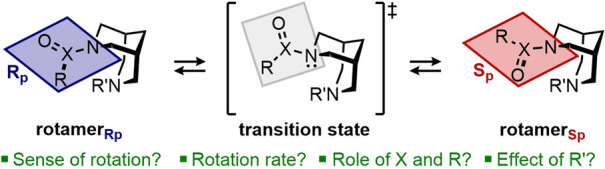

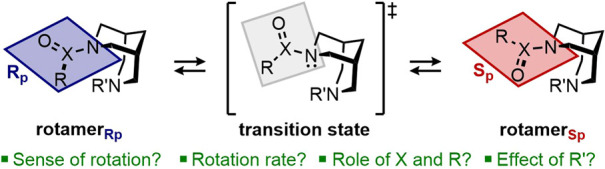

Bispidines (namely 3,7-diazabicyclo[3.3.1]­nonanes) are a class of bicyclic diamines composed of two fused piperidine rings. Found predominantly in natural products such as the quinolizidine alkaloids, bispidines exhibit a range of pharmacological effects, making them of significant interest in drug design.^1–3^ For example, cytisine has been linked to properties such as analgesic, antihypertensive, and antidepressant effects, while sparteine has shown antiarrhythmic and antimicrobial activities (Scheme 1A).^4–6^ Beyond their medicinal uses, bispidine structures have emerged as promising scaffolds in coordination chemistry, molecular motors and organocatalysis.^7–11^ This is the case of bispidine diamides where the relative position of the two carbonyl groups is reminiscent of molecular machine with stops at the syn/anti position, thus allowing chiral–achiral switching (Scheme 1B).^12–16^ While various studies have investigated the relative positioning of the two amide groups, a detailed conformational analysis of rotameric bispidines beyond diamides has not yet been fully explored. This research could have important implications in molecular design since restricted amide bond rotation is key not only in motor motion, but also in foldamers.^17–19^ Herein, a series of rotameric bispidines has been examined experimentally and computationally, with a primary focus on elucidating the chemical behavior of carbonyl-based bispidines. This study aims to uncover the origin of the unexpected desymmetrization observed in NMR spectra (despite the inherent plane of symmetry in the bispidine core) and to understand how substitution on one nitrogen atom influences the rotameric behavior of the opposing amide group. Indeed, uncovering hidden molecular rearrangements has been essential for resolving other puzzling cases of symmetry breaking in NMR spectroscopy.^20^ The insights gained from this work not only enable more confident interpretation of bispidine analyses but also provide valuable guidance for the design of new molecular motors or organocatalysts, where control over rotational movement is crucial to system function.

**Scheme 1:**
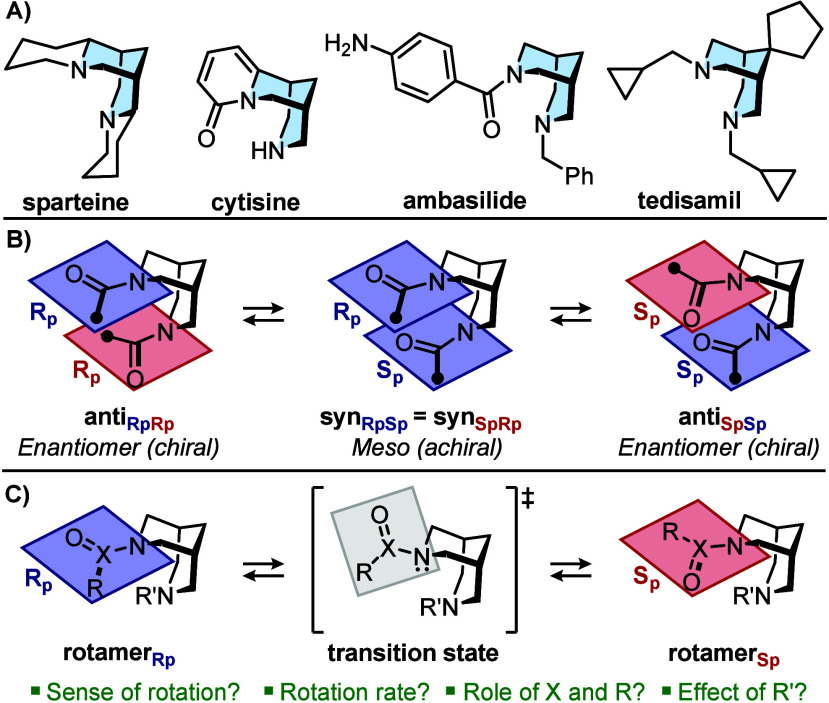
A) Examples of Natural and Synthetic Relevant Bispidines; B) Planar Chirality in Diacyl Bispidine; C) Conformational Study of Rotameric Bispidines (This Work)

Bispidinic diamides exhibit planar chirality, that is the two amides act as rotators linked by a bispidine stator. The latter can assume three conformations (chair–chair, chair–boat, and boat–boat) yet only the former has been mainly associated with these molecular motors.^21,22^ The chair–chair conformation is indeed responsible for the chelating capability of bispidines toward cations, a crucial aspect for their use as ligands in asymmetric synthesis or as ion-channel inhibitors. Taking *N*-acetyl bispidine **Ac-H** as a model substrate, our investigation started modeling the inversion of planar chirality by means of DFT calculations (Scheme 2).

**Scheme 2:**
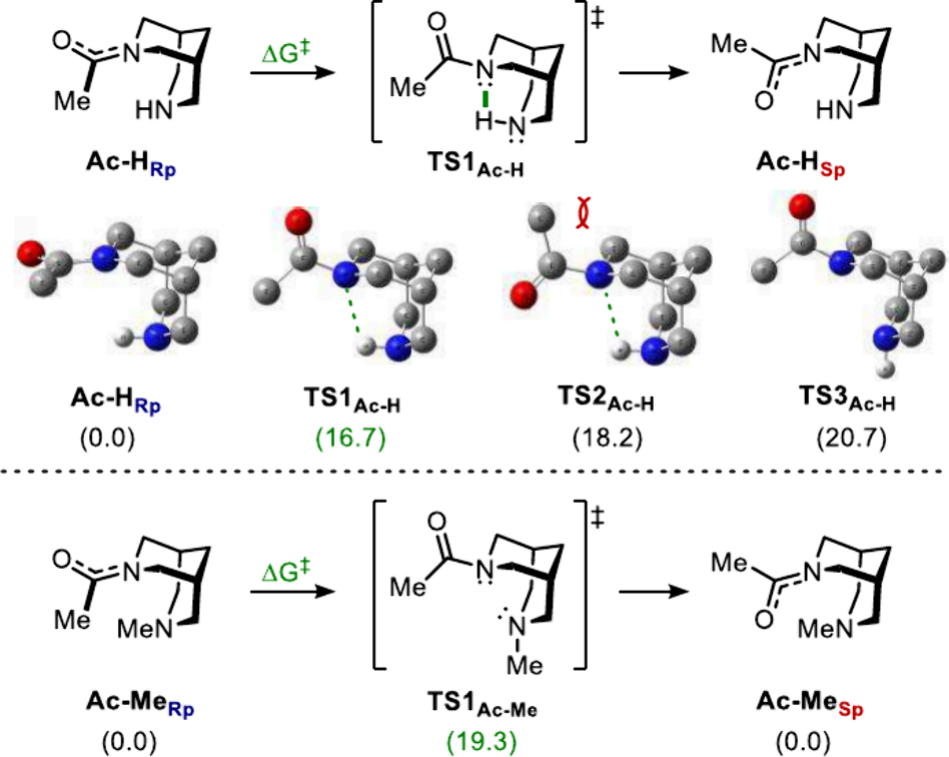
Computed Conformational Analysis for the Inversion of Planar Chirality in **Ac-H** and **Ac-Me** (H Other than *N*′-H Omitted for Clarity)^a^

a In parentheses, relative energy values in kcal/mol.

The modeling was performed at M062X/6-311g+(d,p) including the Polarizable Continuum Model (solvent = chloroform).^21,23^ As expected, the amide bond in **Ac-H**_**Rp**_ was found to be planar, with a significant sp^2^-character for the nitrogen atom (sum of internal angles = 358°) and with its lone pair delocalized throughout the amide bond. The hydrogen on *N′* was located in the axial position: the corresponding **Ac-H**_**Rp**_ with the equatorial *N’*-H had a very similar energy (ΔΔG = 1.5 kcal/mol) and a fully sp^2^-nitrogen (sum of internal angles = 360°). The barrier for the inversion of planar chirality arises from loss of conjugation, with the π_C=O_ system and the nitrogen’s lone pair now orthogonal. This, in turn, causes nitrogen pyramidalization (i.e., partial sp^3^-character), hence the acyl group on the nitrogen can occupy the axial or the equatorial position of the piperidine ring. Given that the hydrogen on the other nitrogen atom can also sit in axial or equatorial position, four transition states are in theory possible (namely *N*_ax_–*N′*_ax_, *N*_ax_–*N′*_eq_, *N*_eq_–*N′*_ax_, *N*_eq_–*N′*_eq_), whereas the relative position of the oxygen atom and the methyl group around the carbonyl carbon further degenerates the system to a total of 8 combinations (see ESI for full analysis). Among them, **TS1**_**Ac‑H**_, having Ac in equatorial and H in axial, was found to be the lowest energy transition state, with an intramolecular H-bonding between the amidic nitrogen and the hydrogen on *N’* being key to stabilization (*N*--H*N*′ = 2.3 Å, with *N*-H-*N′* bond angle = 114.6). This interaction, resembling the chelation of bispidines to metals, was also found on the second most stable transition state (**TS2**_**Ac‑H**_). Yet, in the latter, unfavorable steric interactions with two axial hydrogens of the piperidine chair destabilize the transition state (bond angle N–C–CH_3_ = 113.9 vs 118.1 in **TS1**_**Ac‑H**_ and **TS2**_**Ac‑H**_, respectively). Given that the difference in energy between **TS1**_**Ac‑H**_ and **TS2**_**Ac‑H**_ is expected to increase replacing Ac with bigger acyl groups, it can be stated that the inversion of planar chirality from R_p_ to S_p_ should follow a clockwise rotation (looking along the C_Ac_–N bond in R_p_) since this would avoid steric clashes between the acyl carbon and the two axial hydrogens (for very large systems like dipivaloyl bispidine, the presence of two *tert*-butyl groups causes a different mechanism of rotation).^24^ The third most stable transition state (**TS3**_**Ac‑H**_) was found 4.0 kcal/mol higher in energy than **TS1**_**Ac‑H**_, most likely due to the absence of the stabilizing interaction. This was supported by an increase in the Wiberg bond order of the *N′*-H bond (from 0.82 in **TS1**_**Ac‑H**_ to 0.84 in **TS3**_**Ac‑H**_), indicating a weaker *N′*-H bond in the former. Replacing R’ with a methyl group, the steric congestion inside the bicyclic cavity increases significantly to a point where the most accessible transition state (**TS1**_**Ac‑Me**_) had Ac and Me both in equatorial positions. Nevertheless, the clockwise rotation is still retained, given the destabilizing interactions of the methyl group of Ac with the axial hydrogens of the piperidine. However, the absence of the intramolecular H-bonding observed in **TS1**_**Ac‑H**_ causes a relatively higher energy barrier (ΔG^‡^ = 16.7 kcal/mol vs 19.3 kcal/mol **TS1**_**Ac‑H**_ and **TS1**_**Ac‑Me**_, respectively), thus resulting in a slower rotation of the amide plane. Having established the key features of the inversion of planar chirality in *N*-acyl bispidines, we turned our attention to Boc-protected bispidines (**Boc-R’**), versatile intermediates in quinolizidine alkaloids synthesis with an underexplored potential in molecular motors (Scheme 3).

**Scheme 3:**
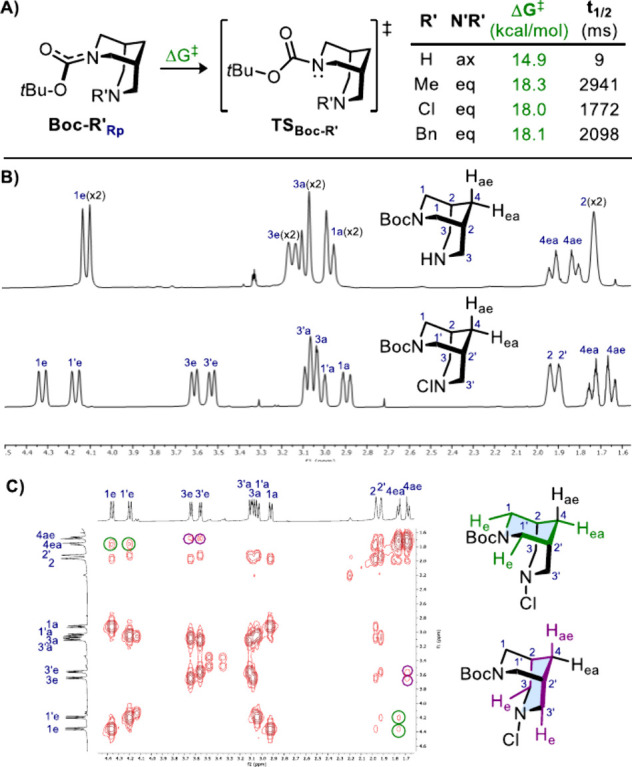
A) Computed Conformational Analysis for the Inversion of Planar Chirality in **Boc-R′**; B) Zoom of ^1^H NMR Spectra (CDCl_3_, 298 K) of **Boc-H** and **Boc-Cl**; C) COSY Spectrum (CDCl_3_, 298 K) of **Boc-Cl** (the Green and Purple Circles Show the ^4^J_H–H_ Long-Range Couplings)

A first computational approach on **Boc-H** revealed similarities with *N*-acyl bispidines, with an intramolecular H-bonding favoring the inversion of planar chirality (**TS1**_**Boc‑H**_ = 14.9 kcal/mol). This value is smaller than that of **TS1**_**Ac–H**_ (16.7 kcal/mol), implying a faster amide bond rotation. This was attributed to weaker destabilizing interactions with axial hydrogens H_1a_ since carbamate rotation could occur basically in both senses (**TS2**_**Boc‑H**_ = 15.0 kcal/mol). Indeed, the oxygen atoms of Boc have similar volumes given that the t-butyl group is placed far away from the bispidine core. Replacing R’ with other substituents causes the preference for the equatorial position which, in turn, causes a significant rise in the energy barrier (Scheme 3A). Indeed, **Boc-Me**, **Boc-Cl**, and **Boc-Bn** were all predicted to slowly interconvert between the two planar chiral conformations, with a half-life of approximately two seconds (for **Boc-H** instead *t*_1/2_ = 9 ms).^25^ Given that processes requiring two seconds are likely to be in slow exchange at 400 MHz,^26^ the prediction was confirmed by ^1^H NMR analysis since an average structure was observed for **Boc-H** and well-resolved spectra for **Boc-R′** (Scheme 3B for R′ = Cl and Supporting Information for R′ = Me or Bn). The chemical shift separations between 1e and 1e′ for R′ = Cl, Me, and Bn were 0.16, 0.13, and 0.15 ppm, corresponding to frequency separations of 52, 64, and 60 Hz, respectively, on a 400 MHz NMR spectrometer (or NMR time scales of 4.3, 3.5, and 3.8 ms).^27^ These frequency differences are significantly larger than those associated with dynamic processes that have lifetimes around 2 s. Therefore, these compounds exhibit a mutual and equally populated slow exchange on the NMR time scale.^28^ It has to be noted that despite the increase in the energy barrier for the inversion of the planar chirality in **Boc-R′**, these values would not ensure the physical separation of the two enantiomers. Moreover, the rotation is also solvent-dependent, as confirmed by the absence of observable rotamers for **Boc-Bn** when running the NMR analysis in MeOH-*d*_4_.^29^ To ensure that the degeneration of the signals is not due to the equilibrium between the chair–chair and chair-boat conformations (as observed in related systems),^21^ a 2D NMR analysis was performed on **Boc-Cl**. The COSY NMR spectrum revealed two distinct sets of ^4^J_H–H_ long-range couplings between equatorial protons (highlighted in green and purple in Scheme 3C), confirming the chair conformations of both rings in solution. These couplings, referred to as W-couplings, are especially pronounced in carbocycles that constrain the coupled protons in a W-conformation.^24,30^ Interestingly, **Boc-Cl** is an important example because it could enable the introduction of sp^2^-substituents (e.g., aryl rings or pyridines) via chloride displacement with Grignard reagents. This process would then give rise to a new class of molecular motors based on the relative positions of the planes containing the Boc group and the aromatic ring. Having observed the interconversion between enantiomeric forms of **Boc-R′**, our attention focused then on the installation of a stereogenic center on R′ since it would create diastereomeric rotamers. This led to the synthesis of **Boc-PEA** whose ^1^H NMR analysis is reported in Scheme 4. At room temperature, two sets of equatorial protons adjacent to the amide were observed in almost equal amounts (nonmutual, unequally populated slow exchange), as expected for nearly isoenergetic diastereomers. Similarly, two methyl and two *tert*-butyl groups were observed in ^1^H NMR and ^13^C NMR, confirming diastereomeric structures. Variable Temperature NMR on **Boc-PEA** revealed coalescence around 70 °C, a temperature corresponding to a roughly estimated free energy of activation of 17.1 kcal/mol.^31^ It should be noted that this value is approximate since only one experimental data point was measured. Above 90 °C, fully resolved spectra were observed, with the disappearance of the planar chirality leading to a single set of two equatorial protons (diastereotopic due to the presence of central chirality). The last type of rotameric bispidines investigated belongs to the family of *N*-nitrosamines since it is known that restricted rotation of the N-NO bond gives rise to rotamers.^32,33^ Yet, despite the similarities with a −CHO group, some differences have been noted between the rotamers of *N*-nitroso and *N*-formyl piperidones.^34^ Therefore, **NO-Bn** was synthesized and analyzed by ^1^H NMR (Scheme 5A). Also in this case, slow interconversion of planar chirality was observed (mutual, equally populated slow exchange, Δν = 44 Hz), with the benzylic protons being now diastereotopic due to the presence of planar chirality (a similar spectrum was also observed replacing CDCl_3_ with MeOH-*d*_4_).

**Scheme 4:**
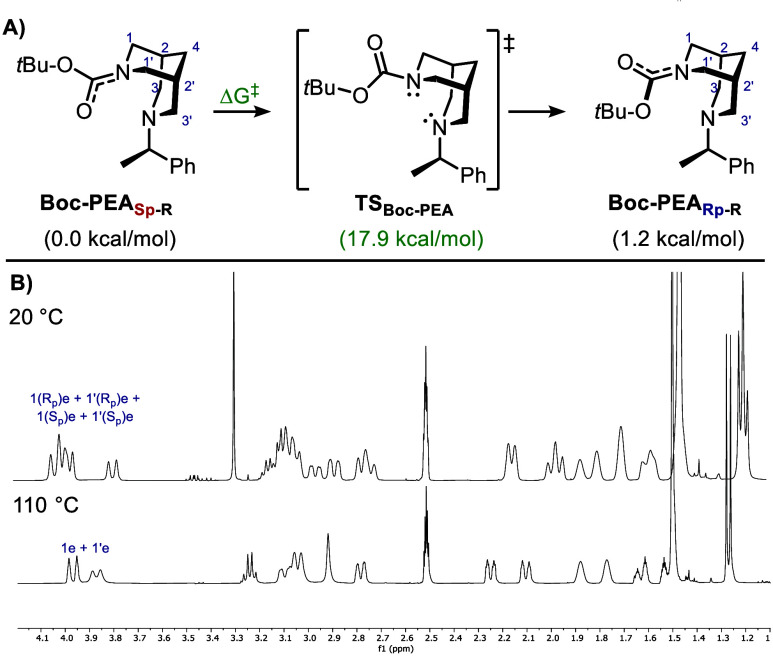
A) Inversion of Planar Chirality in **Boc-PEA**; B) Zoom of ^1^H NMR Spectra in DMSO-*d*_6_ below and above the Coalescence Temperature

**Scheme 5:**
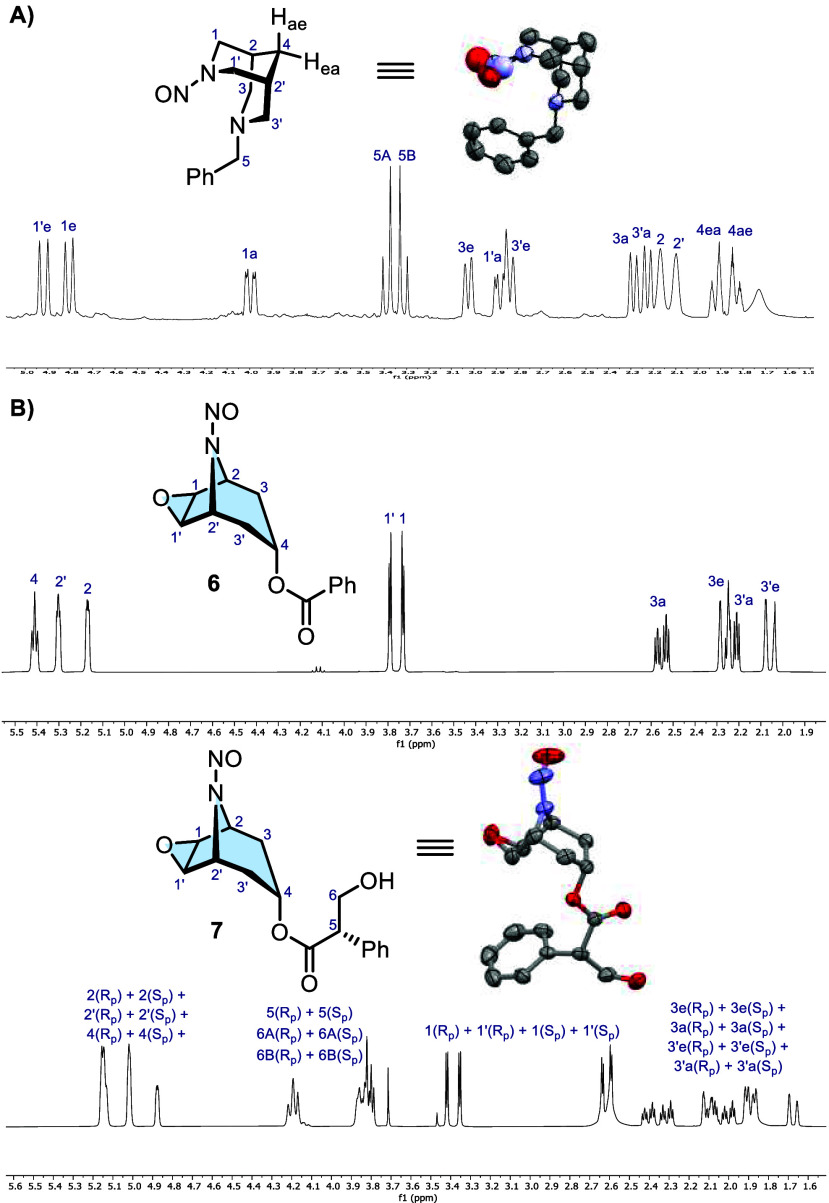
Zoom of the ^1^H NMR Spectra (CDCl_3_, 298 K) of *N*-Nitrosamines **NO-Bn** (A) and Tropane Derivatives **6** and **7** (B)^a^

a For clarity, hydrogens are omitted in the molecular structures.

Interestingly, in the solid state, the chair–chair conformation of **NO-Bn** exhibited a 74:26 disorder at the nitroso group. This occurs because crystal packing restricts the rotation of the phenyl ring, thereby locking the two rotamers into rigid conformations. Unlike in solution, where the Bn group rotates freely, this restriction causes the two rotamers to become diastereomers, thus energetically inequivalent. It should be emphasized that the observation of rotamers in *N*-nitrosamines is not restricted to bispidines. Indeed, given the urgent problem of nitrosamine impurities in active pharmaceutical ingredients,^35^ the formation of rotamers should be considered when characterizing this class of impurities. To confirm this, we synthesized the *N*-nitrosamine of another bicyclic structure belonging to the family of tropane alkaloids (Scheme 5B).^36^ By means of NMR analysis, enantiomeric rotamers for scopine derivative **6** (mutual, equally populated slow exchange) and diastereomeric rotamers for scopolamine derivative **7** (nonmutual, unequally populated slow exchange) were still observed, thus confirming restricted N–NO bond rotation also in these nitrosamines. Interestingly, the diastereomeric rotamers of **7** crystallized separately as confirmed by XRD analysis, showing, in the solid state, no dislocation of the electron density of the nitrosaminic oxygen over two sites.

In conclusion, the inversion of planar chirality in N–R bispidines (R = Ac, Boc, NO) was investigated (Scheme 6). This process involves significant pyramidalization of the amidic nitrogen and may influence symmetry breaking of the bispidine core in NMR analysis. In unsubstituted carbonyl bispidines, an attractive interaction between the amidic nitrogen and N′–H stabilizes the transition state and enables rapid carbonyl rotation, resulting in a fast exchange regime. When a substituent is present on N′, the energy barrier increases by 2–3 kcal/mol, leading to restricted carbonyl rotation (slow exchange regime). This unexpected effect is also observed in *N*-nitrosobispidines and other related natural systems.

**Scheme 6:**
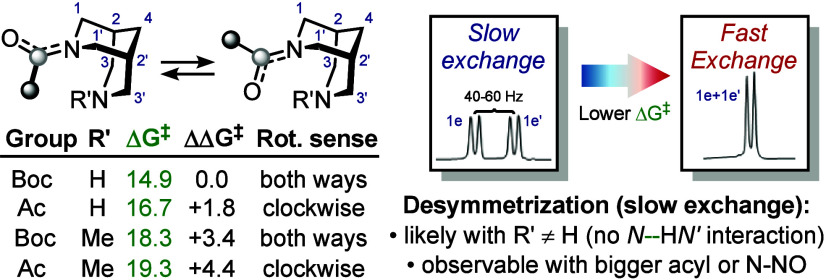
Summary of Carbonyl Bispidines Analyses

## Supplementary Material



## Data Availability

The data underlying this study are available in the published article and its Supporting Information.

## Notes

NMR characterizations, DFT calculations, XRD analysis (PDF)

## References

[1] Singh H., Khatoon N., Bhardwaj S. K., Kampani P., Nayak T. K., Haridas V. (2023). Bispidine as a Versatile Scaffold: From Topological
Hosts to Transmembrane Transporters. ChemBioChem..

[2] Tomassoli I., Gündisch D. (2016). Bispidine
as a Privileged Scaffold. Curr. Top. Med. Chem..

[3] Comba P., Kerscher M., Rück K., Starke M. (2018). Bispidines for radiopharmaceuticals. Dalt. Trans..

[4] Wang X., Yang J., Huang P., Wang D., Zhang Z., Zhou Z., Liang L., Yao R., Yang L. (2024). Cytisine:
State of the art in pharmacological activities and pharmacokinetics. Biomed. Pharmacother..

[5] Rouden J., Lasne M. C., Blanchet J., Baudoux J. (2014). (−)-Cytisine
and Derivatives: Synthesis, Reactivity, and Applications. Chem. Rev..

[6] Villalpando-Vargas F., Medina-Ceja L. (2016). Sparteine as an anticonvulsant drug: Evidence and possible
mechanism of action. Seizure.

[7] Breuning M., Steiner M. (2008). Chiral Bispidines. Synthesis
(Stuttg)..

[8] Yang Z., Liu J., Liu X., Wang Z., Feng X., Su Z., Hu C. (2008). Highly Efficient
Amine Organocatalysts Based on Bispidine forthe
Asymmetric Michael Addition of Ketones to Nitroolefins. Adv. Synth. Catal..

[9] Bulygina L. A., Kagramanov N. D., Khrushcheva N. S., Lyssenko K. A., Peregudov A. S., Sokolov V. I. (2017). Unsymmetrical pincer CNN palladium complex of 7-ferrocenylmethyl-3-methyl-3,7-diazabicyclo[3.3.1]­nonane. J. Organomet. Chem..

[10] Phuan P. W., Ianni J. C., Kozlowski M. C. (2004). Is the
A-Ring of Sparteine Essential
for High Enantioselectivity in the Asymmetric Lithiation–Substitution
of N-Boc-pyrrolidine?. J. Am. Chem. Soc..

[11] Li G., Wang R., Ye D., Pu M., Feng X., Lin L. (2024). Bispidine-Based S,N-Chiral Ligands
for Palladium-Catalyzed Asymmetric
Arylation of Cyclic N-Sulfonyl Ketimines. Eur.
J. Org. Chem..

[12] Krut’ko D. P., Medved’ko A. V., Lyssenko K. A., Churakov A. V., Dalinger A. I., Kalinin M. A., Gudovannyy A. O., Ponomarev K. Y., Suslov E. V., Vatsadze S. Z. (2022). Bispidine Platform as a Tool for
Studying Amide Configuration Stability. Molecules.

[13] Palyulin V. A., Emets S. V., Chertkov V. A., Kasper C., Schneider H.-J. (1999). Conformational
Switching of 3,7-Diacyl-3,7-diazabicyclo[3.3.1]­nonanes by Metal Binding
and by Solvent Changes. Eur. J. Org. Chem..

[14] Singh H., Chenna A., Gangwar U., Borah J., Goel G., Haridas V. (2021). Bispidine as a β-strand
nucleator: from a β-arch
to self-assembled cages and vesicles. Chem.
Sci..

[15] Wang Z., Islam M. J., Vukotic V. N., Revington M. J. (2016). Conformational
Study of N,N′-Diacyl Bispidines and Dioxo Bis-bispidines: Planar
Chirality and Molecular Switching. J. Org. Chem..

[16] Pisarev S. A., Golubeva E. A., Palyulin V. A. (2024). Ab Initio
Conformational Analysis
of 3,7-Diacetyl-3,7-Diazabicyclo[3.3.1]­Nonanes. Nat. Prod. Commun..

[17] Le
Bailly B. A. F., Clayden J. (2016). Dynamic foldamer chemistry. Chem. Commun..

[18] Tomasini C., Angelici G., Castellucci N. (2011). Foldamers Based on Oxazolidin-2-ones. Eur. J. Org. Chem..

[19] Goodman C. M., Choi S., Shandler S., DeGrado W. F. (2007). Foldamers as versatile
frameworks for the design and evolution of function. Nat. Chem. Biol..

[20] McGlinchey M. J. (2014). Symmetry
Breaking in NMR Spectroscopy: The Elucidation of Hidden Molecular
Rearrangement Processes. Symmetry.

[21] Castellano C., Sacchetti A., Meneghetti F. (2016). Spectroscopic, Structural, and Computational
Characterization of Three Bispidinone Derivatives, as Ligands for
Enantioselective Metal Catalyzed Reactions. Chirality.

[22] Zefirov N.
S., Palyulin V. A. (1991). Topics in Stereochemistry.

[23] Gorske B. C., Stringer J. R., Bastian B. L., Fowler S. A., Blackwell H. E. (2009). New Strategies
for the Design of Folded Peptoids Revealed by a Survey of Noncovalent
Interactions in Model Systems. J. Am. Chem.
Soc..

[24] Singh H., Chenna A., Gangwar U., Dutta S., Kurur N. D., Goel G., Haridas V. (2023). Bispidine
as a promising scaffold
for designing molecular machines. Org. Biomol.
Chem..

[25] www.metadynamics.cz/eyring/eyring.html.

[26] Nikitin K., O’Gara R. (2019). Mechanisms
and Beyond: Elucidation of Fluxional Dynamics
by Exchange NMR Spectroscopy. Chem.—Eur.
J..

[27] Bryant R. G. (1983). The NMR
time scale. J. Chem. Educ..

[28] Bain A. D. (2003). Chemical
exchange in NMR. Prog. Nucl. Magn. Reson. Spectrosc..

[29] Bean J. W., Nelson D. J. (1984). The effect of solvent polarity upon
rotational barriers in nikethamide. Biochem.
Pharmacol..

[30] Gillet R., Roux A., Brandel J., Huclier-Markai S., Camerel F., Jeannin O., Nonat A. M., Charbonnière L. J. (2017). A Bispidol
Chelator with a Phosphonate Pendant Arm: Synthesis, Cu­(II) Complexation,
and ^64^Cu Labeling. Inorg. Chem..

[31] Jameson B., Glaser R. (2024). VT-NMR Analysis of
Rotation-Inversion of N-(4-hydroxybutyl)-N-(2,2,2-trifluoroethyl)
tert-butyl Carbamate: Utilizing the – CH2CF3 Appendage as a
Reporter on E/Z-Isomerization. ChemistrySelect.

[32] Cechová L., Procházková E., Císarová I., Dracínský M., Janeba Z. (2014). Separation of planar
rotamers through intramolecular hydrogen bonding in polysubstituted
5-nitrosopyrimidines. Chem. Commun..

[33] Gdaniec M., Milewska M. J., Połoński T. (1995). Conformational
Study
of *N*-Nitroso-2,6-diphenylpiperidines and *N*-Nitroso-2,6-diphenylpiperidin-4-ones by Molecular Mechanics
Calculations, X-ray Crystallography, and ^1^H and ^13^C NMR Spectroscopy. J. Org. Chem..

[34] Ponnuswamy S., Sethuvasan S., Thirunavukarasu K. (2015). Synthesis, characterisation, stereochemistry
and dynamic NMR studies of *N*-nitroso and *N*-formyl-t-3-isopropyl-r-2,c-6-bis­(4-methoxyphenyl)­piperidin-4-ones. J. Mol. Struct..

[35] Vikram H. P. R., Kumar T. P., Kumar G., Beeraka N. M., Deka R., Suhail S. M., Jat S., Bannimath N., Padmanabhan G., Chandan R. S., Kumar P., Gurupadayya B. (2024). Nitrosamines
crisis in pharmaceuticals – Insights on toxicological implications,
root causes and risk assessment: A systematic review. J. Pharm. Anal..

[36] Huang J. P., Wang Y. J., Tian T., Wang L., Yan Y., Huang S. X. (2021). Tropane alkaloid biosynthesis: a centennial review. Nat. Prod. Rep..

