# Supplementary material for: Rotational Motion in Bispidines: A Conformational Study
Source: Org Lett. 2025 Aug 26;27(35):9669–73. doi: 10.1021/acs.orglett.5c02839 (PMC12418502; doi:10.1021/acs.orglett.5c02839)
Supplement: Supplementary file 1 [file ol5c02839_si_001.pdf]

Supporting Information for

# Rotational Motion in Bispidines: A Conformational Study

Francesco Migliano,[a] Luca Pozzi,[a] Andrea Citarella,[a] Giovanni Macetti,[a] Leonardo Lo Presti,[a] Daniele Passarella,*[a] Valerio Fasano*[a]

Correspondence to:

daniele.passarella@unimi.it; valerio.fasano@unimi.it

[a] Department of Chemistry, Università degli Studi di Milano, Via Camillo Golgi, 19, 20133 Milano

# TABLE OF CONTENTS

1. MATERIALS AND GENERAL METHODS ........ 2
1.1. Chemistry ........ 3
2. EXPERIMENTAL DATA ........ 5
2.1 Overview on the synthesis of bispidines ........ 5
2.2 Synthesis of 2 ........ 5
2.3 Synthesis of Boc-Bn ........ 6
2.4 Synthesis of Boc-H ........ 7
2.5 Synthesis of Boc-Cl ........ 8
2.6 Synthesis of 3 ........ 8
2.7 Synthesis of Boc-PEA ........ 9
2.8 Synthesis of Boc-Me ........ 10
2.9 Synthesis of 4 ........ 11
2.10 Synthesis of Bn-NO ........ 11
2.11 Overview on the synthesis of tropanes ........ 13
2.12 Synthesis of 5 ........ 13
2.13 Synthesis of 6 ........ 14
2.14 Synthesis of 7 ........ 15
3. SPECTROSCOPIC DATA ........ 16
$^{1}$H NMR (400 MHz, $CDCl_3$) of 2 (see procedure) ........ 16
$^{1}$H NMR (400 MHz, $CDCl_3$) of Boc-Bn (see procedure) ........ 17
$^{1}$H NMR (400 MHz, $CD_3OD$) of Boc-Bn (see procedure) ........ 18
$^{13}$C NMR (400 MHz, $CDCl_3$) of Boc-Bn (see procedure) ........ 19
COSY of Boc-Bn (see procedure) ........ 20
HSQC of Boc-Bn (see procedure) ........ 21
HMBC of Boc-Bn (see procedure) ........ 22
$^{1}$H NMR (400 MHz, $CD_3OD$) of Boc-H (see procedure) ........ 23
$^{1}$H NMR (400 MHz, $CDCl_3$) of Boc-Cl (see procedure) ........ 24
$^{13}$C NMR (400 MHz, $CDCl_3$) of Boc-Cl (see procedure) ........ 25
COSY of Boc-Cl (see procedure) ........ 26
HSQC of Boc-Cl (see procedure) ........ 27
HMBC of Boc-Cl (see procedure) ........ 28
$^{1}$H NMR (400 MHz, CDCl3) of Boc-PEA (see procedure) ........ 29
$^{13}$C NMR (400 MHz, $CDCl_3$) of Boc-PEA (see procedure) ........ 30

COSY of Boc-PEA (see procedure) 31
HSQC of Boc-PEA (see procedure) 32
HMBC of Boc-PEA (see procedure) 33
VT NMR (400 MHz, DMSO) of Boc-PEA (see procedure) 34
$^{1}$H NMR (400 MHz,$CDCl_3$) of Boc-Me (see procedure) 35
$^{13}$C NMR (400 MHz,$CDCl_3$) of Boc-Me (see procedure) 36
COSY of Boc-Me (see procedure) 37
HSQC of Boc-Me (see procedure) 38
$^{1}$H NMR (400 MHz,$CDCl_3$) of 4 (see procedure) 39
$^{1}$H NMR (400 MHz,$CDCl_3$) of Bn-NO (see procedure) 40
$^{1}$H NMR (400 MHz, $CD_3OD$ ) of Bn-NO (see procedure) 41
$^{13}$C NMR (400 MHz,$CDCl_3$) of Bn-NO (see procedure) 42
COSY of Bn-NO (see procedure) 43
HSQC of Bn-NO (see procedure) 44
HMBC of Bn-NO (see procedure) 45
$^{1}$H NMR (400 MHz,$CDCl_3$) of 5 (see procedure) 46
$^{13}$C NMR (400 MHz,$CDCl_3$) of 5 (see procedure) 47
COSY of 5 (see procedure) 48
HSQC of 5 (see procedure) 49
HMBC of 5 (see procedure) 50
$^{1}$H NMR (400 MHz,$CDCl_3$) of 6 (see procedure) 51
$^{13}$C NMR (400 MHz,$CDCl_3$) of 6 (see procedure) 52
COSY of 6 (see procedure) 53
HSQC of 6 (see procedure) 54
HMBC of 6 (see procedure) 55
$^{1}$H NMR (400 MHz,$CDCl_3$) of 7 (see procedure) 56
$^{13}$C NMR (400 MHz,$CDCl_3$) of 7 (see procedure) 57
COSY of 7 (see procedure) 58
HSQC of 7 (see procedure) 59
HMBC of 7 (see procedure) 60
4. DFT CALCULATIONS 61
5. CRYSTALLOGRAPHIC DATA 89
6. REFERENCES 96

# 1. MATERIALS AND GENERAL METHODS

## 1.1. Chemistry

Unless stated, all starting materials and anhydrous solvents were obtained from commercial sources and used without purification. Reactions were carried out under an inert atmosphere of nitrogen unless stated. Reaction progress was monitored by TLC, with $^{1}H$ NMR or LC-MS analyses taken from reaction samples. Column chromatography was performed on silica gel (230-400 mesh) or automated Isolera One Flash Chromatography (Biotage). $^{1}H$ NMR and $^{13}C$ NMR spectra were recorded at 298 K on a Brüker Avance Spectrometer (400 and 600 MHz), using commercially available deuterated solvents ($CDCl_3$, MeOD). VT NMR spectra of Boc-PEA were recorded at 304 K, 323 K, 343 K, 363 K, 383 K on a Brüker Avance Spectrometer (400 MHz), using commercially available deuterated solvent (DMSO). $^{1}H$ NMR chemical shifts are reported in ppm relative to protio impurities in the deuterated solvents and reported as follow: chemical shift (multiplicity, coupling constants, number of protons). $^{13}C$ NMR chemical shifts are reported in ppm using the solvent resonance. Coupling constants *J* are given in Hertz (Hz), while the multiplicity of the signals are indicated as “s”, “d”, “t”, “q”, “pent”, “sept” or “m” for singlet, doublet, triplet, quartet, pentet, septet or multiplet, respectively. Structural assignments were made with additional information from gCOSY, gHSQC, and gHMBC experiments. Mass spectra were recorded on a Thermo Fisher LCQ Fleet Ion Trap Mass Spectrometer. Compound names are those generated by ChemDraw Professional 20.0 software (PerkinElmer), following the IUPAC nomenclature. The following safety-related information is provided to address potential hazards associated with the handling, use, and disposal of selected chemical reagents employed in this study:

**Methyl Iodide**

Methyl Iodide can cause cancer according to an independent committee of scientific and health experts. Methyl iodide appears as a colorless liquid that turns brown on exposure to light. Denser than water. Contact may irritate skin, eyes and mucous membranes. Very toxic by ingestion, inhalation and skin absorption.

**Acetic acid**

Acetic acid, solution, appears as a colorless aqueous solution. Smells like vinegar. Corrosive to metals and tissue.

**Sodium borohydride**

Sodium borohydride is a white to grayish crystalline powder. It is decomposed by water to form sodium hydroxide, a corrosive material, and hydrogen, a flammable gas. The heat of this reaction may be sufficient to ignite the hydrogen. The material itself is easily ignited and burns

vigorously once ignited.

**4 M HCl in dioxane**

At room temperature, hydrochloric acid is a colorless to slightly yellow, corrosive, nonflammable gas that is heavier than air and has a strong irritating odor. On exposure to air, hydrogen chloride forms dense white corrosive vapors. Hydrocloric acid causes severe skin burns and eye damage. Toxic if inhaled.

***p*-Toluenesulfonyl hydrazide**

*p*-Toluenesulfonyl hydrazide is a white nearly odorless powder used as a reagent and scavenger. May explode if heated under confinement; Temperatures above 120 °C or prolonged temperatures above 50 °C can cause exothermic decomposition with evolution of gas. It is a mild eye irritant and toxic if ingested.

# 2. EXPERIMENTAL DATA

## 2.1 Overview on the synthesis of bispidines

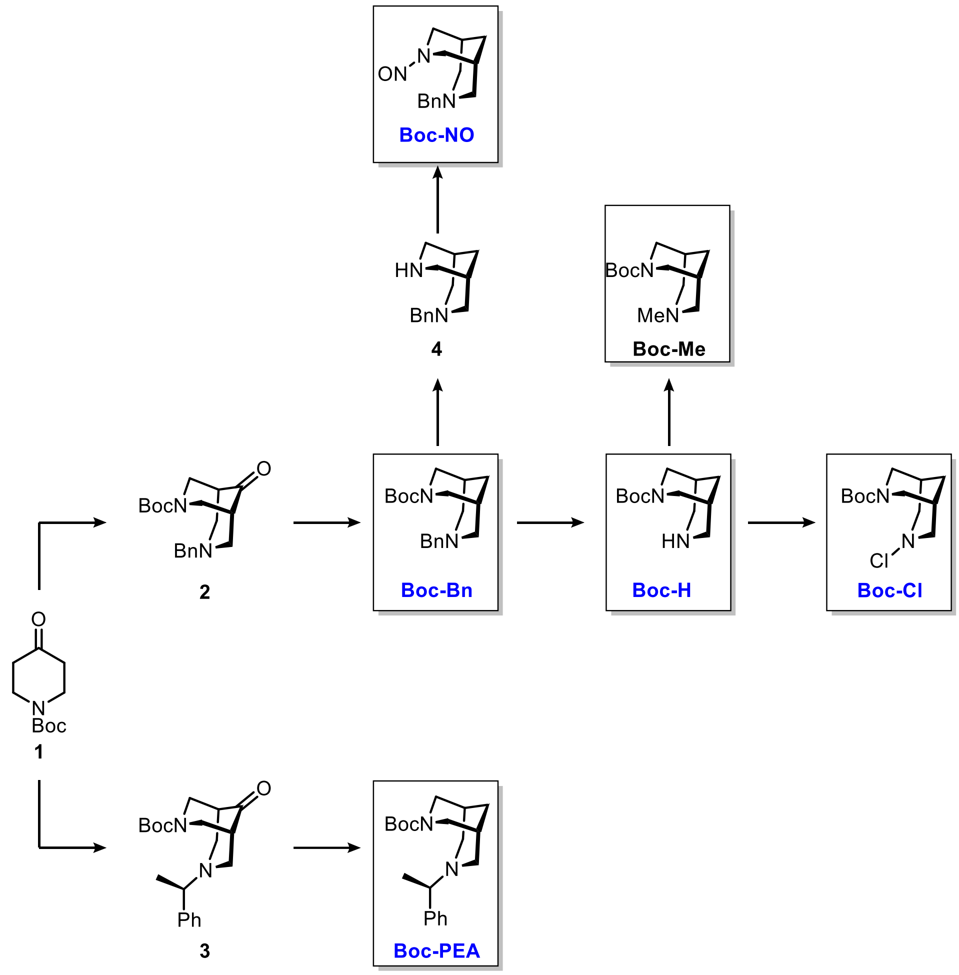


**Figure S1.** General synthetic scheme for bispidine scaffolds.

## 2.2 Synthesis of 2

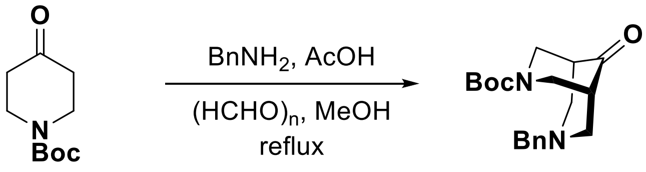


1 2

To a suspension of paraformaldehyde (0.33 g, 11.0 mmol, 2.2 equiv) in MeOH (20 mL) heated at reflux in an oil bath, a solution of 1-Boc-4-piperidone (1.00 g, 5.02 mmol, 1.0 equiv), AcOH (0.30 g, 5.02 mmol, 1.0 equiv) and benzylamine (0.55 g, 5.12 mmol, 1.02 equiv) in MeOH (25 mL) was added dropwise over a period of 1 h. After the addition, paraformaldehyde (0.33 g, 11.0 mmol, 2.2 equiv)

was added and the mixture was stirred for 5 h. After completion of the reaction, the solvents were removed under reduced pressure, the crude was dissolved in diethyl ether (20 mL) and washed with a solution of NaOH 1 M (10 mL). The aqueous layer was extracted with diethyl ether (5 × 10 mL). The organic phase was dried with anhydrous $Na_2SO_4$, filtered, and concentrated under reduced pressure. The crude was purified with column chromatography (*n*-hex/AcOEt, 75:25 *v/v*) to afford intermediate **2** as a colorless liquid (1.094 g, 3.31 mmol, 66% yield).

**$^1$H NMR (400 MHz, $CDCl_3$):** rotamers present δ 7.32 – 7.22 (m, 5H, Ph H), 4.56 (d, $^3J_{H,H}$= 13.5 Hz, 1H, C*H*$_2$-NBoc), 4.40 (d, $^3J_{H,H}$= 13.5 Hz, 1H, C*H*$_2$-NBoc), 3.50 (d, $^3J_{H,H}$= 13.5 Hz, 2H, benzylic C*H*$_2$), 3.35 (d, $^3J_{H,H}$= 13.5 Hz, 1H, -NC*H*$_2$), 3.27 (d, $^3J_{H,H}$= 13.5 Hz, 1H, -NC*H*$_2$), 3.18 (d, $^3J_{H,H}$= 13.5 Hz, 1H, -NC*H*$_2$), 3.15 (d, $^3J_{H,H}$= 13.5 Hz, 1H, -NC*H*$_2$), 2.72 (d, $^3J_{H,H}$= 13.5 Hz, 1H, -NC*H*$_2$), 2.66 (d, $^3J_{H,H}$= 13.5 Hz, 1H, -NC*H*$_2$), 2.41 (br, 2H, bridgehead C*H*), 1.53 (s, 9H, Boc C*H*$_3$).

Spectroscopic data are in accordance to those reported in literature[1].

See spectra

## 2.3 Synthesis of Boc-Bn

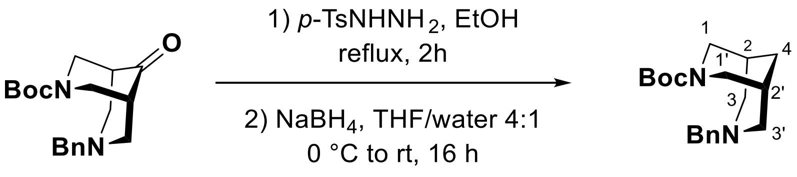


**2** **Boc-Bn**

To a solution of compound **2** (1.094 g, 3.31 mmol, 1.0 equiv) in EtOH (30 mL) heated at reflux in an oil bath, *p*-toluenesulfonyl hydrazide (0.74 g, 3.97 mmol, 1.2 equiv) was added in one portion and the solution was left stirring until disappearance of compound **2** (about 2 h). After completion of the reaction, the solvent was removed under reduced pressure. The crude was dissolved in a mixture of THF/water 4:1 (17 mL). To this solution stirred at 0 °C, $NaBH_4$ (1.25 g, 33.1 mmol, 10 equiv) was added portionwise over a period of 20 minutes and the mixture was left stirring at room temperature for 16 h. Then the mixture was stirred at reflux in an oil bath for 3 h. After being allowed to cool to rt, water (30 mL) was added, and the aqueous layer was extracted with diethyl ether (7 × 10 mL). The organic phase was dried with anhydrous $Na_2SO_4$, filtered, and concentrated under reduced pressure. The crude was purified with column chromatography (*n*-hex/AcOEt, 8:2 *v/v*) to afford compound **Boc-Bn** as a colourless oil (0.524 g, 1.66 mmol, 50% yield).

**$^1$H NMR (400 MHz, $CDCl_3$):** rotamers present δ 7.34 – 7.19 (m, 5H, Ph H), 4.15 (d, $^3J_{H,H}$= 13.3 Hz, 1H, equatorial C*H*$_2$ 1), 4.00 (d, $^3J_{H,H}$= 13.3 Hz, 1H, equatorial C*H*$_2$ 1'), 3.45 (d, $^2J_{H,H}$= 13.5 Hz, 1H, PhC*H*$_2$), 3.30 (d, $^2J_{H,H}$= 13.5 Hz, 1H, PhC*H*$_2$), 3.11 (m, 1H, axial C*H*$_2$ 1), 3.07 (m, 1H, axial C*H*$_2$ 1'),

2.97 (d, $^3J_{H,H}$= 11.0 Hz, 1H, equatorial C$H_2$ 3), 2.89 (d, $^3J_{H,H}$= 11.0 Hz, 1H, equatorial C$H_2$ 3'), 2.22 (d, $^3J_{H,H}$= 11.0 Hz, 1H, axial C$H_2$ 3), 2.17 (d, $^3J_{H,H}$= 11.0 Hz, 1H, axial C$H_2$ 3'), 1.87 (br, s, 1H, C*H* 2), 1.79 (br, s, 1H, C*H* 2'), 1.65 (m, 2H, C$H_2$ 4), 1.52 (s, 9H, Boc C$H_3$).

**$^{13}$C NMR (100 MHz, CDCl$_3$):** δ 155.2 (C=O), 139.2 (Ph C), 128.7 (Ph *C*), 128.2 (Ph *C*), 126.8 (Ph *C*), 78.8 (*C*CH3), 63.7 (benzylic $CH_2$), 59.2 (*C*$H_2$ 2), 58.9 (*C*$H_2$ 2'), 48.6 (*C*$H_2$ 1), 47.7 (*C*$H_2$ 1'), 31.3 (*C*$H_2$ 4), 29.2 (*C*H 2-2'), 28.9 (Boc *C*$H_3$).

**$^1$H NMR (400 MHz, MeOD)** δ 7.31 – 7.17 (m, 5H, Ph *H*), 4.06 (d, $^1J_{H,H}$= 13.0 Hz, 2H, equatorial C$H_2$ 1), 3.35 (s, 2H, benzylic C$H_2$) 3.12 (d, $^3J_{H,H}$= 11.0 Hz, 1H, equatorial C$H_2$ 3), 3.03 (d, $^3J_{H,H}$= 11.0 Hz, 1H, equatorial C$H_2$ 3'), 2.93 (d, $^1J_{H,H}$= 13.0 Hz, 2H, equatorial C$H_2$ 1), 2.24 – 2.19 (m, 2H, axial C$H_2$ 3-3'), 1.85 (br, s, 2H, 1H, C*H* 2), 1.81 (br, s, 2H, 1H, C*H* 2') 1.73 (br, 1H, C$H_2$ $4_{ea}$), 1.65 (br, 1H, C$H_2$ $4_{ae}$), 1.52 (s, 9H, Boc C$H_3$).

Spectroscopic data are in accordance to those reported in literature[1].

See spectra

## 2.4 Synthesis of Boc-H

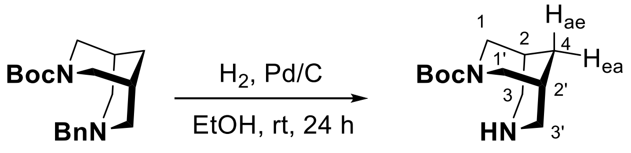


**Boc-Bn** **Boc-H**

Pd/C 10 % *w/w* (0.62 g, 35 mmol% loading) was added to a solution of **Boc-Bn** (0.524 g, 1.66 mmol, 1.0 equiv) in EtOH (11 mL). The atmosphere was exchanged for hydrogen three times. The mixture was stirred for 24 h, and the reaction was monitored by TLC. Once the starting material was consumed, the mixture was filtered over Celite and the solvent was removed under reduced pressure affording compound **Boc-H** as a brown oil (0.33 g, 1.46 mmol, 88% yield).

**$^1$H NMR (400 MHz, MeOD)** δ 4.09 (d, $^1J_{H,H}$= 13.0 Hz, 2H, equatorial C$H_2$ 1) , 3.14 (br, 2H, equatorial C$H_2$ 3), 3.07 (br, 2H, axial C$H_2$ 3), 2.96 (d, $^1J_{H,H}$= 13.0 Hz, 2H, axial C$H_2$ 1), 1.95 (br, 1H, C$H_2$ $4_{ea}$), 1.81 (br, 1H, C$H_2$ $4_{ae}$), 1.74 (br, 2H, C*H* 2), 1.49 (s, 9H, Boc C$H_3$), N*H* missing.

**MS (ESI)** *m/z:* $[M+H]^+$ Calcd for $C_{12}H_{22}N_2O_2H$ 227.18; Found 227.00

Spectroscopic data are in accordance to those reported in literature[2].

See spectra

## 2.5 Synthesis of Boc-Cl

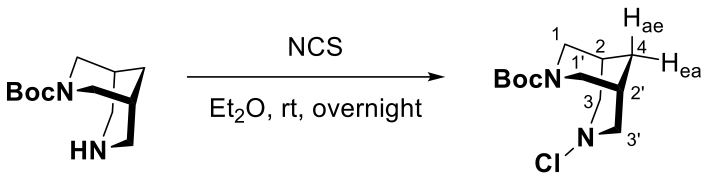


**Boc-H** **Boc-Cl**

To a solution of **Boc-H** (0.134 g, 0.59 mmol, 1.0 equiv) in dry $Et_2O$ (3 mL), *N*-chlorosuccinimide (0.087 g, 0.65 mmol, 1.1 equiv) was added in one portion and the mixture was left stirring at room temperature overnight. After completion of the reaction, EtOH (0.4 mL) was added to the mixture, the solvent was removed under reduced pressure and the crude was filtered over a silica plug using a mixture *n*-hex/AcOEt 4:1 as eluent, affording compound **Boc-Cl** as a white solid (0.087 g, 0.33 mmol, 56% yield).

**$^1$H NMR (400 MHz, $CDCl_3$):** δ 4.34 (d, $^3J_{H,H}$= 13.5 Hz, 1H, equatorial C*H*$_2$ 1), 4.18 (d, $^3J_{H,H}$= 13.5 Hz, 1H, equatorial C*H*$_2$ 1’), 3.62 (d, $^3J_{H,H}$= 10.0 Hz, 1H, equatorial C*H*$_2$ 3), 3.54 (d, $^3J_{H,H}$= 10.0 Hz, 1H, equatorial C*H*$_2$ 3’), 3.08 (m, 1H, axial C*H*$_2$ 3), 3.05 (m, 1H, axial C*H*$_2$ 3’), 3.03 (m, 1H, axial C*H*$_2$ 1), 2.90 (m, 1H, axial C*H*$_2$ 1’), 1.95 (br, 1H, C*H* 2), 1.90 (br, 1H, C*H* 2’), 1.75 (br, 1H, C*H*$_2$ 4$_{ea}$), 1.64 (br, 1H, C*H*$_2$ 4$_{ae}$), 1.48 (s, 9H, Boc C*H*$_3$).

**$^{13}$C NMR (100 MHz, $CDCl_3$):** δ 155.3 (C=O), 79.4 (*C*CH$_3$), 68.1 (*C*H$_2$ 3), 67.8 (*C*H$_2$ 3’), 48.5 (*C*H$_2$ 1), 47.3 (*C*H$_2$ 1’), 33.3 (2C, *C*H$_2$ 2-2’), 31.4 (*C*H$_2$ 4), 28.7 (*C*H$_3$).

**MS (ESI)** *m/z:* [M+Na]$^+$ Calcd for $C_{12}H_{21}ClN_2O_2Na$ 283.12; Found 283.00

See spectra

## 2.6 Synthesis of 3

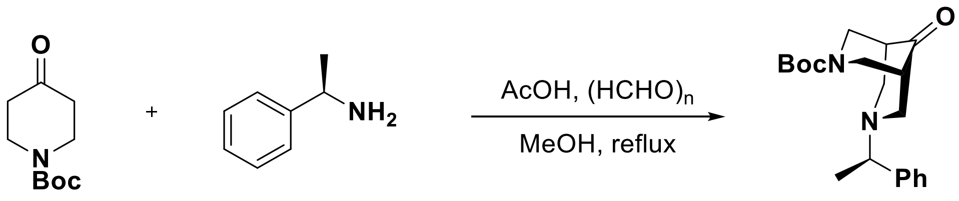


**1** **(*R*)-1-phenylethan-1-amine** **3**

To a suspension of paraformaldehyde (0.33 g, 11.0 mmol, 2.2 equiv) in MeOH (20 mL) heated at reflux in an oil bath, a solution of 1-Boc-4-piperidone (1.00 g, 5.02 mmol, 1.0 equiv), AcOH (0.30 g, 5.02 mmol, 1.0 equiv) and (*R*)-1-phenylethan-1-amine (0.62 g, 5.12 mmol, 1.02 equiv) in MeOH (25

mL) was added dropwise over a period of 1 h. After the addition, paraformaldehyde (0.33 g, 11.0 mmol, 2.2 equiv) was added and the mixture was stirred for 5 h. After completion of the reaction the solvents were removed under reduced pressure, the crude was dissolved in diethyl ether (20 mL) and washed with a solution of NaOH 1 M (10 mL). The aqueous layer was extracted with diethyl ether (5 × 10 mL). The organic phase was dried with anhydrous $Na_2SO_4$, filtered, and concentrated under reduced pressure. The crude was filtered through a silica plug to afford intermediate **3** (1.24 g, 3.6 mmol, 72% yield) which was immediately used for the next reaction without any further purification.

See spectra

## 2.7 Synthesis of Boc-PEA

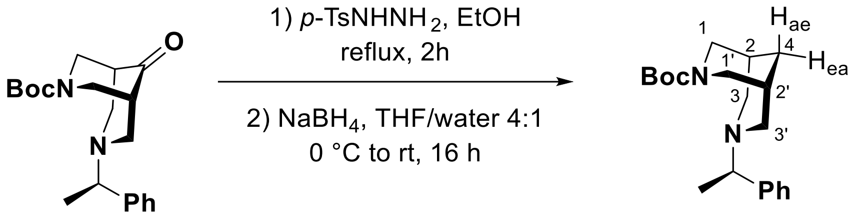


**3** **Boc-PEA**

To a solution of compound **3** (0.62 g, 1.80 mmol, 1.0 equiv) in EtOH (15 mL) heated at reflux in an oil bath, *p*-toluenesulfonyl hydrazide (0.40 g, 2.16 mmol, 1.2 equiv) was added in one portion and the solution was left stirring until the disappearance of compound **3** (about 2 h). After completion of the reaction, the solvents were removed under reduced pressure. The crude was dissolved in a mixture THF/water 4:1 (9 mL). To this solution stirred at 0 °C, $NaBH_4$ (0.68 g, 18 mmol, 10 equiv) was added portion-wise over a period of 20 minutes and the mixture was left stirring at room temperature for 16 h. Then the mixture was stirred at reflux in an oil bath for 3 h. After being allowed to cool to rt, water (30 mL) was added, and the aqueous layer was extracted with diethyl ether (7 × 10 mL). The organic phase was dried with anhydrous $Na_2SO_4$, filtered, and concentrated under reduced pressure. The crude was purified with column chromatography (*n*-hex/AcOEt 8:2 *v/v*) to afford compound **Boc-PEA** as a yellow oil (0.287 g, 0.87 mmol, 48% yield).

**$^1$H NMR (400 MHz, $CDCl_3$):** δ 7.32 – 7.28 (m, 8H, Ph H, rotamers), 7.21 (br, 2H, Ph H, rotamers), 4.13 (br, 1H, equatorial C$H_2$ 1, rotamer), 4.10 (br, 1H, equatorial C$H_2$ 1, rotamer), 4.08 (br, 1H, equatorial C$H_2$ 1', rotamer), 3.86 (d, $^1J_{H,H}$ = 12.9 Hz, 1H, equatorial C$H_2$ 1', rotamer), 3.13 (br, 1H, equatorial C$H_2$ 3, rotamer), 3.12 (br, 2H, benzylic C*H*, rotamers), 3.10 (br, 1H, axial C$H_2$ 1, rotamer), 3.08 (br, 1H, axial C$H_2$ 1, rotamer), 3.06 (br, 1H, axial C$H_2$ 1', rotamer), 3.00 (br, 1H, axial C$H_2$ 1', rotamer), 2.87 (d, $^1J_{H,H}$= 10.7 Hz, 1H, equatorial C$H_2$ 3, rotamer), 2.78 (d, $^1J_{H,H}$ = 10.9 Hz, 1H, equatorial C$H_2$ 3', rotamer), 2.22 (d, $^1J_{H,H}$= 10.6 Hz, 2H, axial C$H_2$ 3, equatorial C$H_2$ 3', rotamers), 2.04 (br, 2H, axial C$H_2$ 3-3', rotamers), 1.92 (br, 1H, C*H* 2, rotamer), 1.82 (br, 1H, C*H* 2', rotamer),

1.73 (br, 2H, C*H* 2-2’, rotamer), 1.72 (br, 1H, axial C*H*$_2$ 3’, rotamer), 1.64 (br, 2H, C*H*$_2$ 4, rotamers), 1.56 (br, 2H, C*H*$_2$ 4, rotamers), 1.54 (s, 9H, Boc C*H*$_3$, rotamer), 1.53 (s, 9H, Boc C*H*$_3$, rotamer), 1.29 (br, 3H, C*H*$_3$ rotamer), 1.26 (s, 3H, C*H*$_3$ rotamer).

**$^{13}$C NMR (100 MHz, CDCl$_3$):** δ 128.3 (Ph *C*, rotamer), 128.2 (Ph *C*, rotamer), 127.7 (Ph *C*, rotamer), 127.6 (Ph *C*, rotamer), 126.6 (Ph *C*, rotamer), 65.5 (benzylic *C*H), 57.3 (*C*H$_2$ 2’, rotamer), 56.6 (*C*H$_2$ 2’, rotamer), 55.9 (*C*H$_2$ 2, rotamer), 48.9 (*C*H$_2$ 1, rotamer), 48.3 (*C*H$_2$ 1, rotamer), 48.0 (*C*H$_2$ 1’, rotamer), 47.6 (*C*H$_2$ 1’, rotamer), 31.6 (*C*H$_2$ 4, rotamer), 31.0 (*C*H$_2$ 4, rotamer), 29.5 (*C*H 2, rotamer), 29.2 (*C*H 2’, rotamer), 29.2 (*C*H 2, rotamer), 28.9 (Boc *C*H$_3$, rotamer), 28.9 (Boc *C*H$_3$, rotamer), 20.5 (*C*H$_3$, rotamer), 20.4 (*C*H$_3$, rotamer).

**MS (ESI)** *m/z:* [M+H]$^+$ Calcd for $C_{20}H_{30}N_2O_2H$ 331.24; Found 331.20

See spectra

The VT-NMR of **Boc-PEA** showed the coalescence around 343 K, and, at this temperature, the maximum difference in chemical shift was measured for two equatorial protons adjacent to the amide (Δvmax = 40 Hz). Using these two values in the Shanan-Atidi and Bar-El equation, an energy barrier of 17.1 kcal/mol was estimated. See VT NMR

## 2.8 Synthesis of Boc-Me

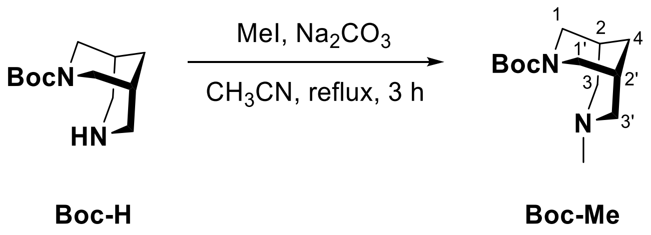


To a solution of **Boc-H** (0.072 g, 0.32 mmol, 1.0 equiv) in dry $CH_3CN$ (3 mL), $Na_2CO_3$ (0.170 g, 1.6 mmol, 5.0 equiv) and MeI (0.136 g, 0.96 mmol, 3.0 equiv) were added and the mixture was left stirring at reflux in an oil bath for 3 h. After completion of the reaction, the solvent was removed under reduced pressure, the crude was dissolved in AcOEt and filtered. The organics were washed with a saturated solution of $NaHCO_3$ (3 × 10 mL), dried with anhydrous $Na_2SO_4$ and filtered. The solvent was removed under reduced pressure affording **Boc-Me** as a brown oil (0.040 g, 0.17 mmol, 53% yield).

**$^1$H NMR (400 MHz, CDCl$_3$):** δ 4.22 (br, 1H, equatorial 1’), 4.09 (br, 1H, equatorial 1), 2.93 (m, 4H), 2.16 (br, 2H), 2.07 (s, 3H, C*H*$_3$), 1.75 (br, 2H), 1.64 (d, $J_{H,H}$ = 13.0, 1H, $4_{ea}$), 1.56 (d, $J$ = 13.0, 1H, $4_{ae}$), 1.43 (s, 9H, Boc C*H*$_3$).

**$^{13}$C NMR (100 MHz, CDCl$_3$):** δ 155.8 (Boc *C*O), 78.8 (Boc *C*-CH$_3$), 60.2 (*C*H$_2$ 3), 59.8 (*C*H$_2$ 3), 49.3 (*C*H$_2$ 1’), 48.2 (*C*H$_2$ 1), 46.8 (*C*H$_3$), 30.5 (*C*H 2-2’), 29.8 (*C*H$_2$ 4), 28.7 (Boc *C*H$_3$).

**MS (ESI)** *m/z:* $[M+H]^+$ Calcd for $C_{13}H_{24}N_2O_2H$ 241.19; Found 241.30

Spectroscopic data are in accordance to those reported in literature[3].

See spectra

## 2.9 Synthesis of 4

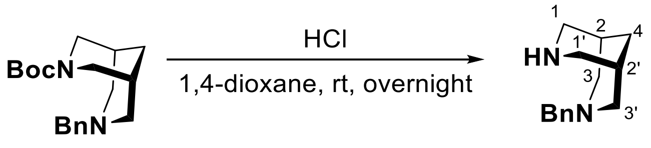


**Boc-Bn** **4**

To a solution of Boc-Bn (0.400 g, 1.26 mmol, 1.0 equiv) in 1,4-dioxane (12 mL), a solution of 4M HCl 4 in 1,4-dioxane was added (12 mL) and the mixture was left stirring at room temperature overnight. After completion of the reaction, the solvent was removed under reduced pressure, the crude was treated with a solution of KOH 1 M (50 mL) and stirred for 1 h. The aqueous phase was extracted with diethyl ether (5 × 10 mL). The organics were dried with anhydrous $Na_2SO_4$ and filtered. The solvent was removed under reduced pressure affording **4** as a colourless oil (0.269 g, 1.24 mmol, quantitative yield).

**$^1$H NMR (400 MHz, $CDCl_3$):** δ 7.30 (m, 4H, Ph *H*), 7.24 (br, 1H, Ph *H*), 3.67 - 3.51 (m, 2H), 3.37 (s, 2H, benzylic C$H_2$), 3.10 - 2.96 (m, 4H), 2.37 (d, 2H), 1.83 (d, 1H, C$H_2$ 4), 1.68 (br, 1H, C$H_2$ 4), 1.68 (s, 2H, C*H* 2), N*H* missing.

Spectroscopic data are in accordance to those reported in literature[4].

See spectra

## 2.10 Synthesis of Bn-NO

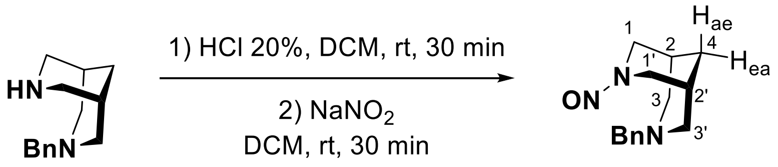


**Bn-H** **Bn-NO**

To a solution of **4** (0.100 g, 0.462 mmol, 1.0 equiv) in DCM (2.5 mL), a 20% *w/w* aqueous solution of HCl was added dropwise over 30 minutes, followed by the portion-wise addition of $NaNO_2$. The reaction mixture was stirred at room temperature for an additional 30 minutes until complete

consumption of the starting material was observed. The reaction was then quenched by the addition of a saturated $Na_2CO_3$ solution. The layers were separated and the aqueous phase was extracted with DCM (3 × 10 mL). The combined organic layers were dried over anhydrous $Na_2SO_4$, filtered, and concentrated under reduced pressure to afford the **Bn-NO** as a white solid (0.087 g, 0.355 mmol, 77% yield).

**$^1$H NMR (400 MHz, $CDCl_3$):** δ 7.25 – 7.10 (m, 5 H, Ph *H*), 4.89 (d, $^1J_{H,H}$ = 14.7 Hz, 1H, equatorial C$H_2$ 1'), 4.78 (d, $^1J_{H,H}$ = 13.4 Hz, 1H, equatorial C$H_2$ 1), 3.97 (d, $^1J_{H,H}$ = 13.4 Hz, 1H, axial C$H_2$ 1), 3.36 (d, $^1J_{H,H}$ = 13.2 Hz, 1H, benzylic C$H_2$), 3.29 (d, $^1J_{H,H}$ = 13.2 Hz, 1H, benzylic C$H_2$), 3.00 (d, $^1J_{H,H}$ = 11.2 Hz, 1H, equatorial C$H_2$ 3), 2.88 (d, $^1J_{H,H}$ = 14.7 Hz, 1H, axial C$H_2$ 1'), 2.83 (br, 1H, equatorial C$H_2$ 3'), 2.26 (d, $^1J_{H,H}$ = 11.2 Hz, 1H, axial C$H_2$ 3), 2.20 (d, $^1J_{H,H}$ = 11.0 Hz, 1H, axial C$H_2$ 3'), 2.14 (br, 1H, C*H* 2), 2.07 (br, 1H, C*H* 2'), 1.89 (d, $^1J_{H,H}$ = 12.8 Hz, 1H, C$H_2$ $4_{ea}$), 1.81 (d, $^1J_{H,H}$ = 12.8 Hz, 1H, C$H_2$ $4_{ae}$).

**$^{13}$C NMR (100 MHz, $CDCl_3$):** δ 134.2 (Ph C), 128.8 (Ph *C*), 128.4 (Ph *C*), 127.1 (Ph *C*), 63.2 (benzylic *C*$H_2$), 59.0 (*C*$H_2$ 3'), 58.1 (*C*$H_2$ 3), 55.2 (*C*$H_2$ 1), 45.1 (*C*$H_2$ 1'), 31.1 (*C*$H_2$ 4), 29.4 (*C*H 2), 28.6 (*C*H 2').

**$^1$H NMR (400 MHz, MeOD)** δ 7.25 – 7.10 (m, 5 H, Ph *H*), 4.90 (d, $^1J_{H,H}$ = 14.7 Hz, 1H, equatorial C$H_2$ 1'), 4.70 (d, $^1J_{H,H}$ = 13.4 Hz, 1H, equatorial C$H_2$ 1), 4.00 (d, $^1J_{H,H}$ = 13.4 Hz, 1H, axial C$H_2$ 1), 3.00 (d, $^1J_{H,H}$ = 11.2 Hz, 1H, equatorial C$H_2$ 3), 2.90 (d, $^1J_{H,H}$ = 14.7 Hz, 1H, axial C$H_2$ 1'), 2.78 (d, $^1J_{H,H}$ = 11.3 Hz, 1H, equatorial C$H_2$ 3'), 2.29 (d, $^1J_{H,H}$ = 11.2 Hz, 1H, axial C$H_2$ 3), 2.21 (d, $^1J_{H,H}$ = 11.0 Hz, 1H, axial C$H_2$ 3'), 2.15 (br, 1H, C*H* 2), 2.08 (br, 1H, C*H* 2'), 2.01 (d, $^1J_{H,H}$ = 12.8 Hz, 1H, C$H_2$ $4_{ea}$), 1.82 (d, $^1J_{H,H}$ = 12.8 Hz, 1H, C$H_2$ $4_{ae}$), benzylic $CH_2$ falls under signal of methanol.

**MS (ESI)** *m/z:* $[M+H]^+$ Calcd for $C_{14}H_{19}N_3OH$ 246.16; Found 246.20

See spectra

## 2.11 Overview on the synthesis of tropanes

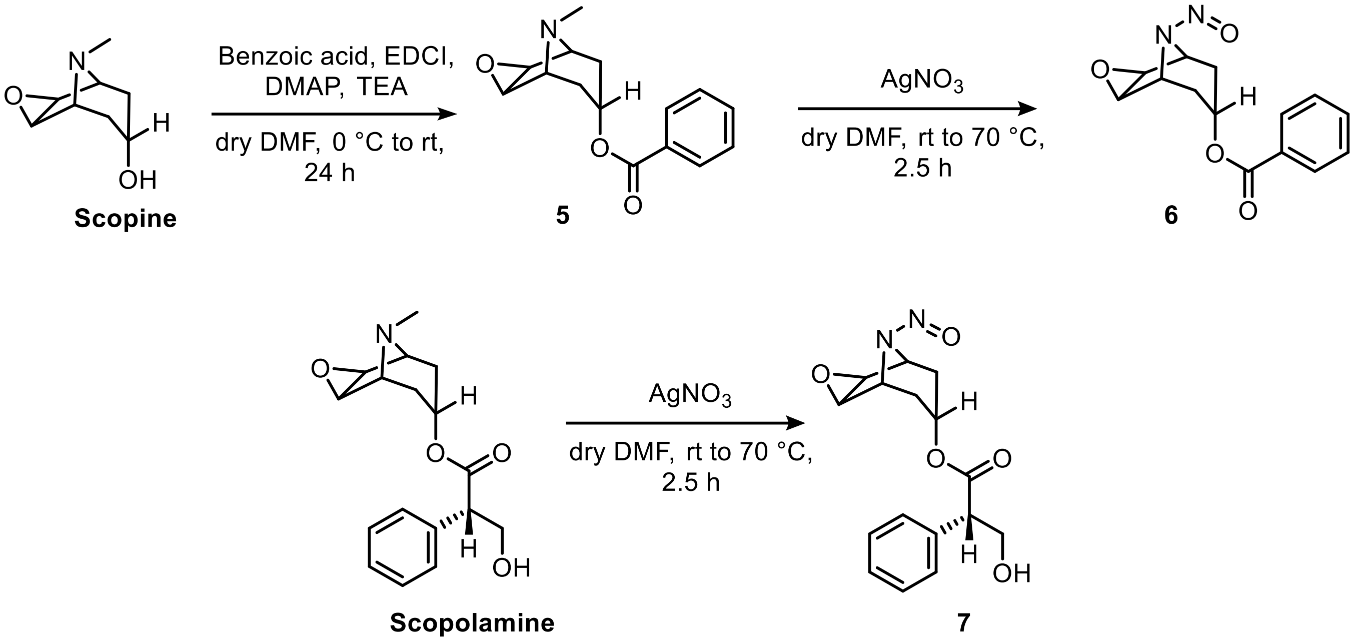


**Figure S2**. General synthetic scheme for tropane scaffolds.

## 2.12 Synthesis of 5

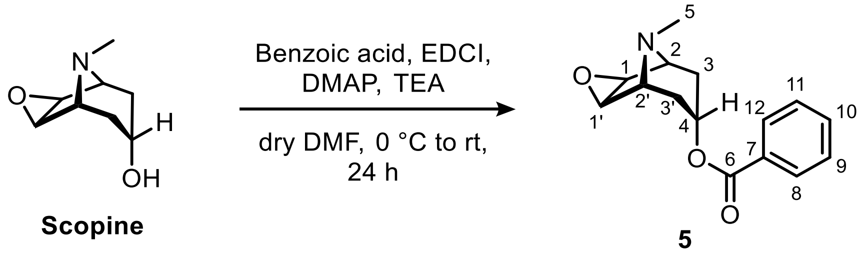


TEA (180 µL, 1.28 mmol, 2.0 equiv), EDCI (249 mg, 1.28 mmol, 2.0 equiv), DMAP (39 mg, 0.32 mmol, 0.5 equiv) and scopine (100 mg, 0.64 mmol, 1.0 equiv) were added to a solution of benzoic acid (156 mg, 1.28 mmol, 2.0 equiv) in dry DMF (6.4 mL) cooled at 0 °C. The mixture was stirred at room temperature for 24 h, then saturated NaOH 1M solution (10 mL) was added. The aqueous phase was extracted with AcOEt (3 x 20 mL), then the collected organic phases were dried over $Na_2SO_4$ and concentrated under reduced pressure. Compound **3** was obtained with 44% yield (74 mg) as a whitish solid after flash column chromatography purification (95:5 DCM/MeOH *v/v* + 1% TEA as eluent).

**$^1$H NMR (400 MHz, $CDCl_3$):** δ 7.96 (d, *J* = 7.0 Hz, 2H, Ph *H* 8 – 12), 7.55 (t, *J* = 8.1 Hz, 1H, Ph *H* 10), 7.42 (t, *J* = 7.6 Hz, 2H, Ph *H* 9 - 11), 5.27 (t, *J* = 5.6 Hz, 1H, C*H* 4), 3.77 (s, 2H, C*H* 1 - 1'), 3.27 (s, 2H, C*H* 2 – 2'), 2.59 (s, 3H, $CH_3$ 5), 2.31 (dt, *J* = 15.5, 5.6 Hz, 2H, axial $CH_2$ 3 - 3'), 1.76 (d, *J* = 15.5 Hz, 2H, equatorial $CH_2$ 3 - 3').

**$^{13}$C NMR (100 MHz, CDCl$_3$)** δ 165.7 (C=O 6), 133.5 (Ph *C*H 10), 130.7 (Ph *C* 7), 129.8 (Ph *C*H 8 - 12), 128.9 (Ph *C*H 9 - 11), 66.9 (*C*H 4), 58.9 (*C*H 2 - 2'), 56.8 (*C*H 1 - 1'), 43.2 (*C*H$_3$ 5), 31.8 (*C*H$_2$ 3 - 3').

**MS (ESI)** *m/z:* [M+Na]$^+$ Calcd for $C_{15}H_{17}NO_3Na$ 282.11; Found 282.11

See spectra

## 2.13 Synthesis of 6

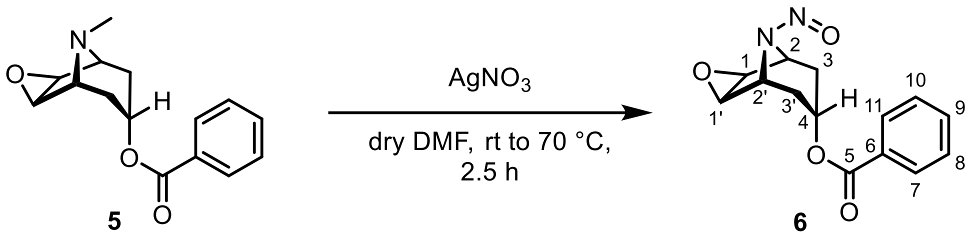


$AgNO_2$ (234 mg, 1.52 mmol, 8.0 equiv) was added to a solution of **5** (50 mg, 0.19 mmol, 1.0 equiv) in dry DMF (3.8 mL) at room temperature. The reaction mixture was left stirring at 70 °C in an oil bath for 2.5 h, then concentrated under reduced pressure. The resulting solid was dissolved in DCM (20 mL) and washed with water (10 mL), then the organic phase was dried over $Na_2SO_4$ and concentrated under reduced pressure. Compound **6** was obtained with 26% yield (15 mg) as a whitish solid after flash column chromatography purification (7:3 *n*-hex/AcOEt *v/v* as eluent).

**$^1$H NMR (400 MHz, CDCl$_3$):** δ 7.98 (d, *J* = 7.0 Hz, 2H, Ph *H* 7 – 11), 7.61 (t, *J* = 7.5 Hz, 1H, Ph *H* 9), 7.47 (t, *J* = 7.8 Hz, 2H, Ph *H* 8 – 10), 5.41 (t, *J* = 4.9 Hz, 1H, C*H* 4), 5.33 – 5.28 (m, 1H, C*H* 2'), 5.17 (dd, *J* = 4.0, 2.0 Hz, 1H, C*H* 2), 3.80 (d, *J* = 3.3 Hz, 1H, C*H* 1'), 3.74 (d, *J* = 3.3 Hz, 1H, C*H* 1), 2.55 (td, *J* = 15.4, 4.9, 1H, axial C*H*$_2$ 3), 2.31 – 2.19 (m, 2H, equatorial C*H*$_2$ 3 – axial C*H*$_2$ 3'), 2.06 (dd, *J* = 15.4, 2.0 Hz, 1H, equatorial C*H*$_2$ 3').

**$^{13}$C NMR (100 MHz, CDCl$_3$):** δ 165.2 (C=O 5), 133.7 (Ph *C*H 9), 129.8 (Ph *C* 6), 129.5 (Ph *C*H 7 – 11), 128.8 (Ph *C*H 8 – 10), 66.3 (*C*H 4), 57.4 (*C*H 2), 51.8 (*C*H 1'), 51.2 (*C*H 1), 50.4 (*C*H 2'), 33.3 (*C*H$_2$ 3), 31.1 (*C*H$_2$ 3').

**MS (ESI)** *m/z:* [M+Na]$^+$ Calcd for $C_{14}H_{14}N_2O_4Na$ 297.08; Found 297.09

See spectra

## 2.14 Synthesis of 7

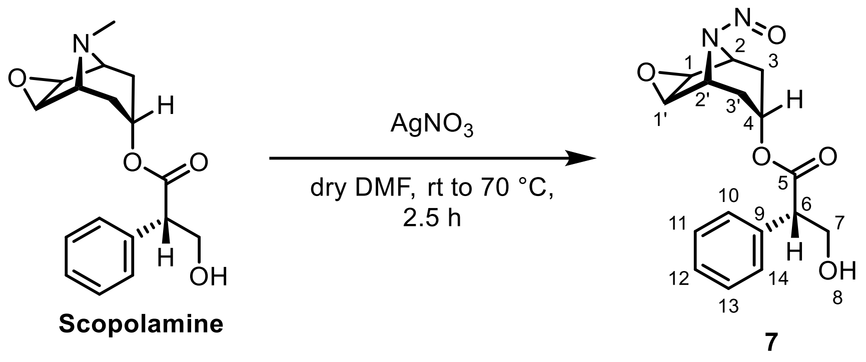

AgNO$_2$ (197 mg, 1.28 mmol, 8.0 equiv) was added to a solution of scopolamine (50 mg, 0.16 mmol, 1.0 equiv) in dry DMF (3.2 mL) at room temperature. The reaction mixture was left stirring at 70 °C in an oil bath for 2.5 h, then concentrated under reduced pressure. The resulting solid was dissolved in DCM (20 mL) and washed with water (10 mL), then the organic phase was dried over Na$_2$SO$_4$ and concentrated under reduced pressure. Compound **7** was obtained with 24% yield (12 mg) as a yellowish solid after flash column chromatography purification (7:3 *n*-hex/AcOEt *v/v* as eluent).

**$^1$H NMR (400 MHz, CDCl$_3$)** δ 7.40 – 7.19 (m, 10H, Ph *H* rotamers), 5.16 – 5.09 (m, 3H, C*H* 2' – 4 rotamers), 5.00 (s, 2H, C*H* 2 rotamers), 4.85 (dd, *J* = 4.1, 1.9 Hz, 1H, C*H* 2' rotamer), 4.17 (t, *J* = 9.8 Hz, 2H, C*H*$_2$ 7 rotamers), 3.90 – 3.71 (m, 4H, C*H*$_2$ 7 – C*H* 6 rotamers), 3.39 (d, *J* = 3.4 Hz, 1H, C*H* 1 rotamer), 3.33 (d, *J* = 3.4 Hz, 1H, C*H* 1' rotamer), 2.61 (d, *J* = 3.4 Hz, 1H, C*H* 1' rotamer), 2.57 (d, *J* = 3.4 Hz, 1H, C*H* 1 rotamer), 2.38 (dt, *J* = 15.5, 4.5 Hz, 1H, axial C*H*$_2$ 3 rotamer), 2.28 (dt, *J* = 15.7, 4.9 Hz, 1H, axial C*H*$_2$ 3' rotamer), 2.14 – 2.02 (m, 2H, equatorial C*H*$_2$ 3 – axial C*H*$_2$ 3' rotamers), 1.98 (dt, *J* = 15.6, 4.3 Hz, 1H, axial C*H*$_2$ 3 rotamer), 1.90 – 1.82 (m, 2H, equatorial C*H*$_2$ 3' rotamers), 1.65 (d, *J* = 14.3 Hz, 1H, equatorial C*H*$_2$ 3 rotamer).

**$^{13}$C NMR (100 MHz, CDCl$_3$)** δ 171.6 (C=O 5 rotamers), 135.4 (Ph CH 9 rotamer), 135.4 (Ph *C*H 9 rotamer), 129.2 (Ph *C*H 11 – 13 rotamers), 128.3 (Ph *C*H 12 rotamers), 128.0 (Ph *C*H 10 – 14 rotamers), 66.3 (*C*H 4 rotamers), 63.9 (*C*H$_2$ 7 rotamer), 63.9 (*C*H$_2$ 7 rotamer), 57.3 (*C*H 2 rotamer), 57.1 (*C*H 2' rotamer), 54.2 (*C*H 6 rotamers), 51.4 (*C*H 1 rotamer), 50.9 (*C*H 1' rotamer), 50.9 (*C*H 1' rotamer), 50.4 (*C*H 1 rotamer), 50.2 (*C*H 2' rotamer), 50.1 (*C*H 2 rotamer), 33.0 (*C*H$_2$ 3 rotamer), 32.7 (*C*H$_2$ 3' rotamer), 30.7 (*C*H$_2$ 3' rotamer), 30.5 (*C*H$_2$ 3 rotamer).

**MS (ESI)** *m/z:* [M+Na]$^+$ Calcd for $C_{16}H_{18}N_2O_5Na$ 341.11; Found 341.11

See spectra

# 3. SPECTROSCOPIC DATA

$^{1}$H NMR (400 MHz, $CDCl_3$) of **2** (see procedure)

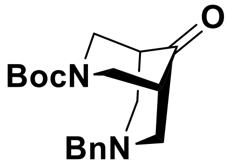

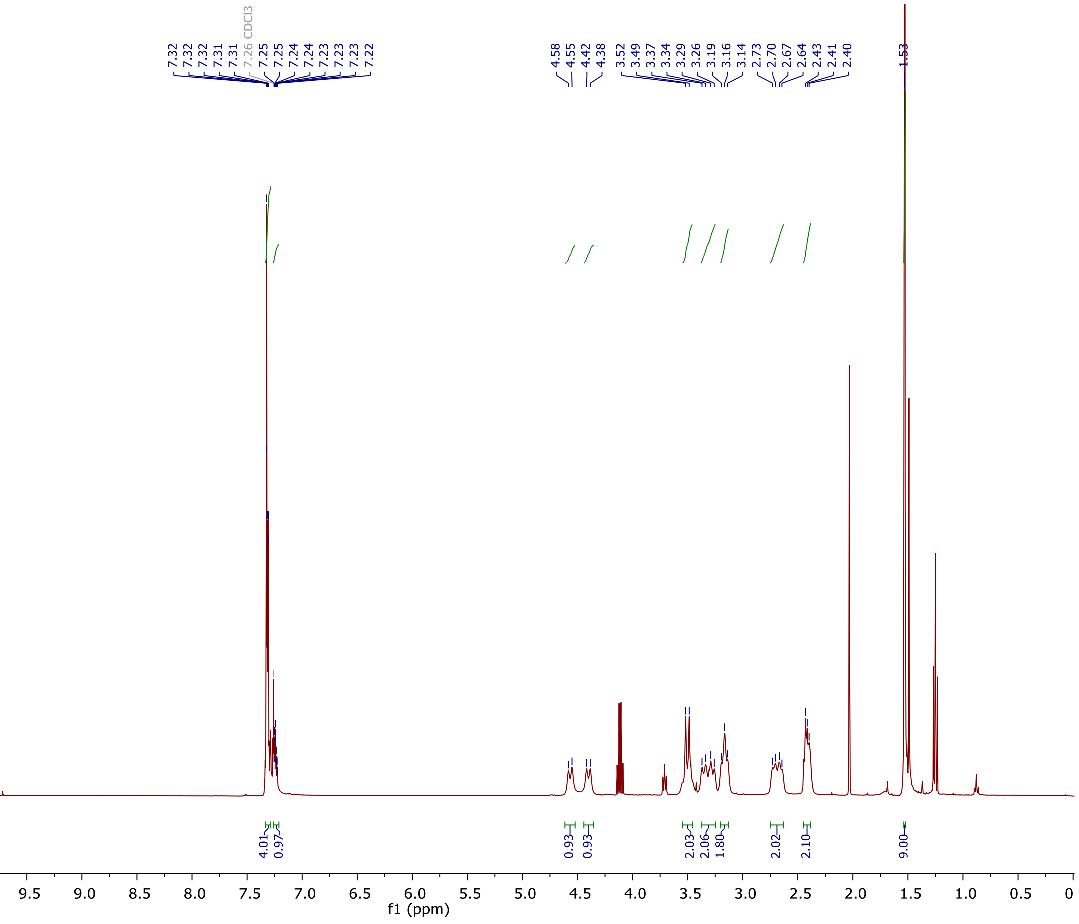

$^{1}$H NMR (400 MHz, $CDCl_3$) of **Boc-Bn** (see procedure)

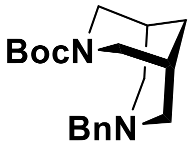


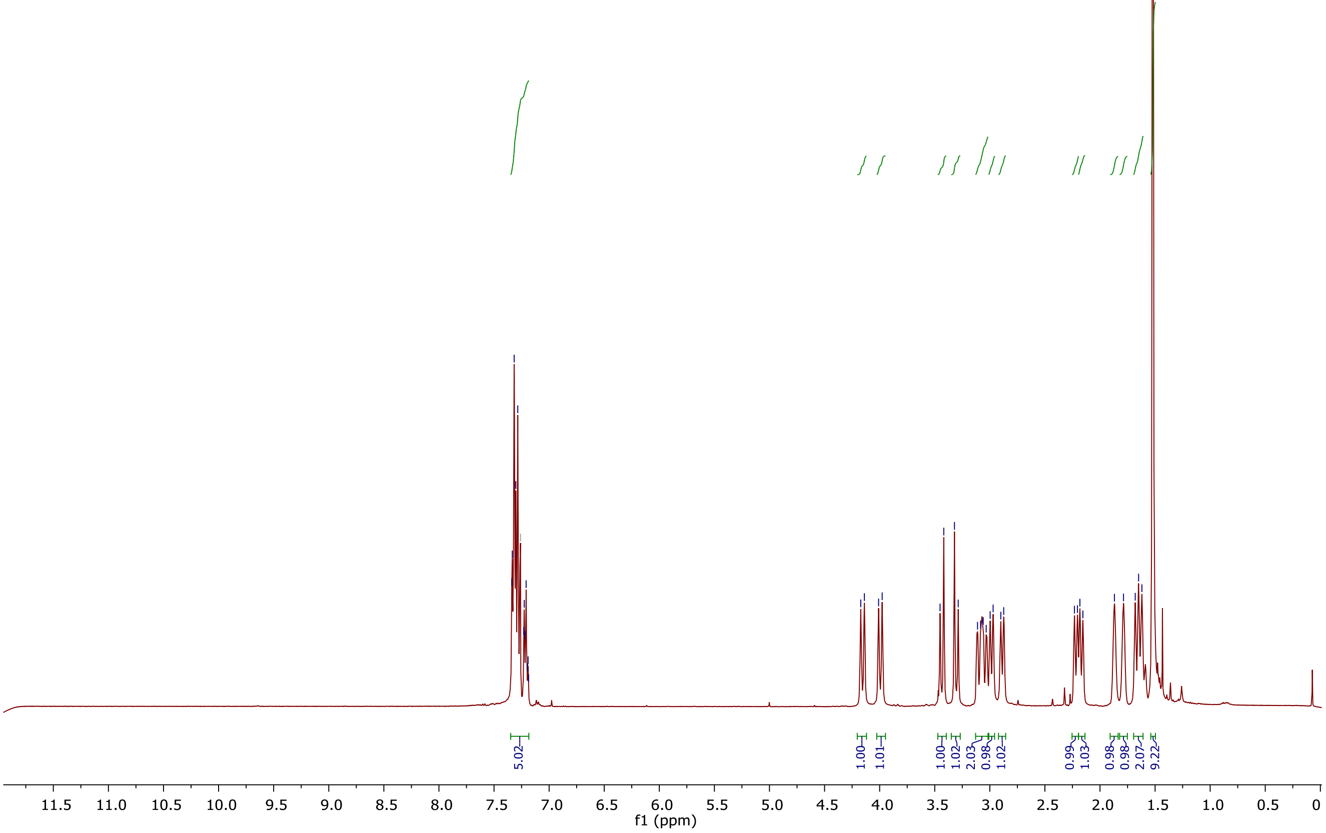

$^{1}$H NMR (400 MHz,$CD_3OD$) of **Boc-Bn** (see procedure)

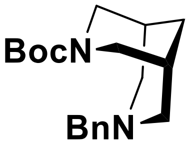


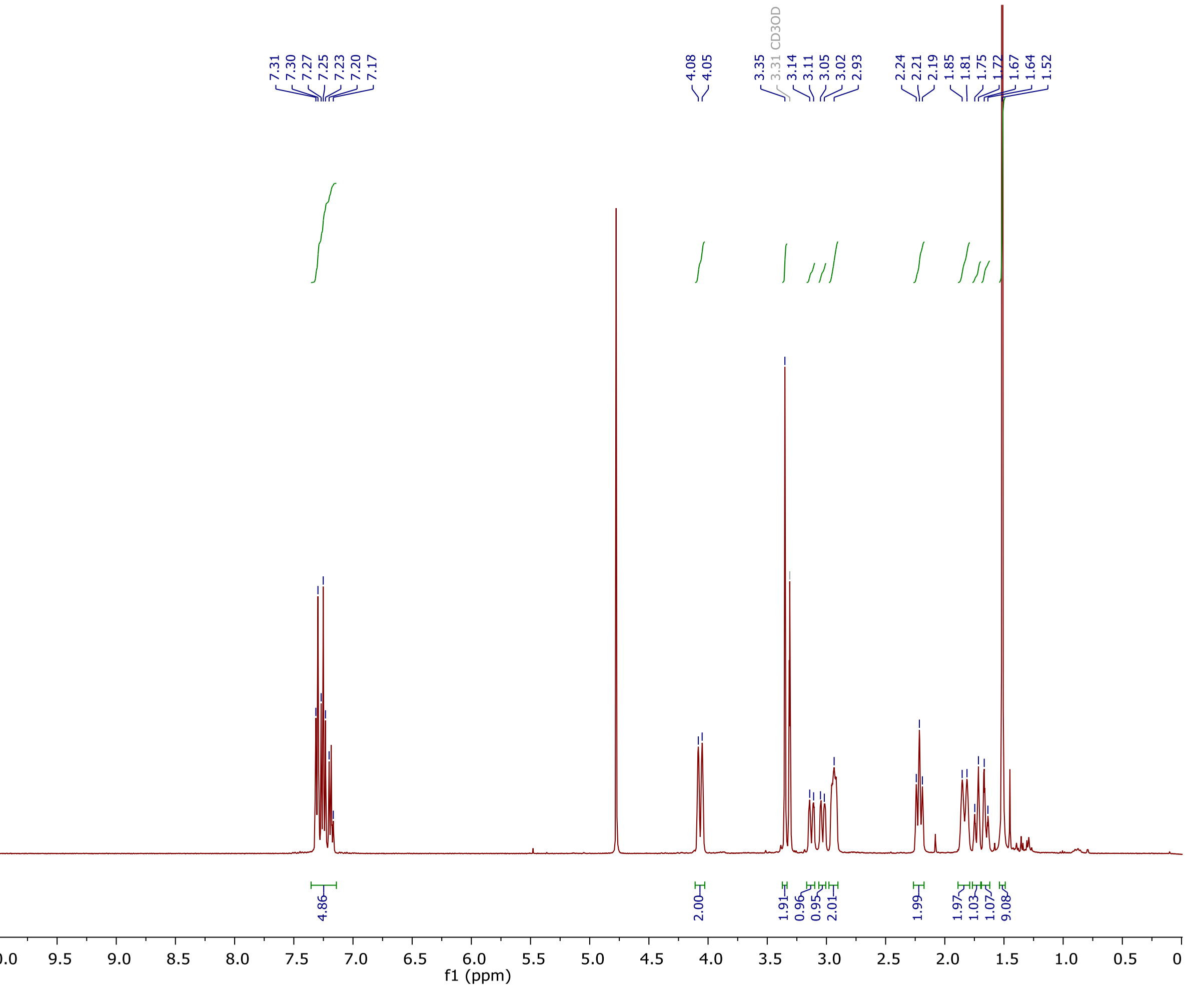

$^{13}$C NMR (400 MHz,$CDCl_3$) of **Boc-Bn** (see procedure)

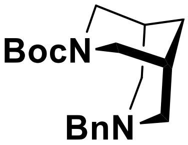


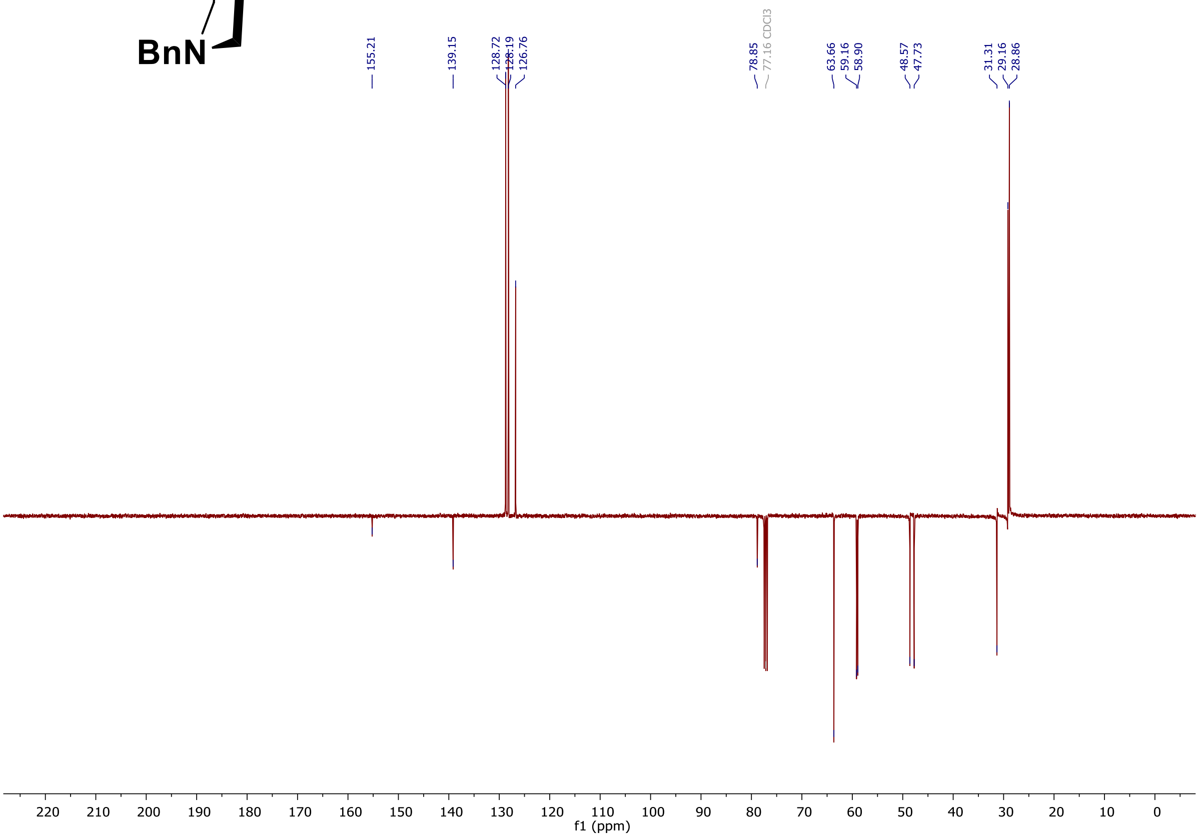

COSY of **Boc-Bn** (see procedure)

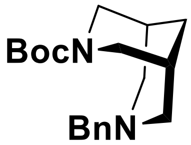


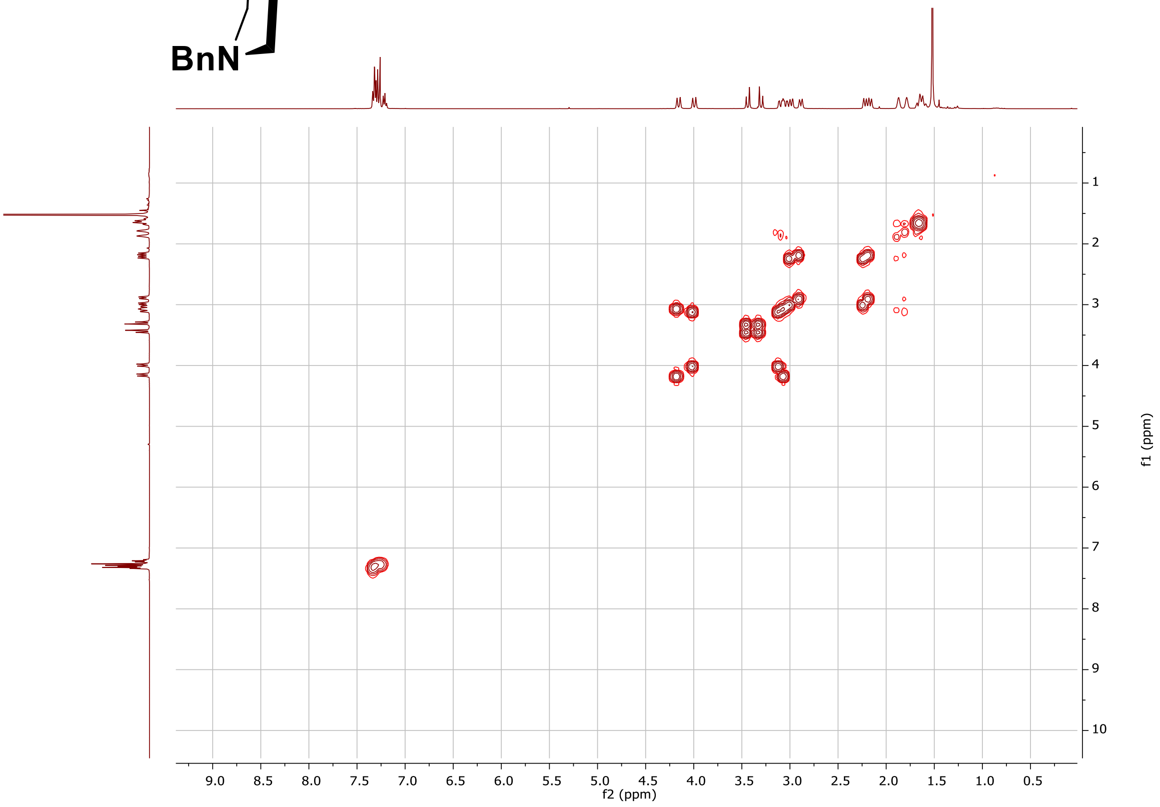

HSQC of **Boc-Bn** (see procedure)

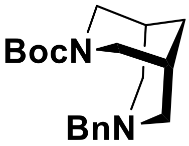


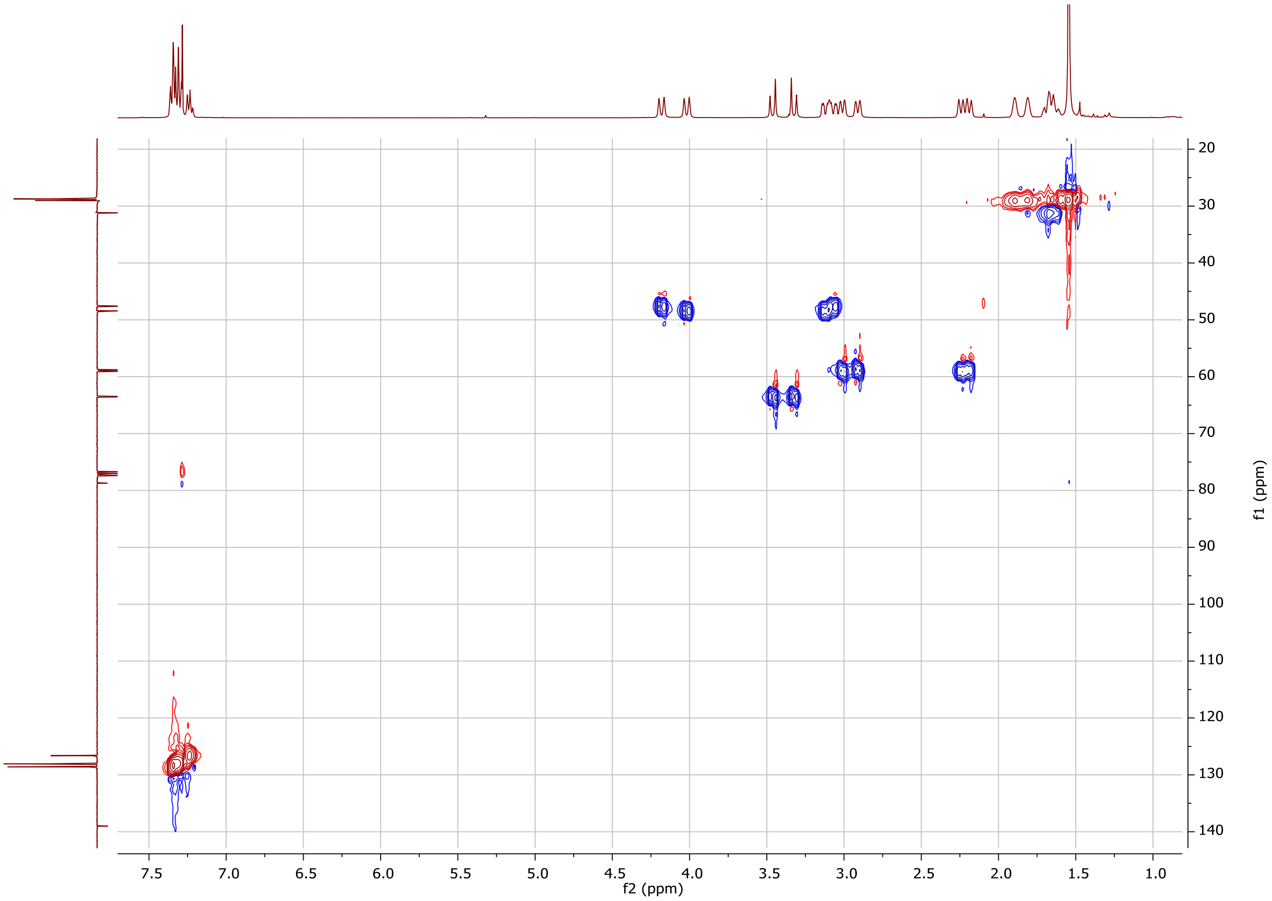

HMBC of **Boc-Bn** (see procedure)

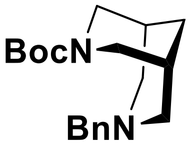


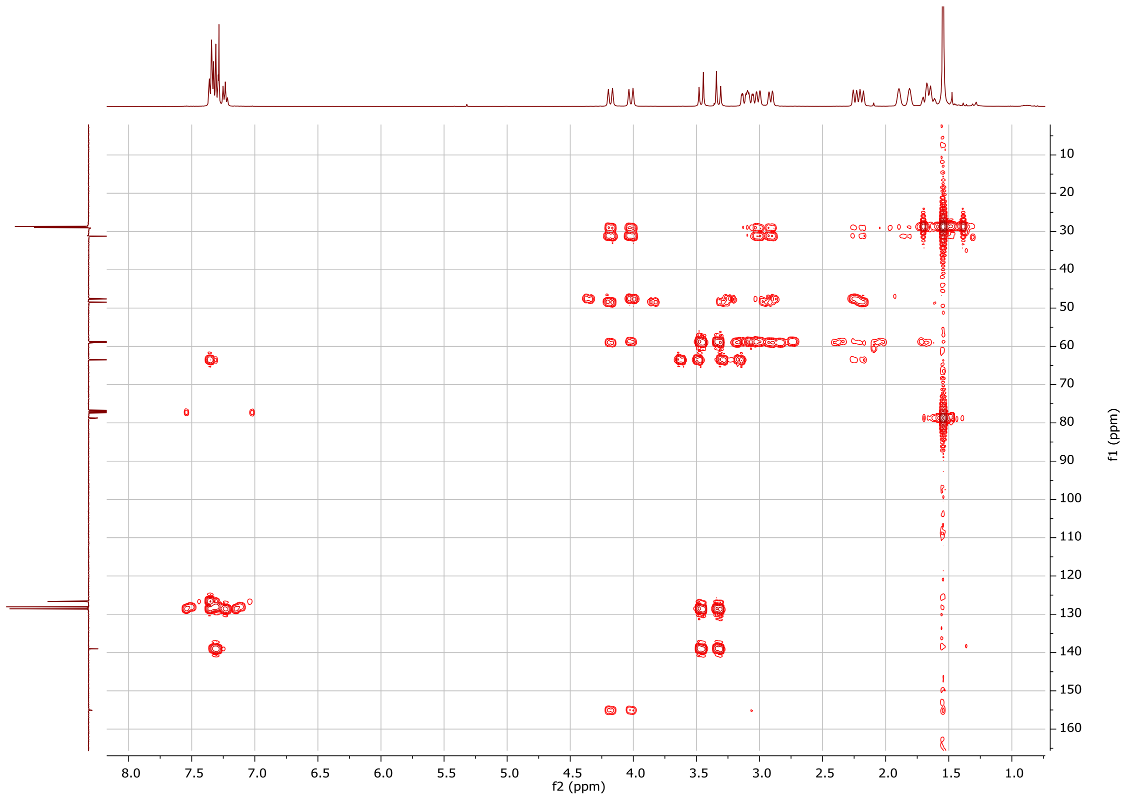

$^{1}$H NMR (400 MHz, $CD_3OD$) of **Boc-H** (see procedure)

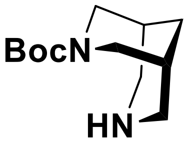


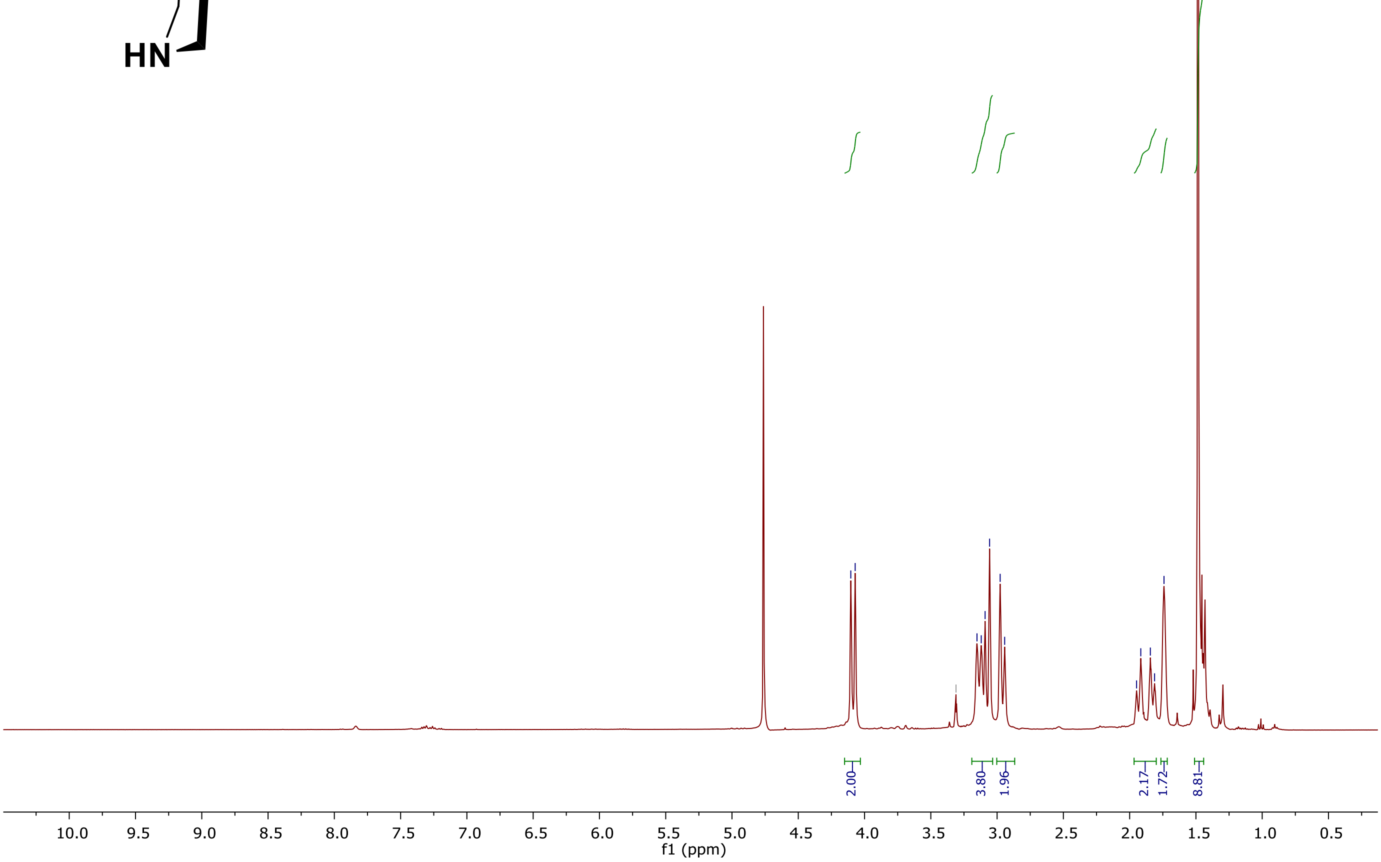

$^{1}$H NMR (400 MHz,CDCl₃) of **Boc-Cl** (see procedure)

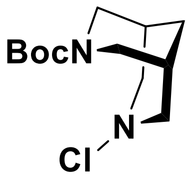


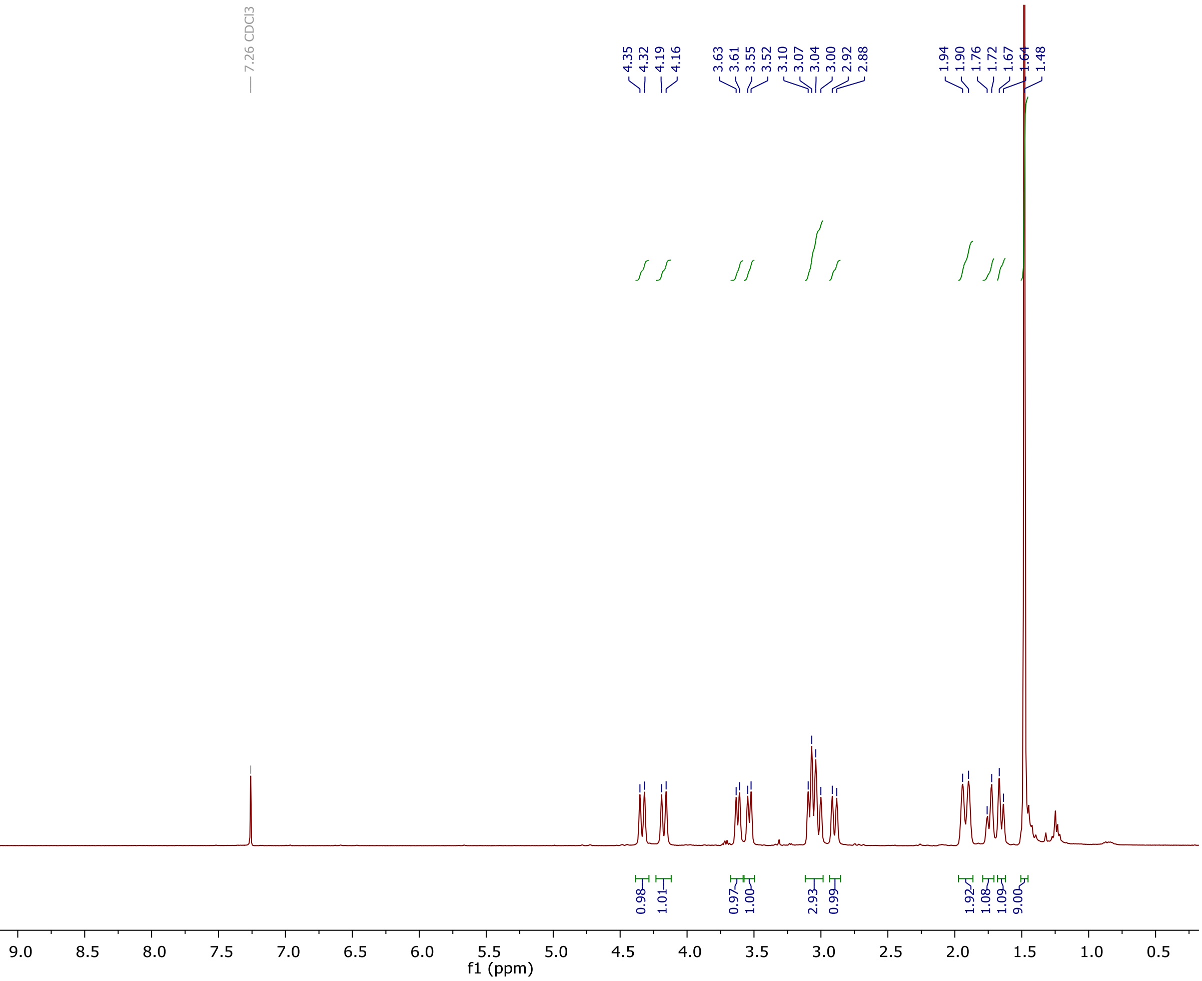

$^{13}C$ NMR (400 MHz,$CDCl_3$) of **Boc-Cl** (see procedure)

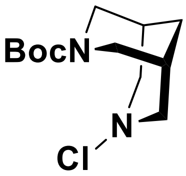


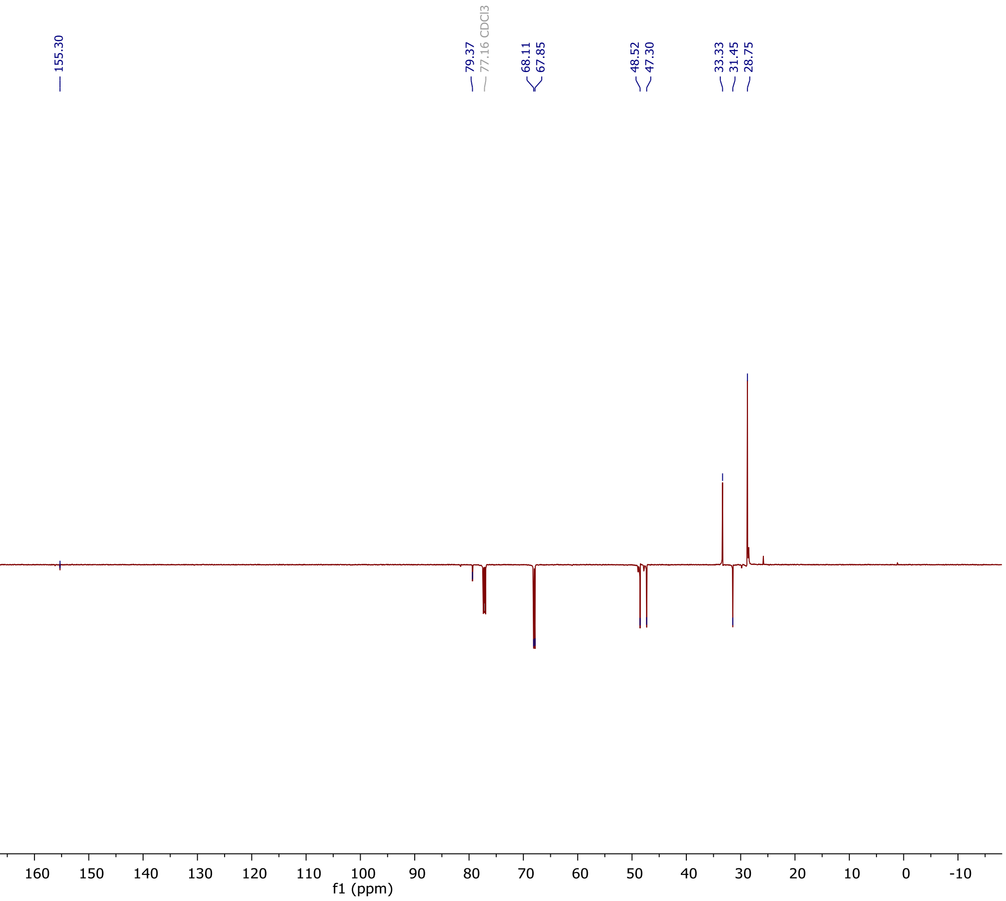

COSY of **Boc-Cl** (see procedure)

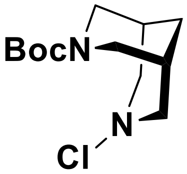


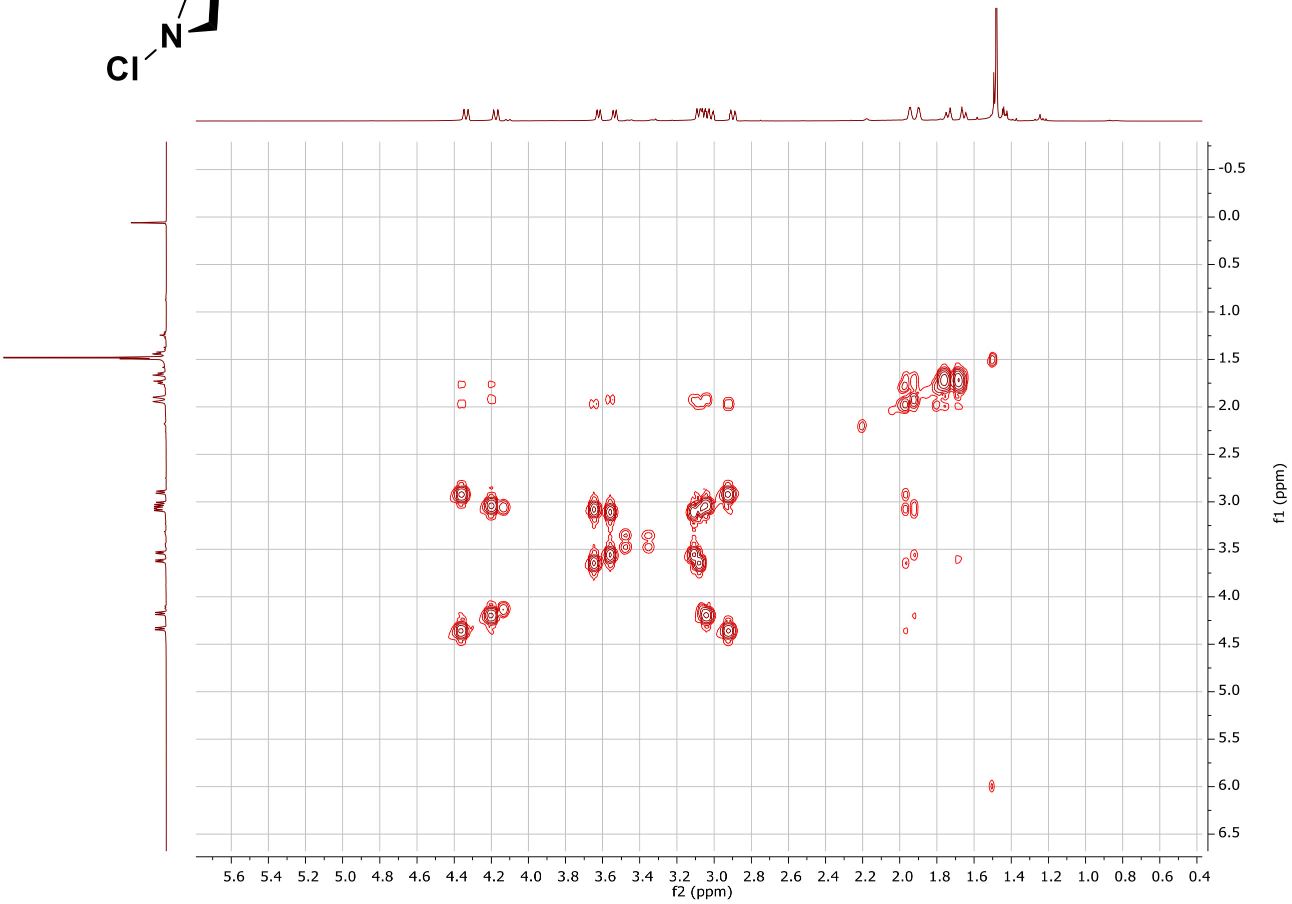

HSQC of **Boc-Cl** (see procedure)

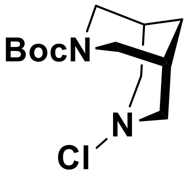


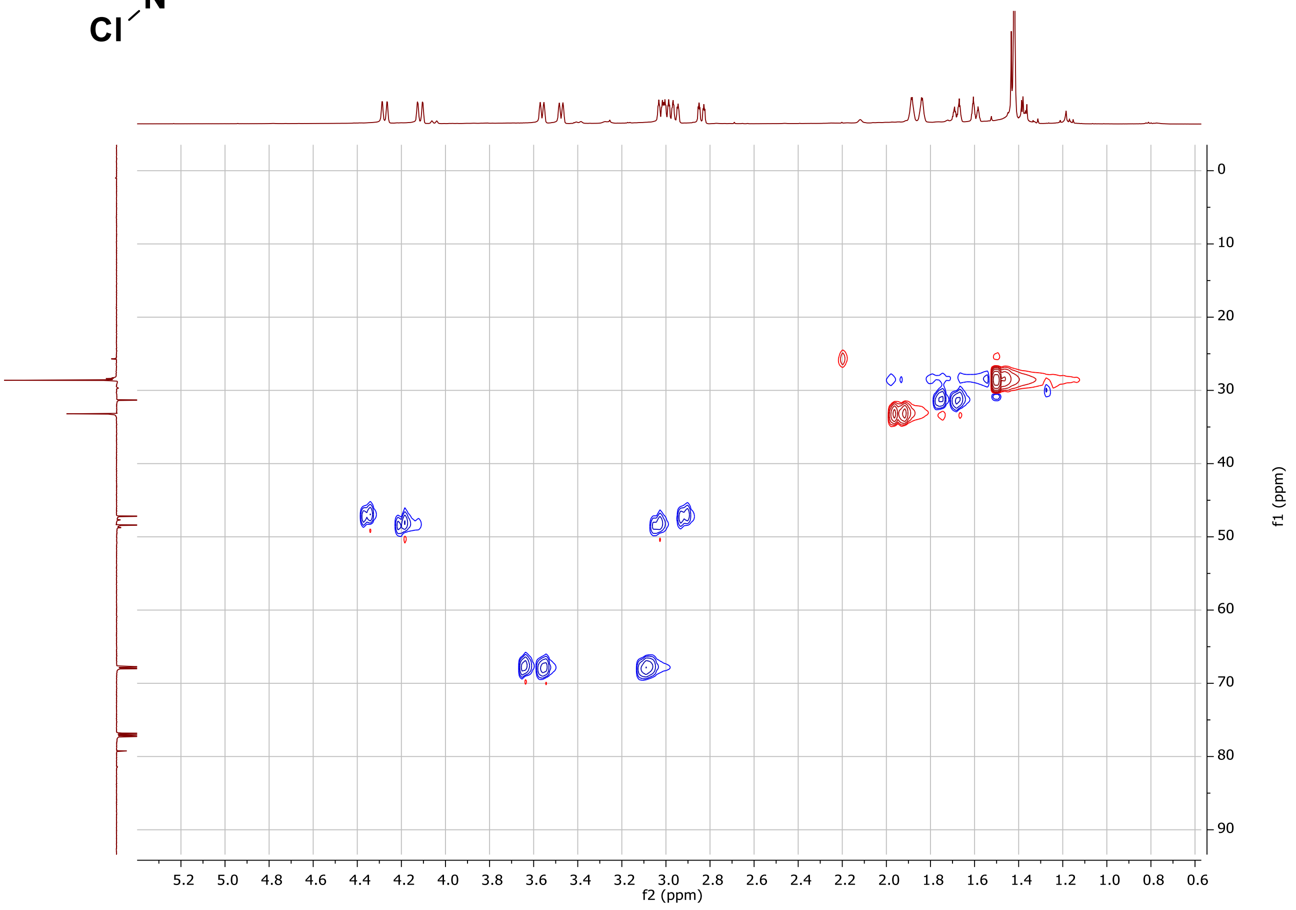

HMBC of **Boc-Cl** (see procedure)

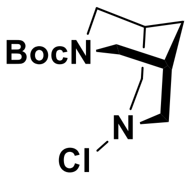


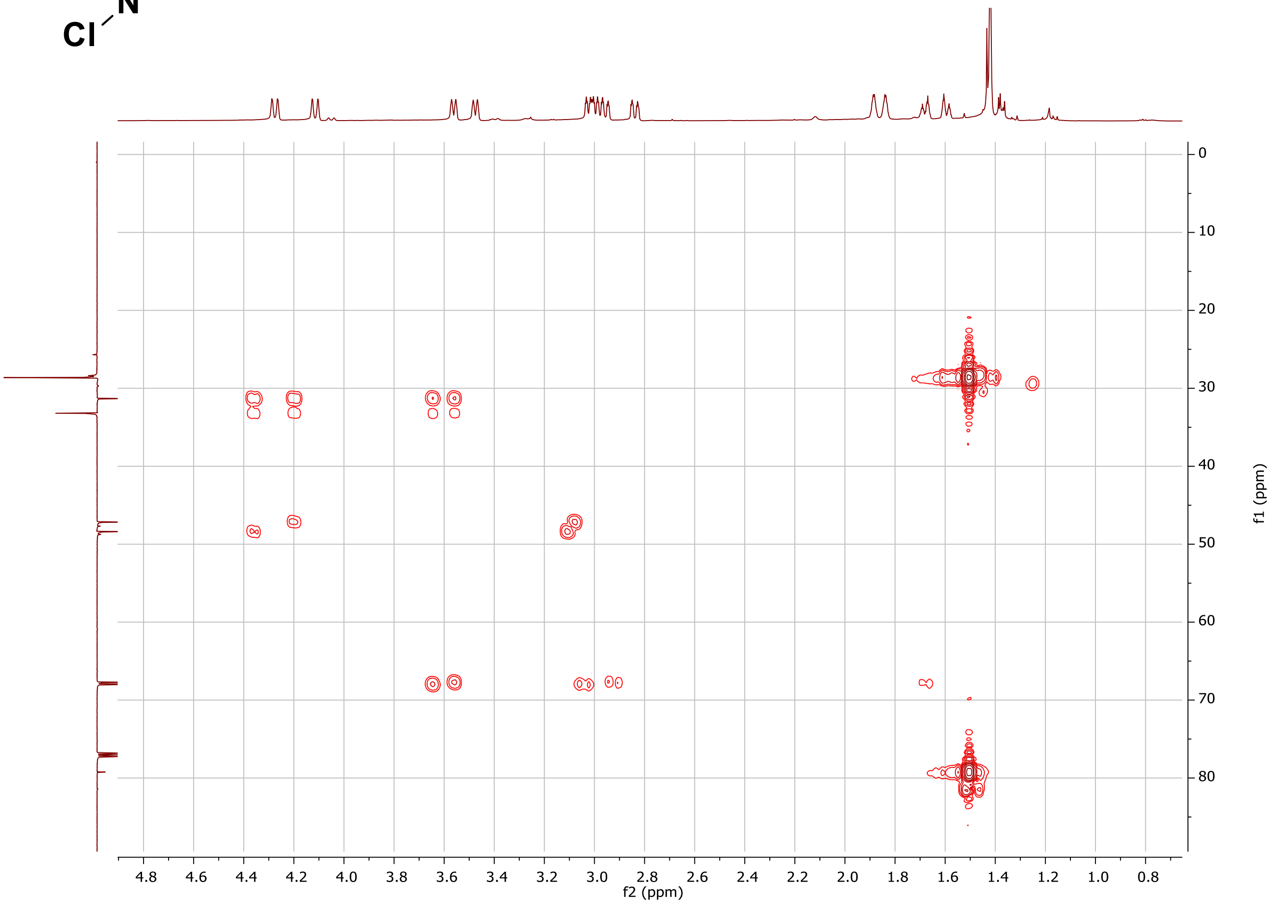

$^{1}$H NMR (400 MHz, CDCl3) of **Boc-PEA** (see procedure)

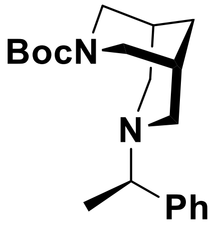


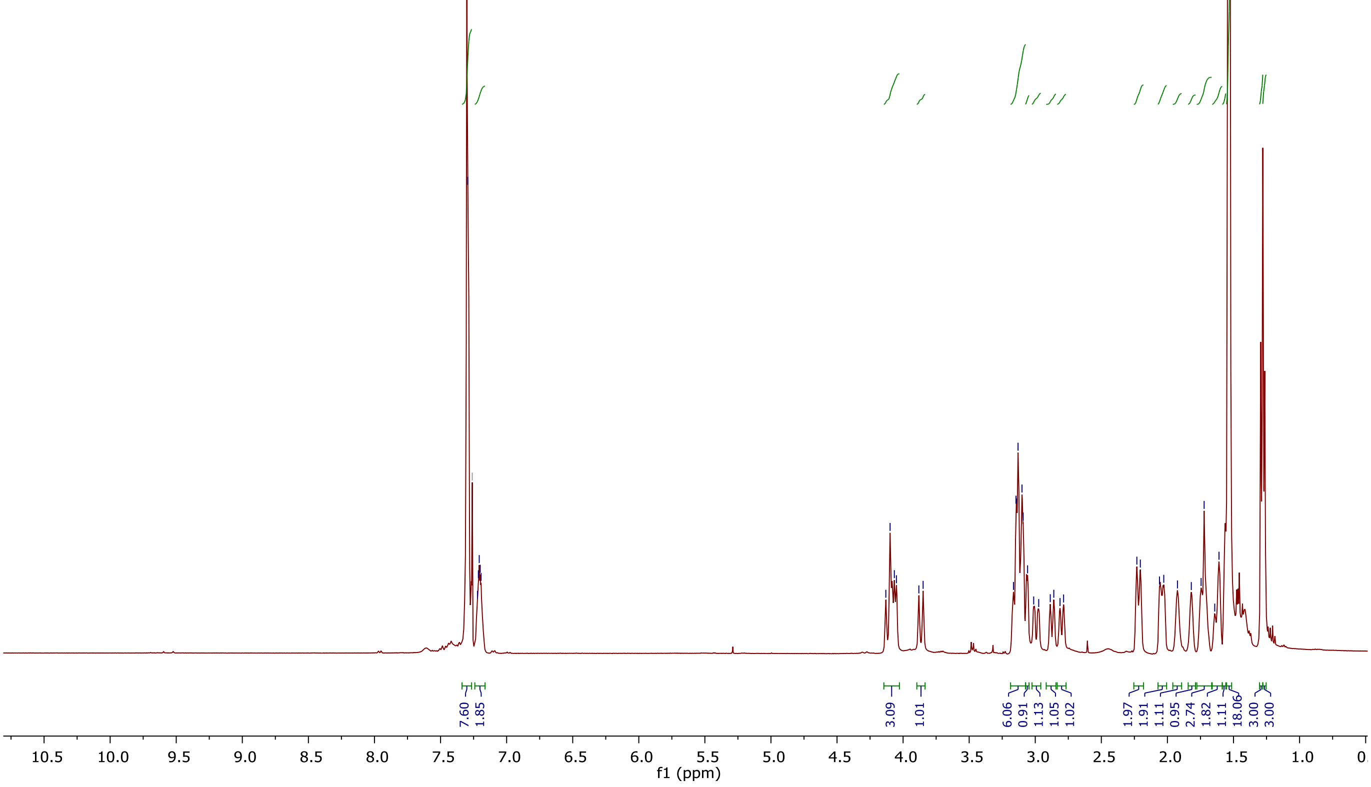

$^{13}C$ NMR (400 MHz,$CDCl_3$) of **Boc-PEA** (see procedure)

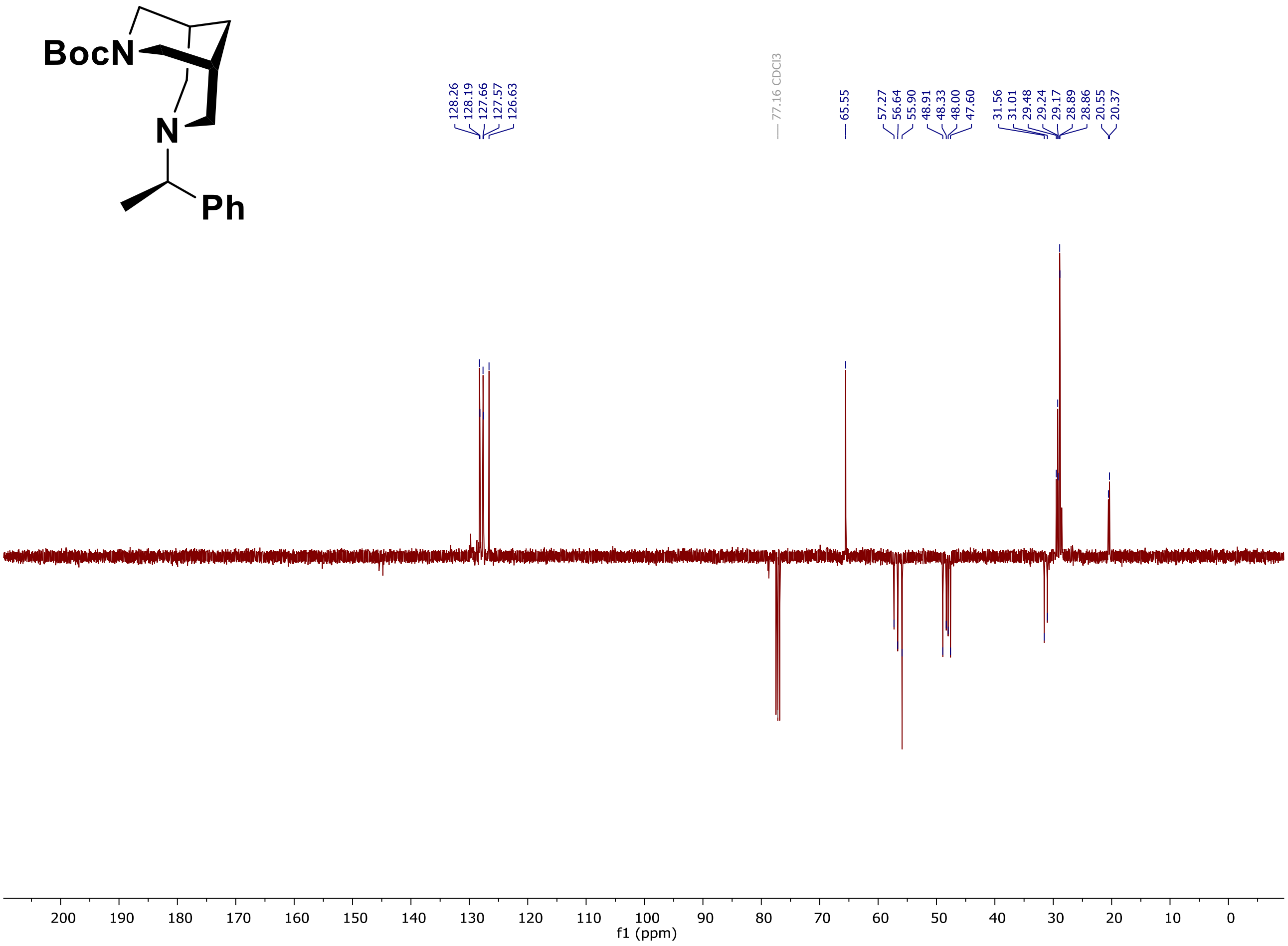

COSY of **Boc-PEA** (see procedure)

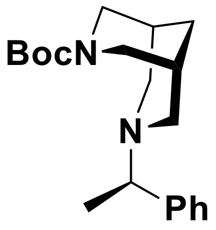


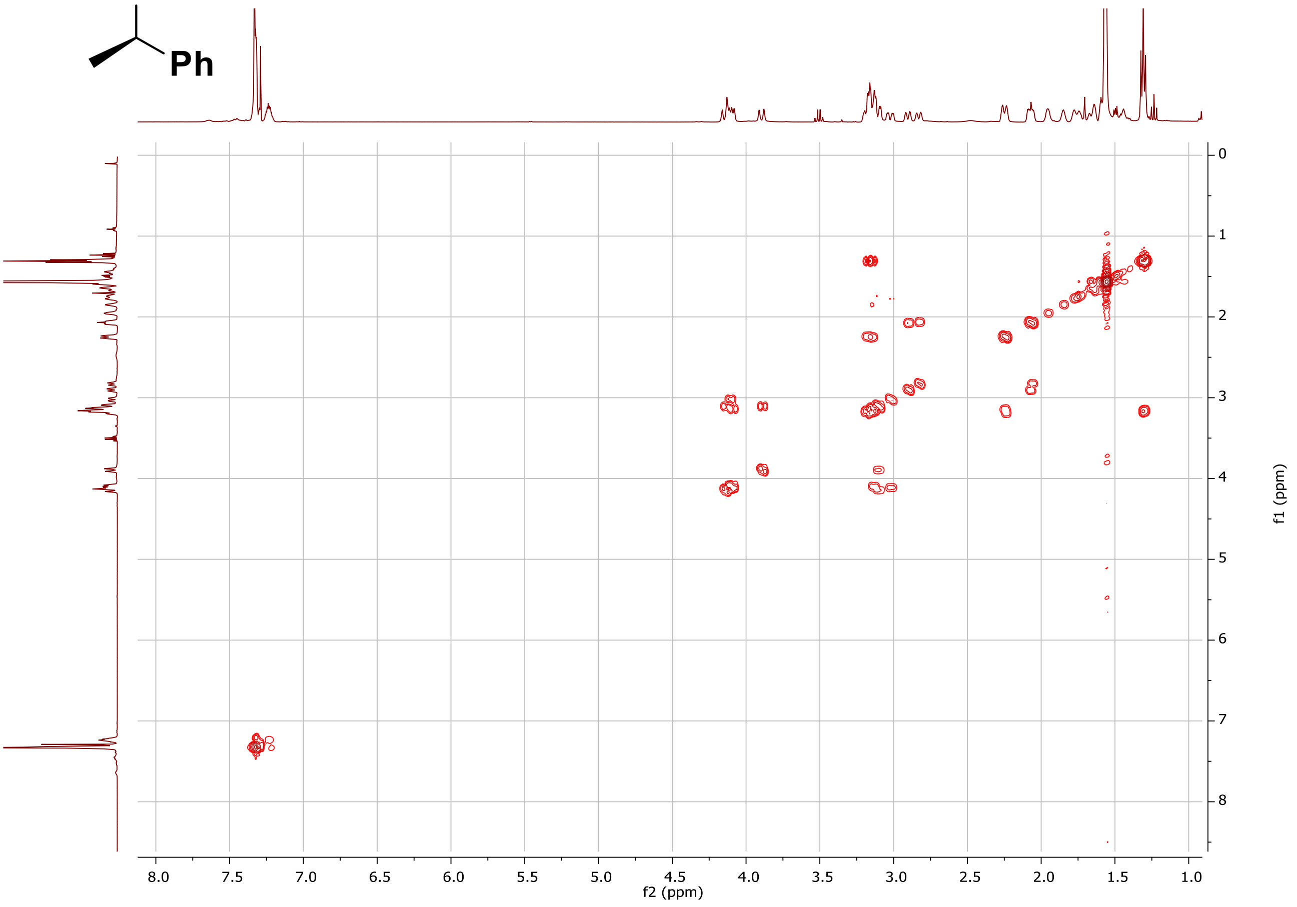

HSQC of **Boc-PEA** (see procedure)

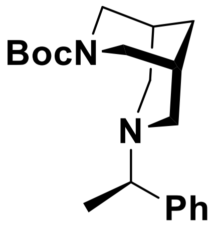


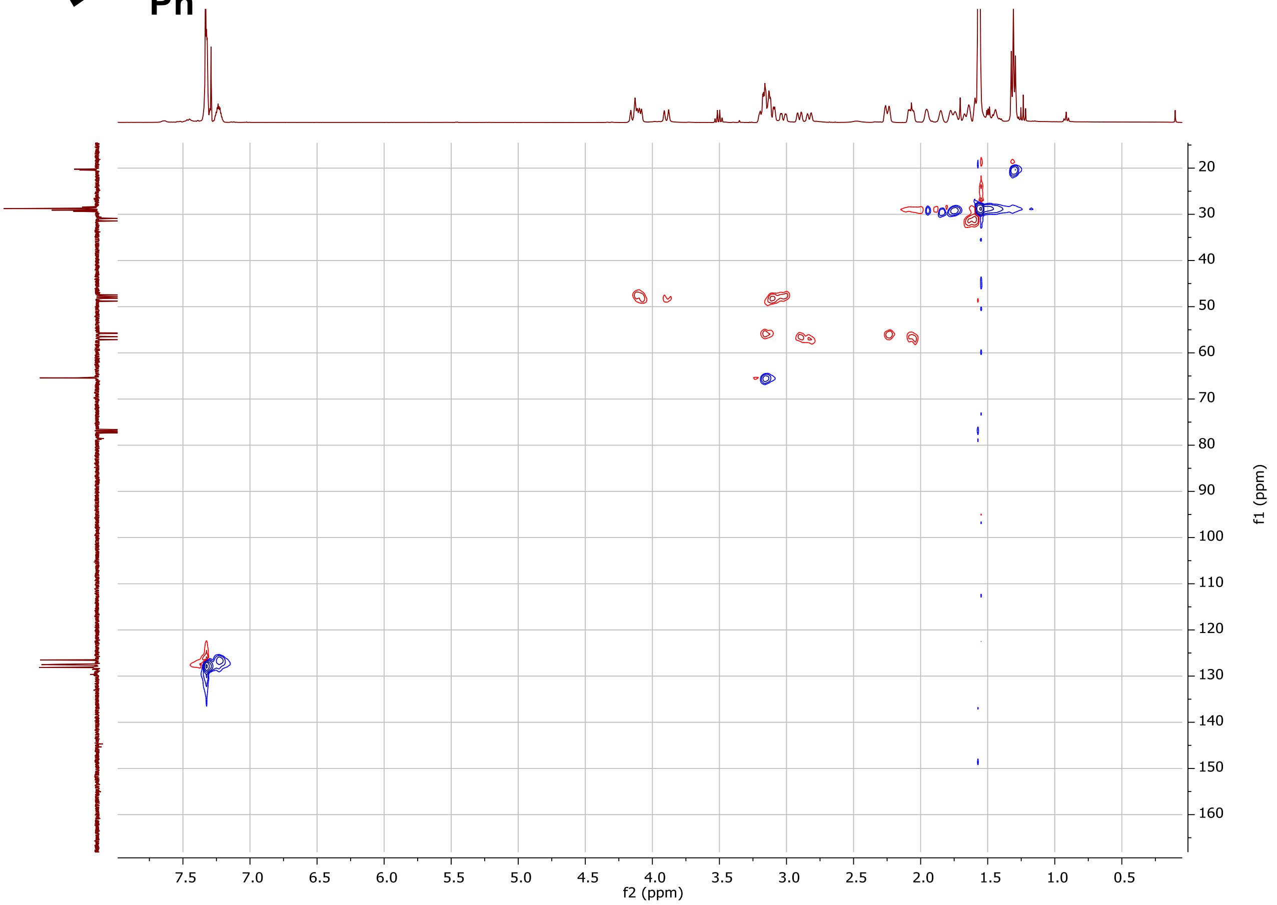

HMBC of **Boc-PEA** (see procedure)

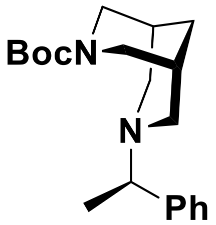


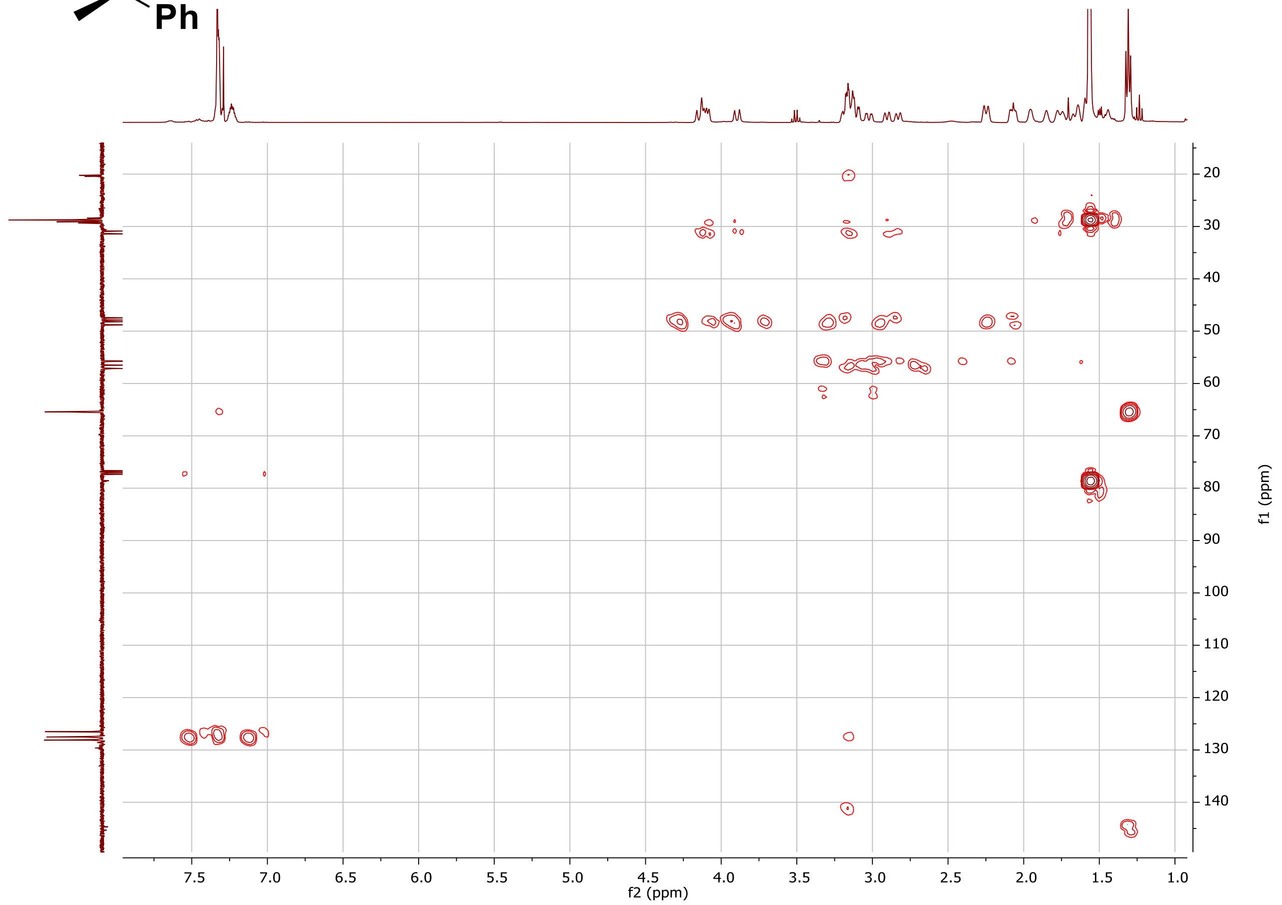

VT NMR (400 MHz, DMSO) of **Boc-PEA** (see procedure)

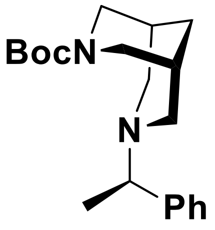


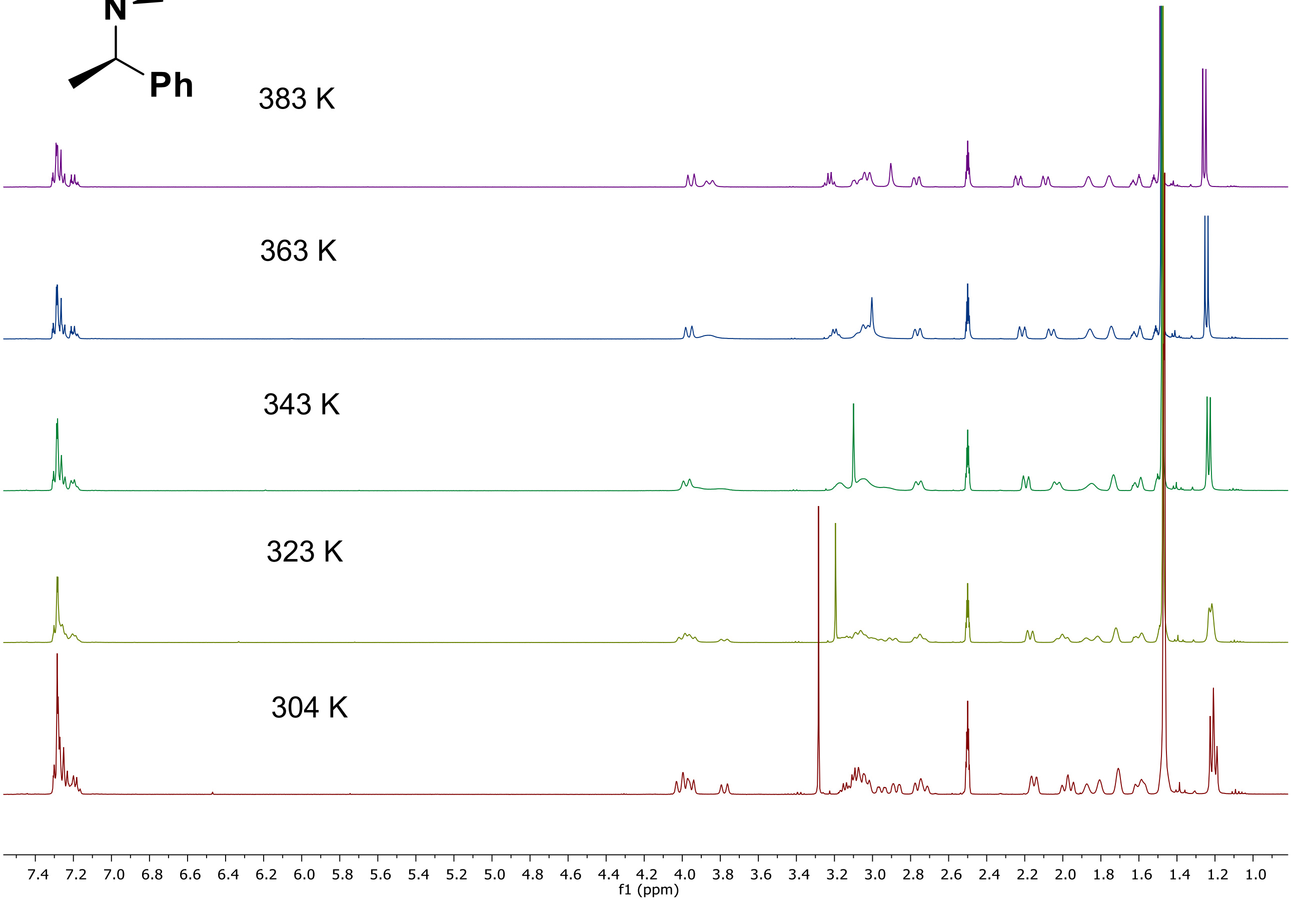

$^1$H NMR (400 MHz, $CDCl_3$) of **Boc-Me** (see procedure)

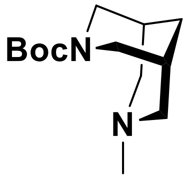

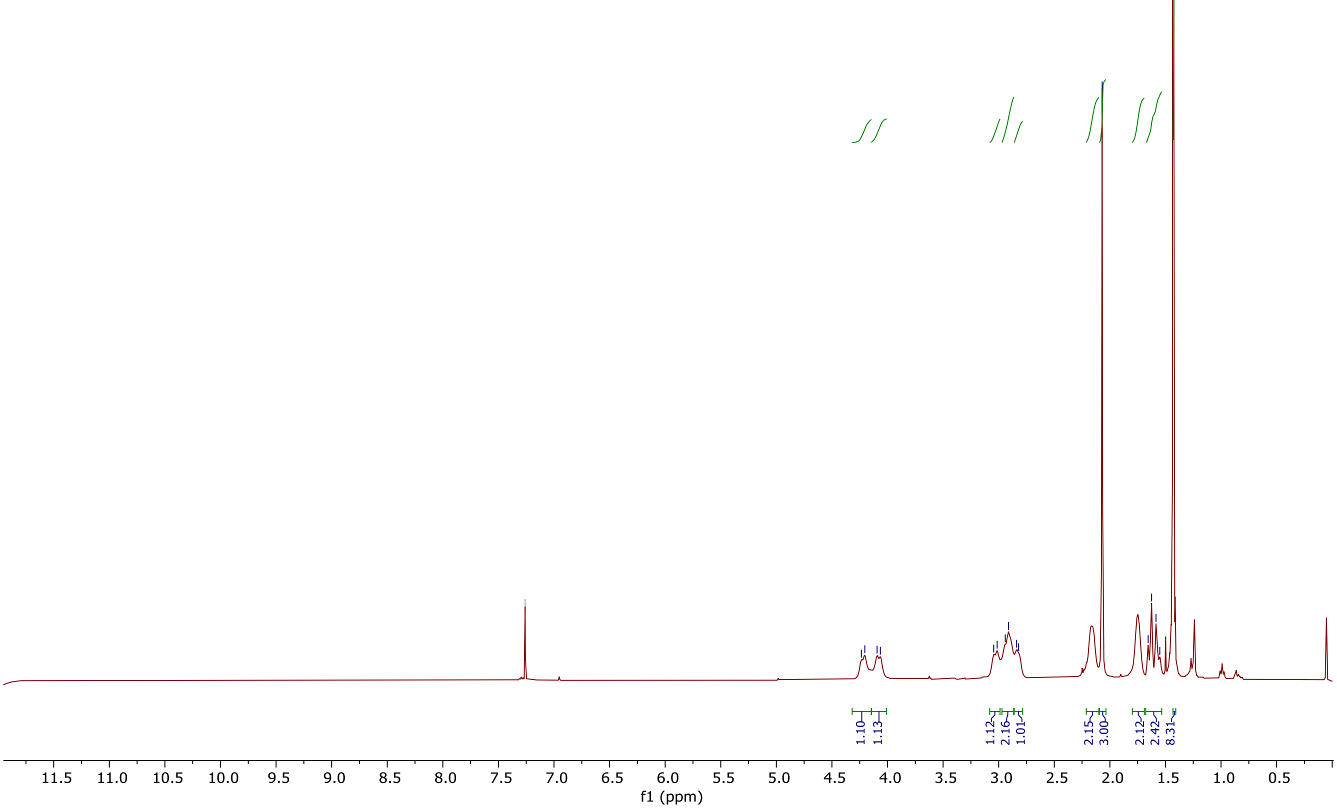

$^{13}C$ NMR (400 MHz,$CDCl_3$) of **Boc-Me** (see procedure)

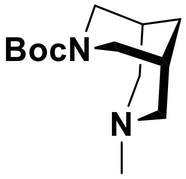


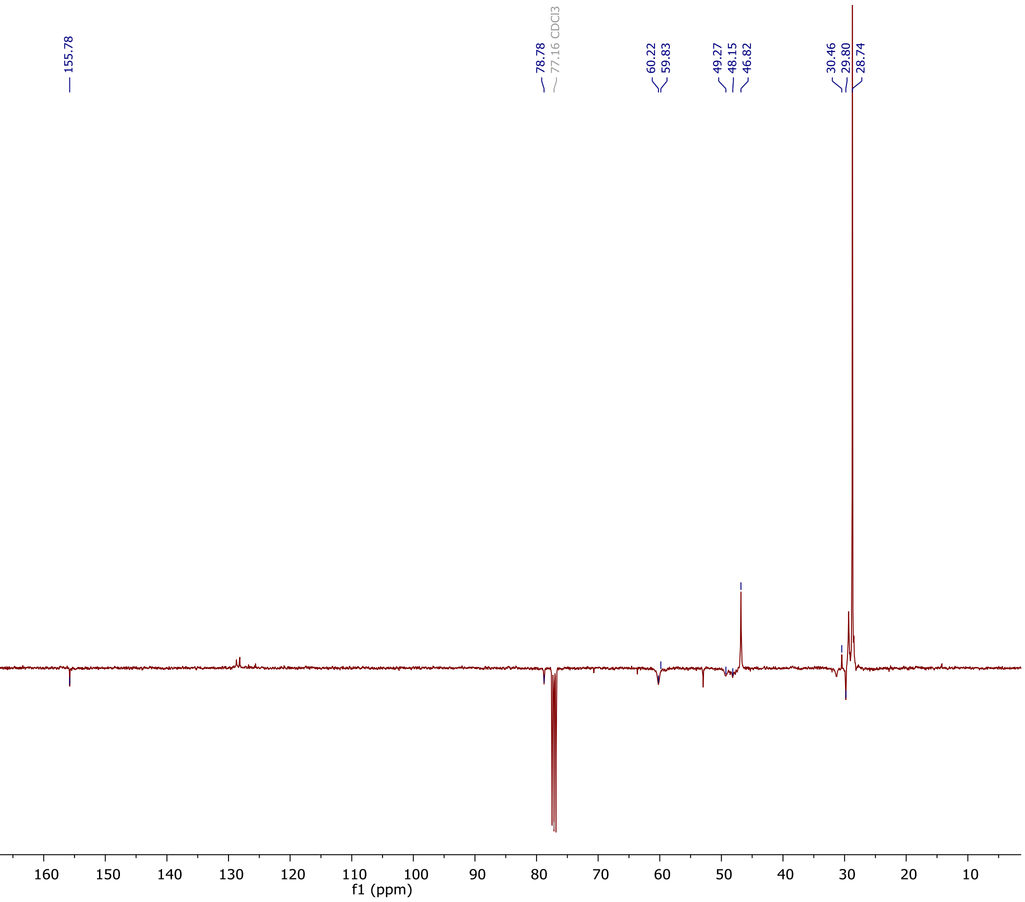

COSY of **Boc-Me** (see procedure)

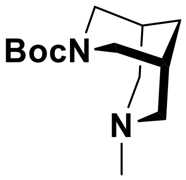


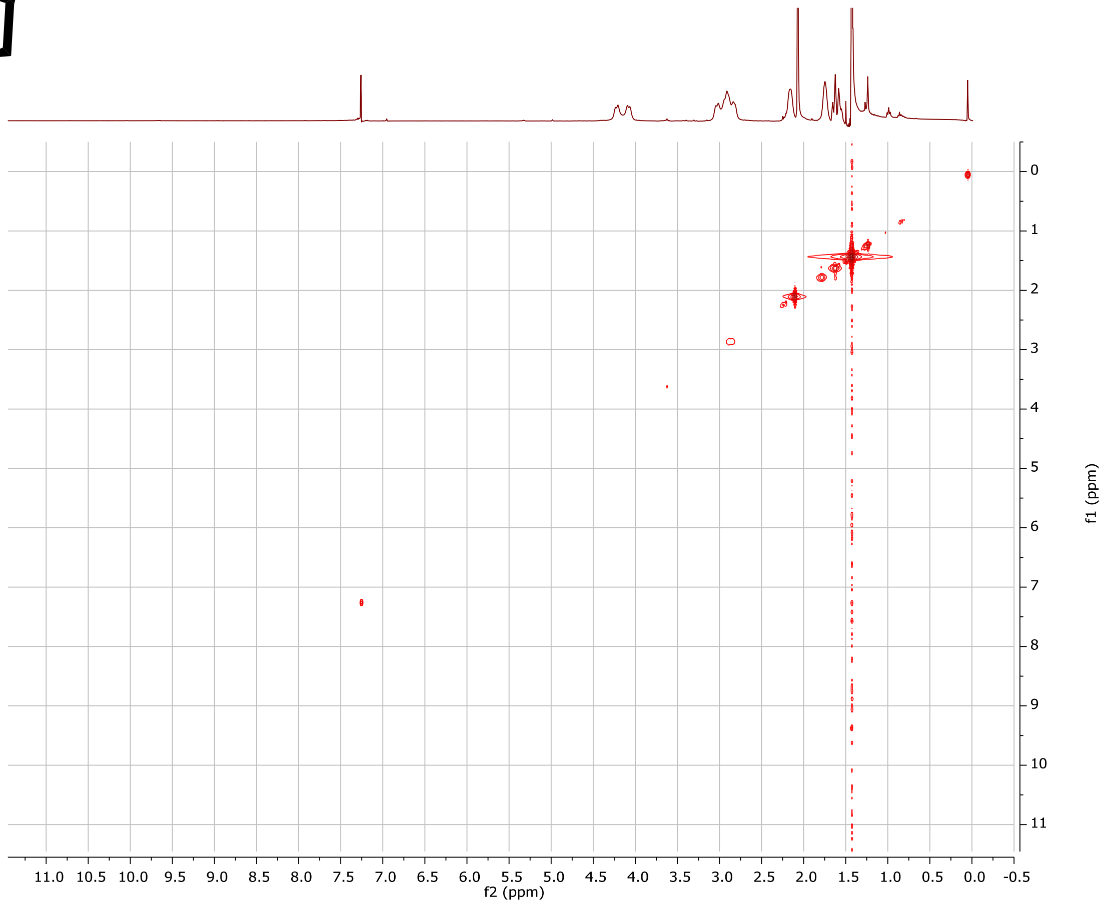

HSQC of **Boc-Me** (see procedure)

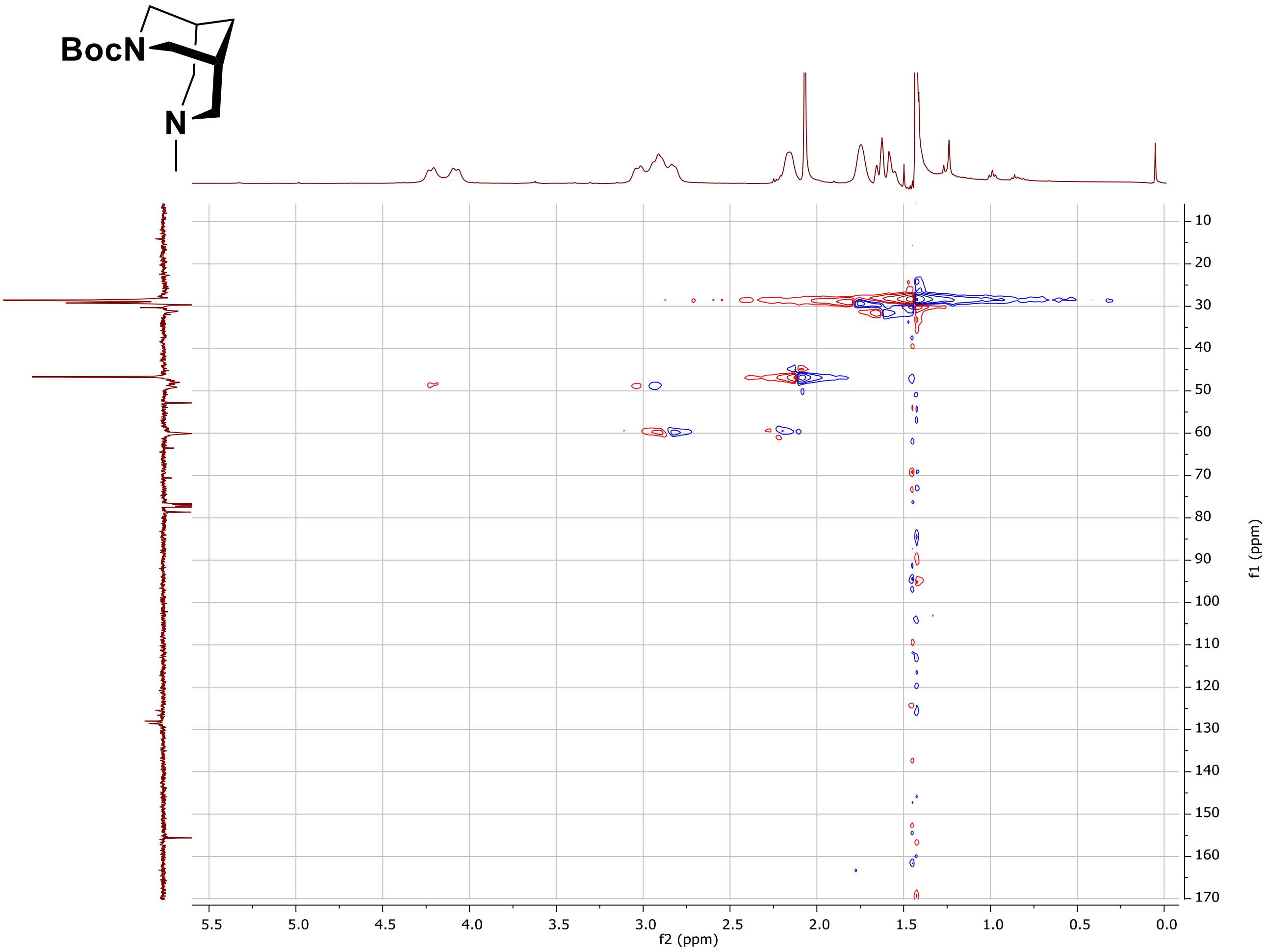

$^1$H NMR (400 MHz,$CDCl_3$) of **4** (see procedure)

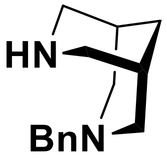


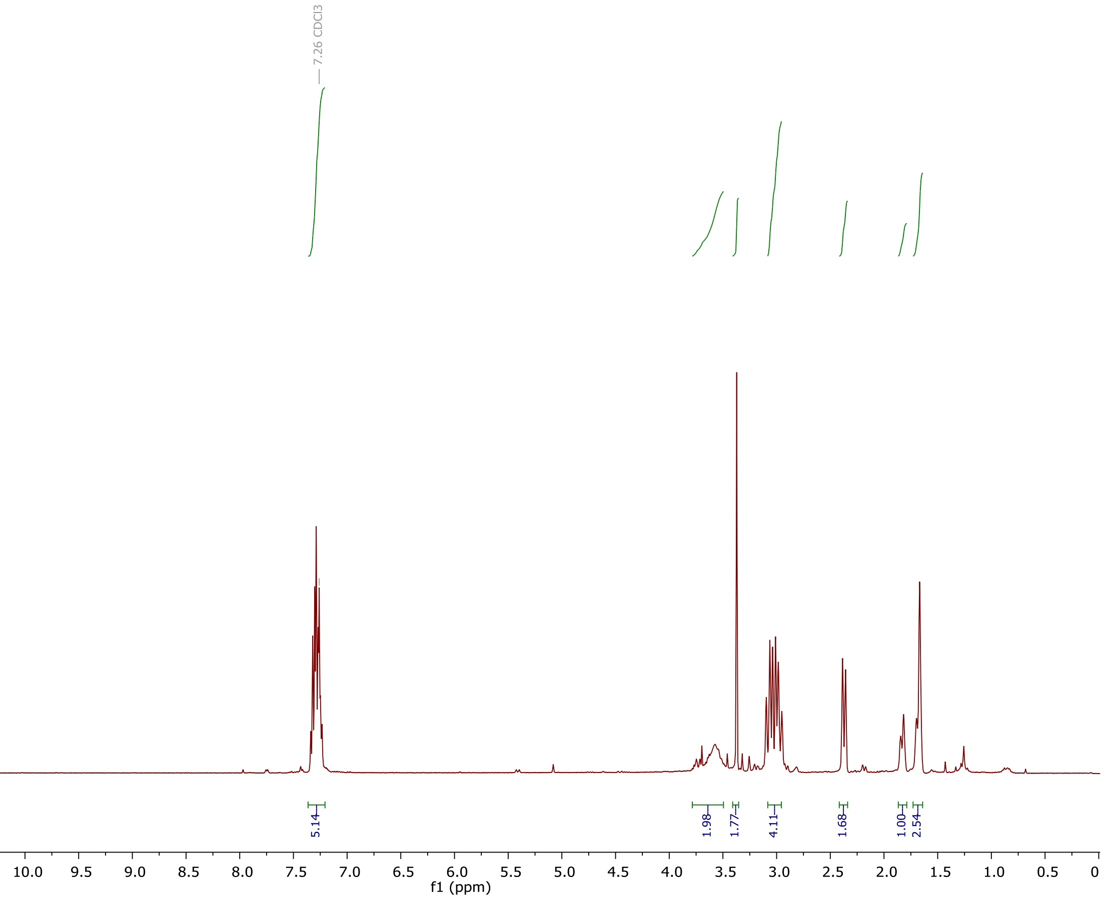

$^{1}$H NMR (400 MHz,$CDCl_3$) of **Bn-NO** (see procedure)

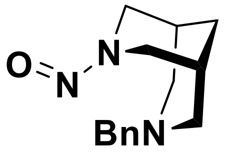


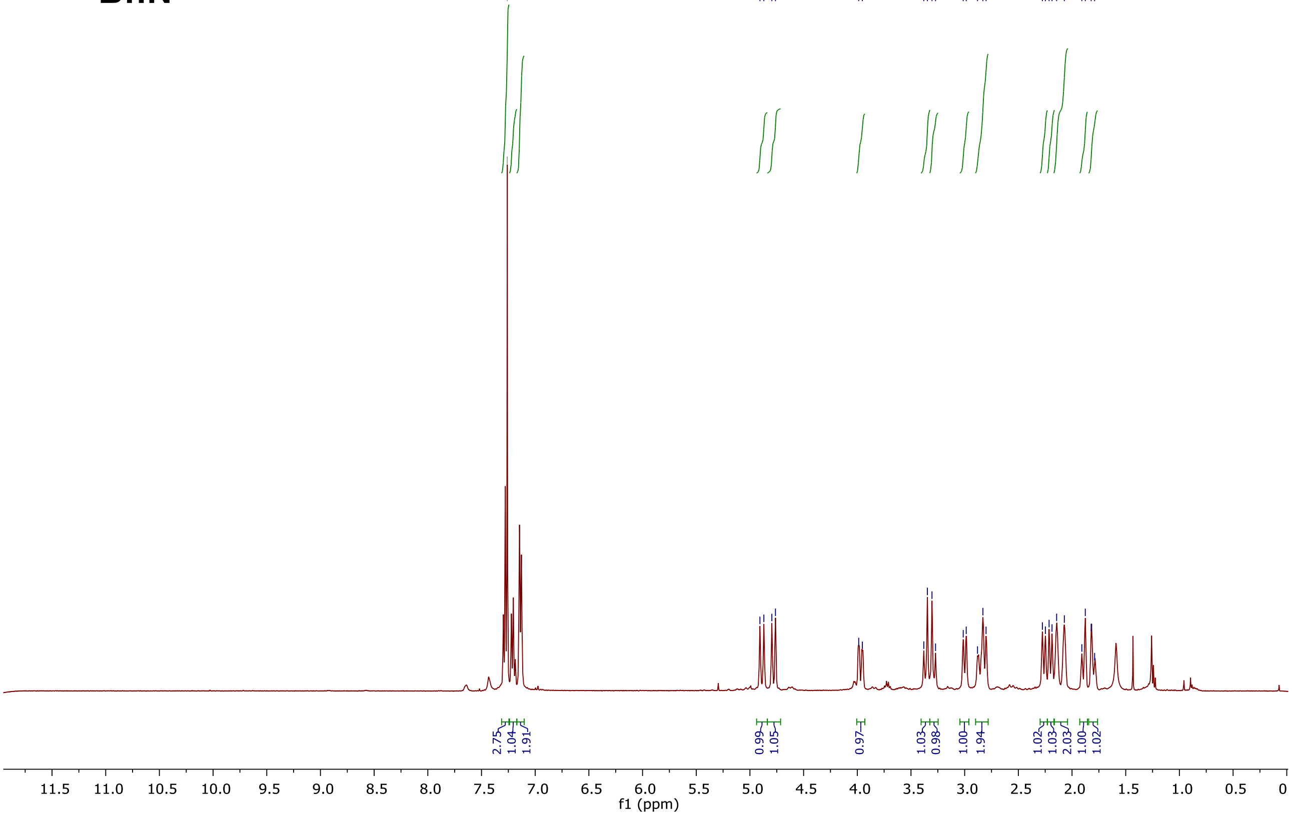

$^1$H NMR (400 MHz, $CD_3OD$ ) of **Bn-NO** (see procedure)

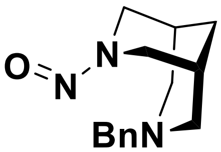


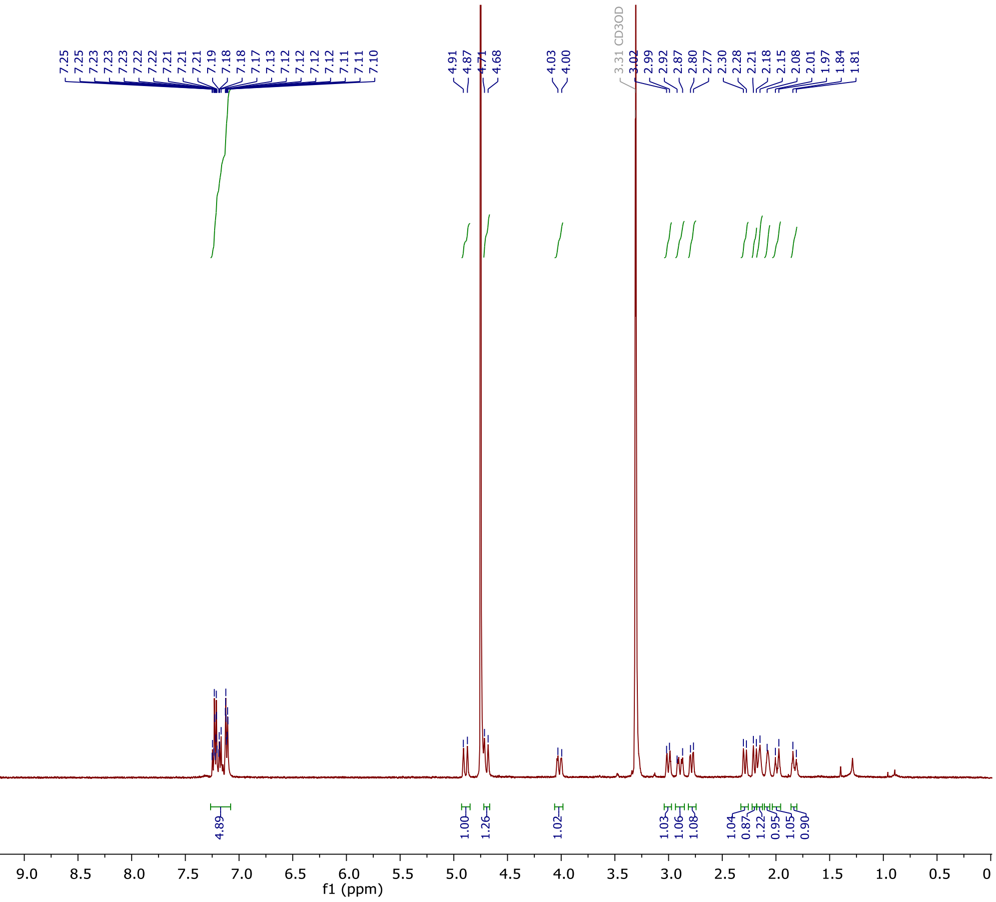

$^{13}$C NMR (400 MHz,$CDCl_3$) of **Bn-NO** (see procedure)

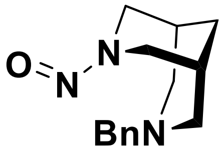


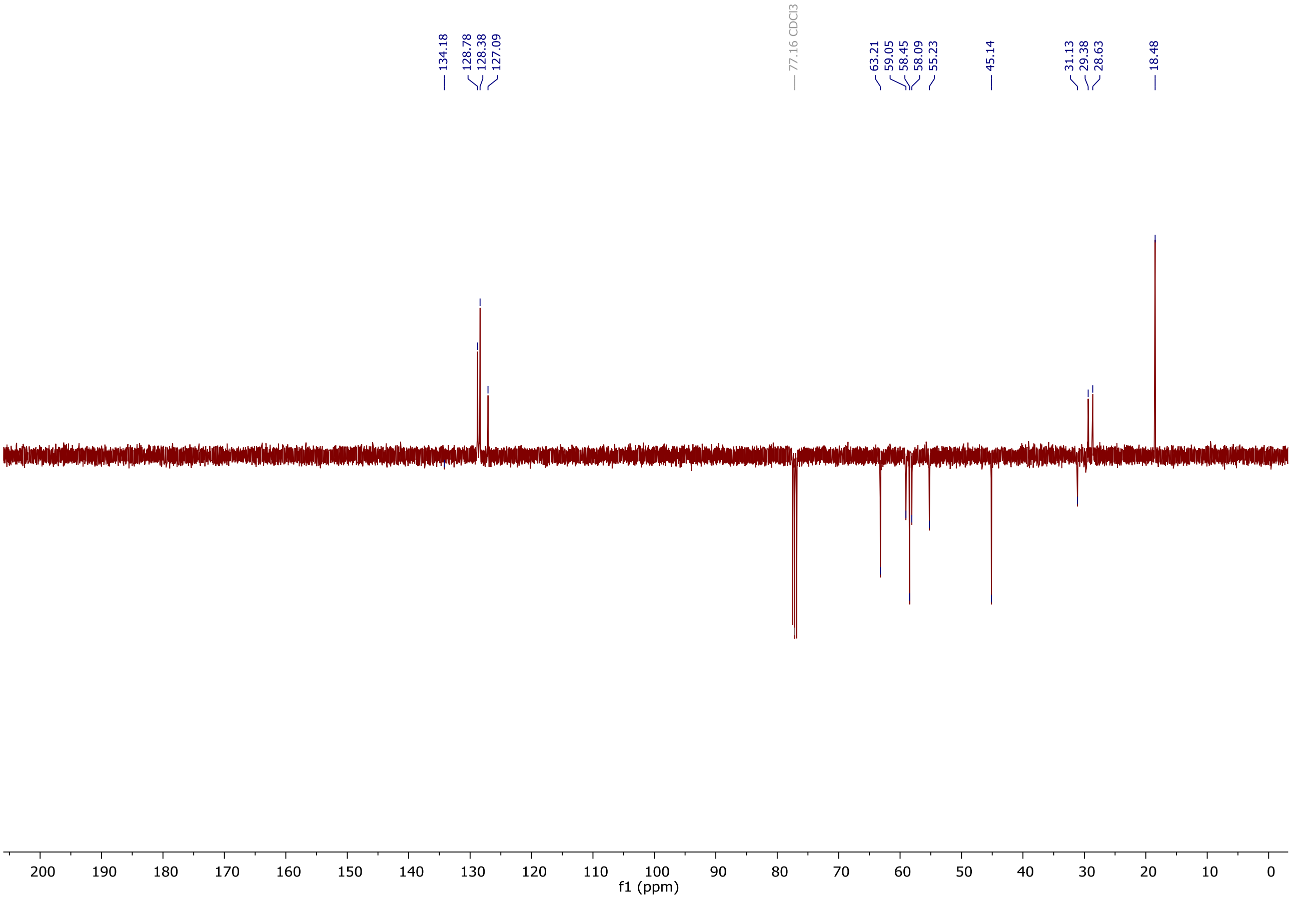

COSY of **Bn-NO** (see procedure)

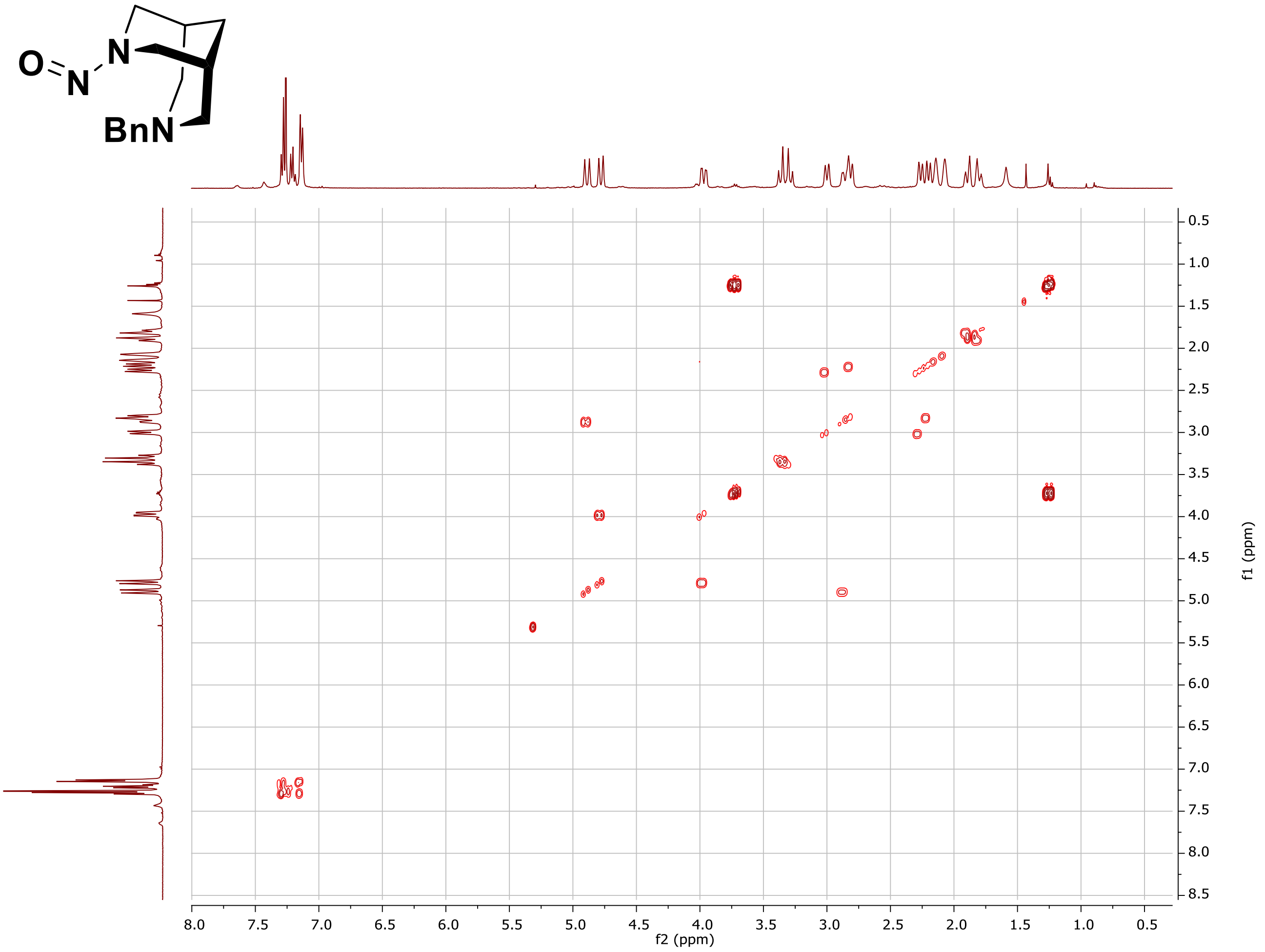

HSQC of **Bn-NO** (see procedure)

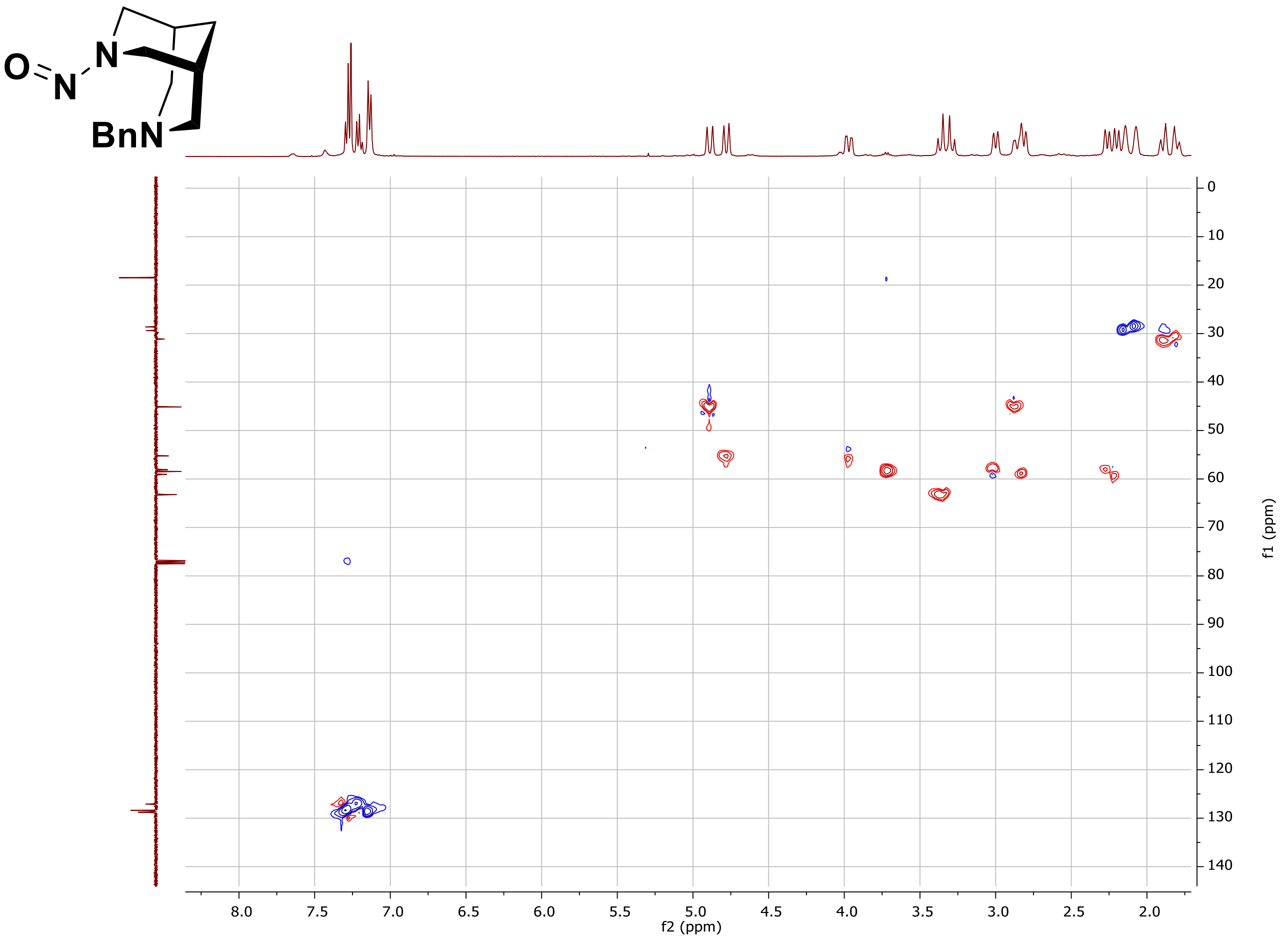

HMBC of **Bn-NO** (see procedure)

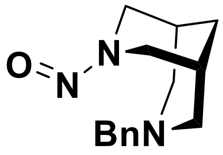


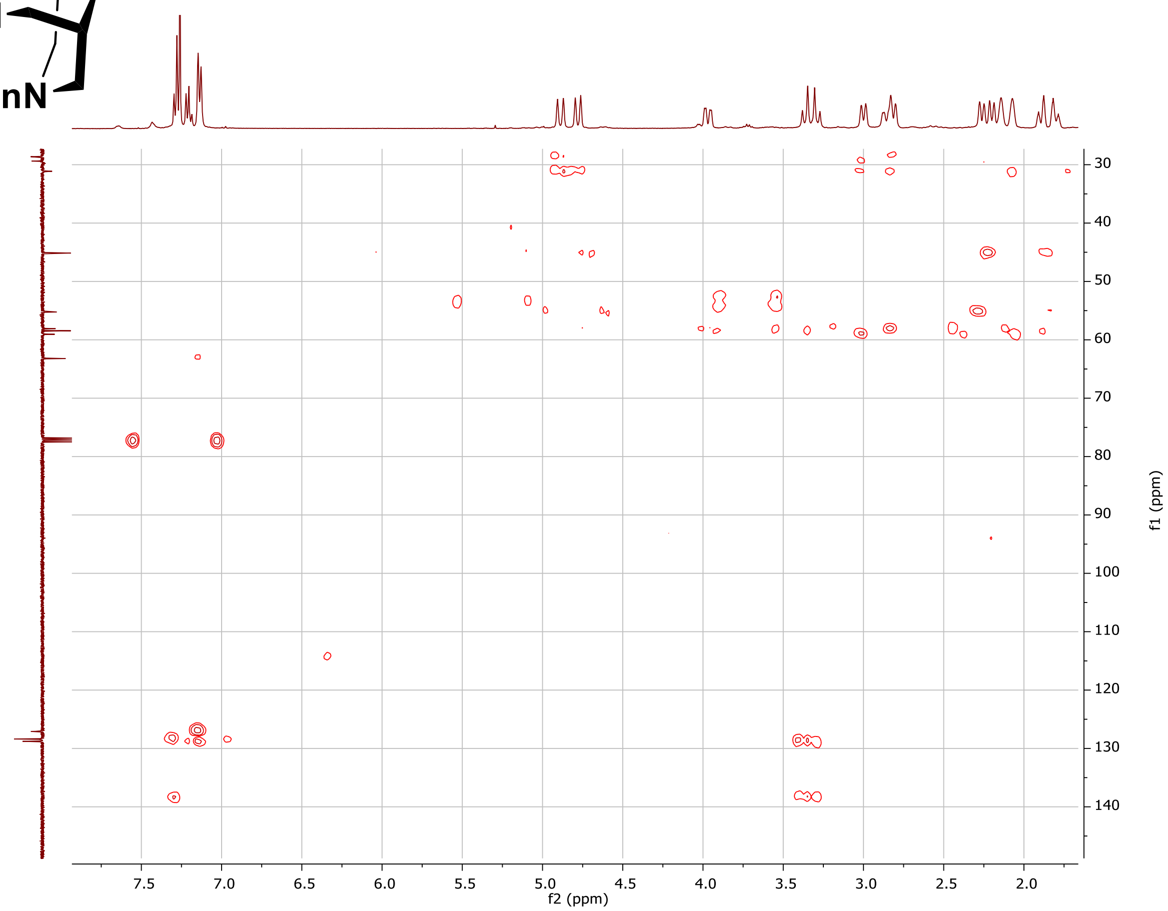

$^{1}$H NMR (400 MHz,$CDCl_3$) of **5** (see procedure)

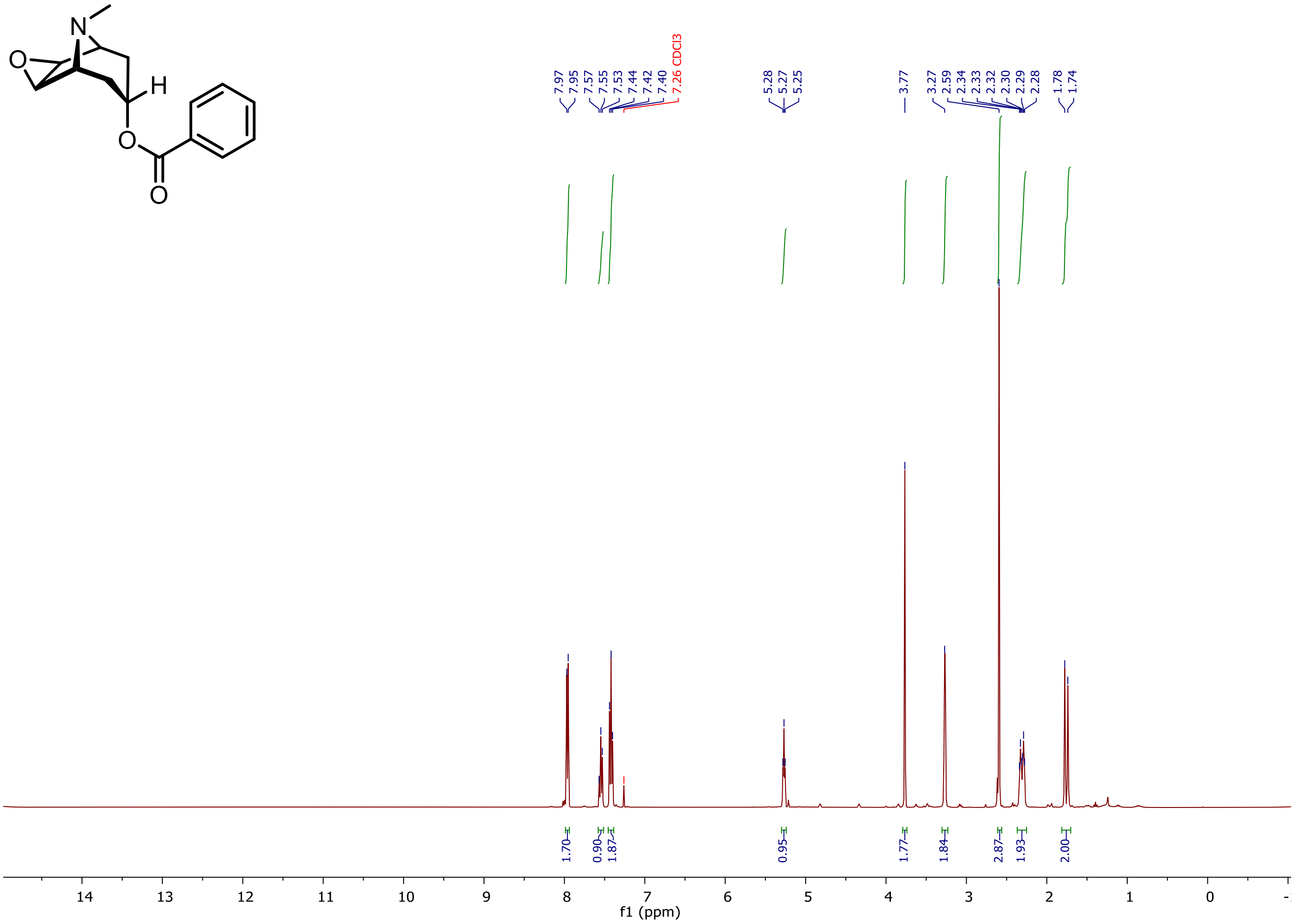

$^{13}$C NMR (400 MHz,$CDCl_3$) of **5** (see procedure)

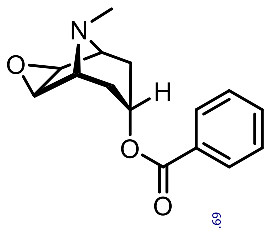


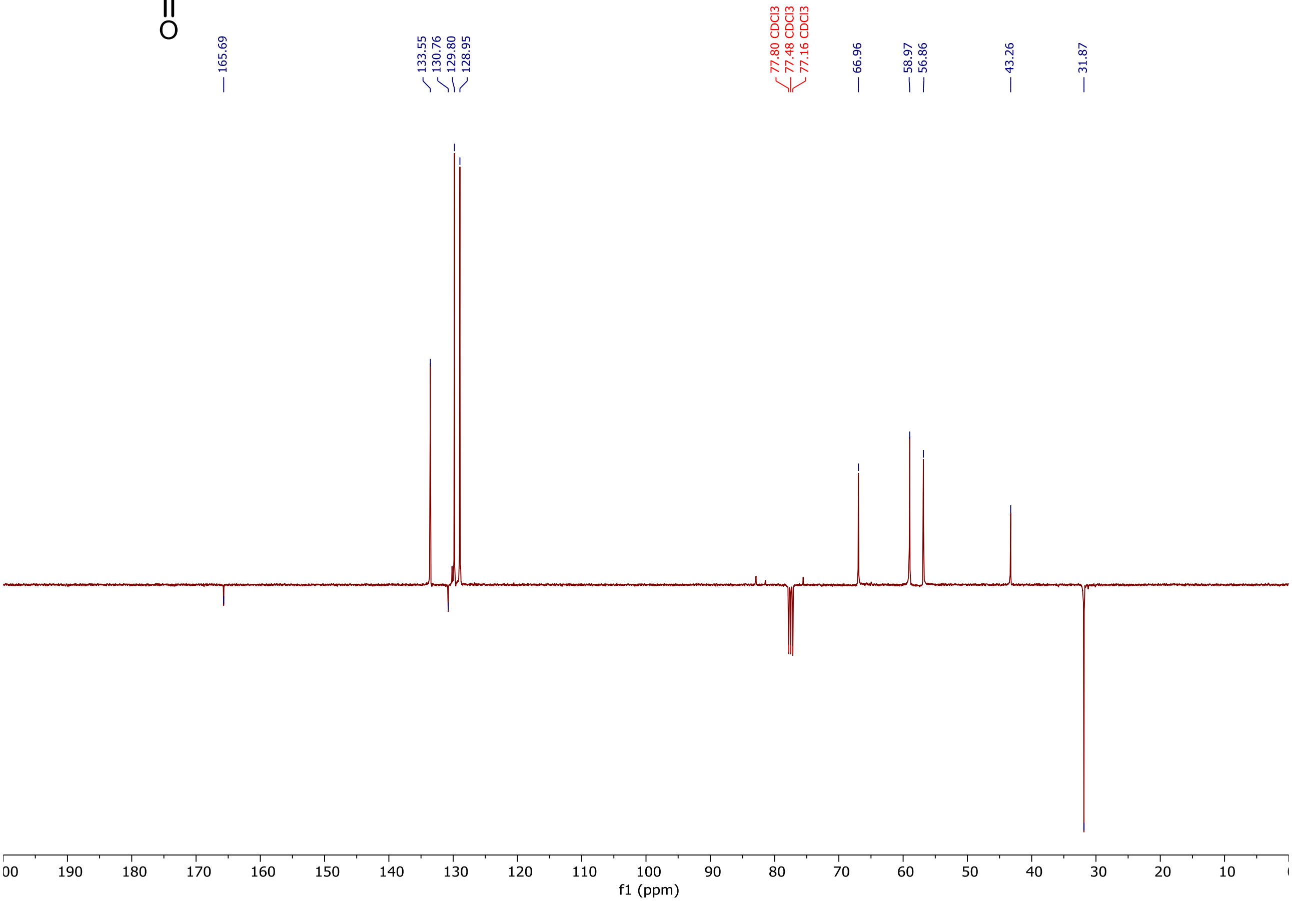

COSY of **5** (see procedure)

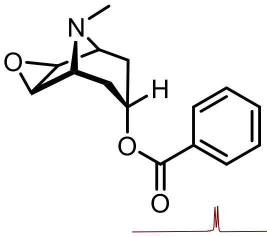


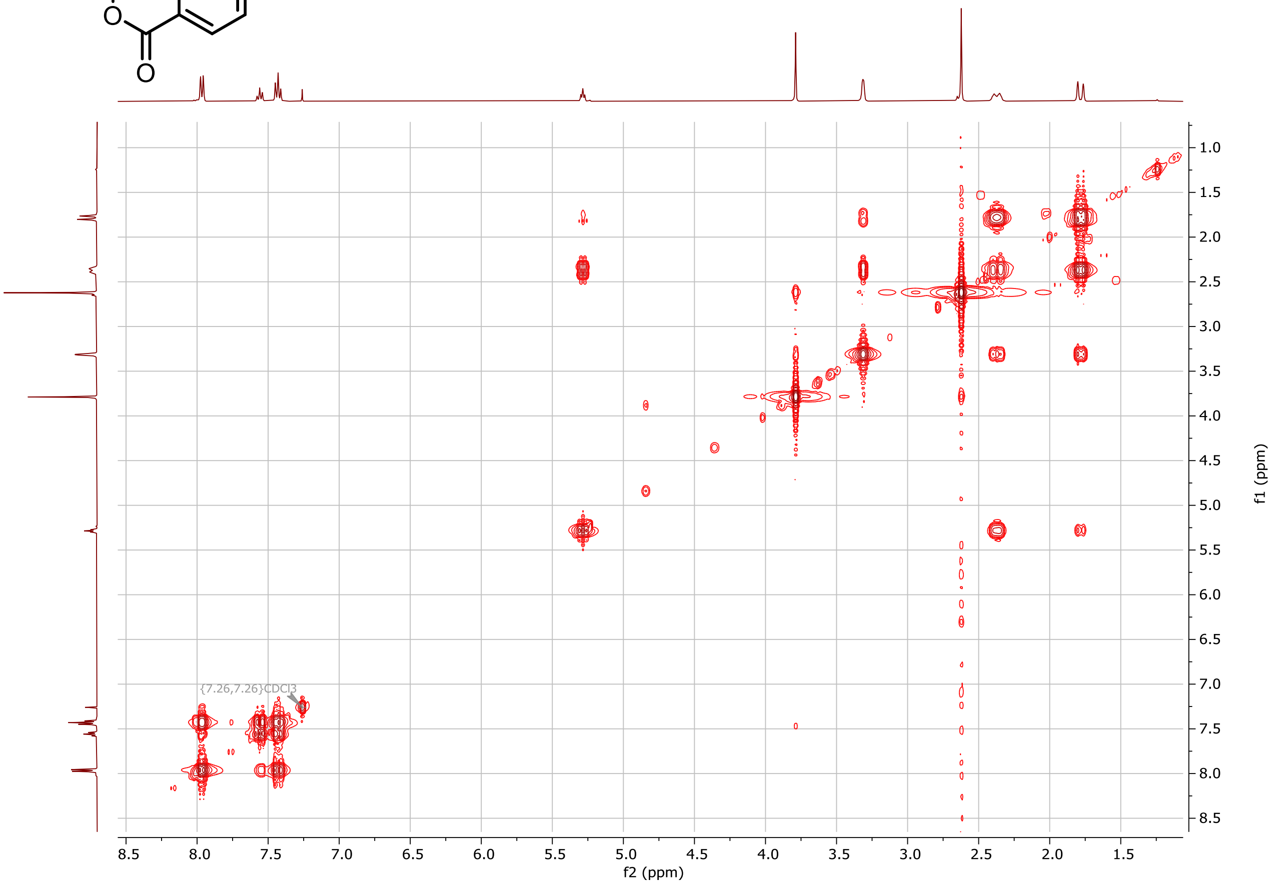

HSQC of **5** (see procedure)

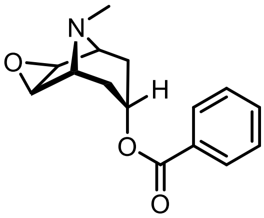


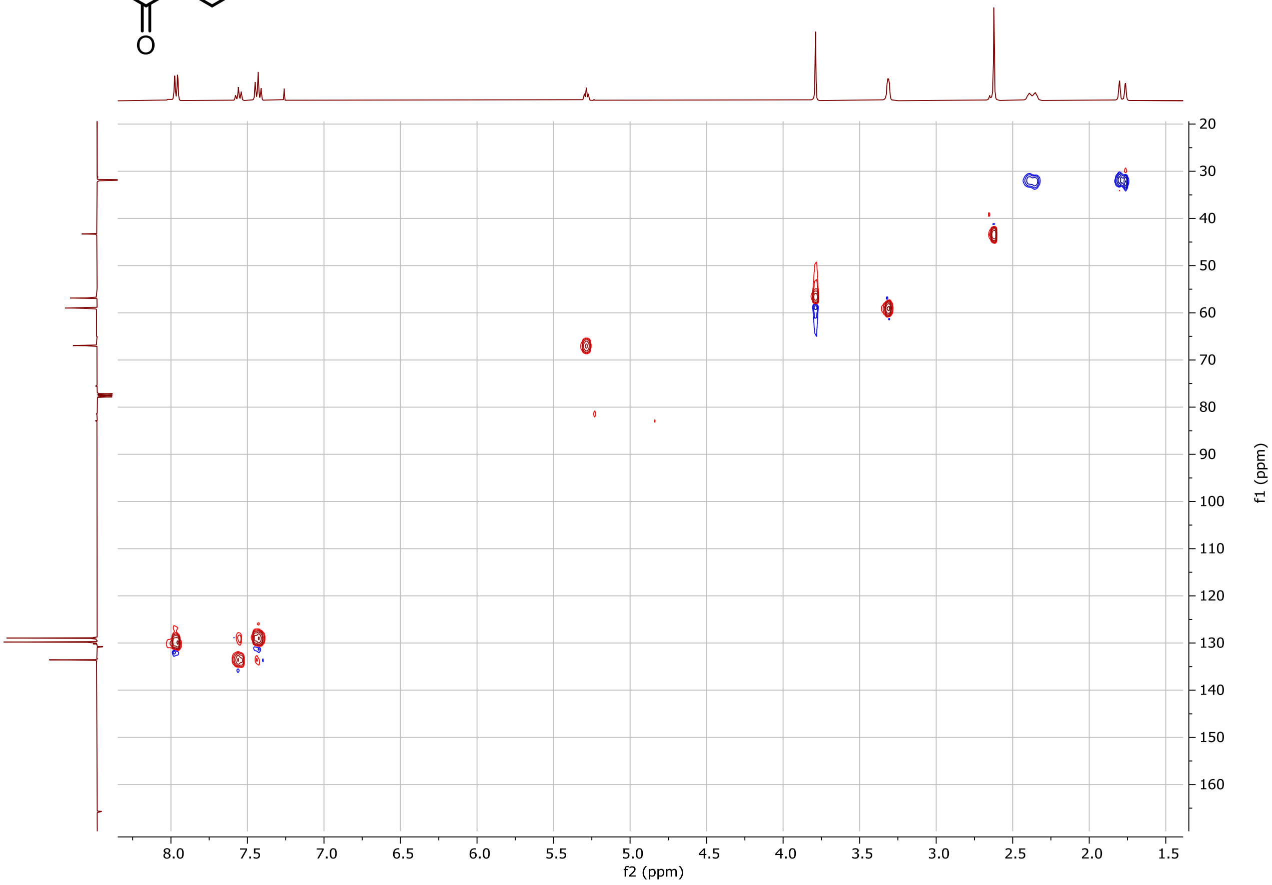

HMBC of **5** (see procedure)

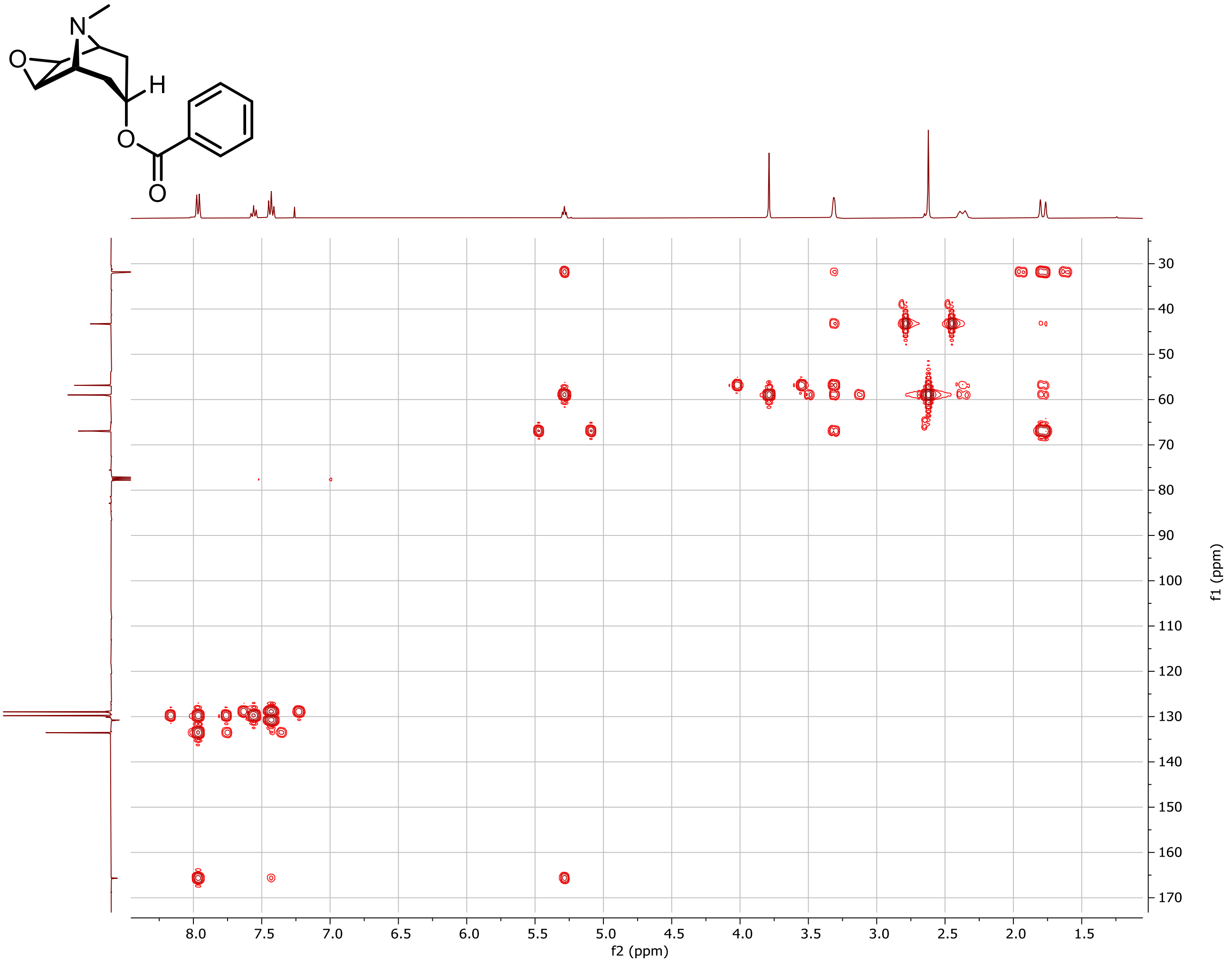

$^{1}$H NMR (400 MHz,$CDCl_3$) of **6** (see procedure)

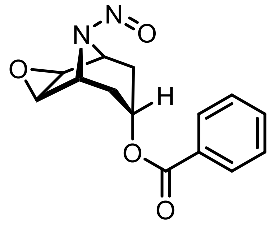


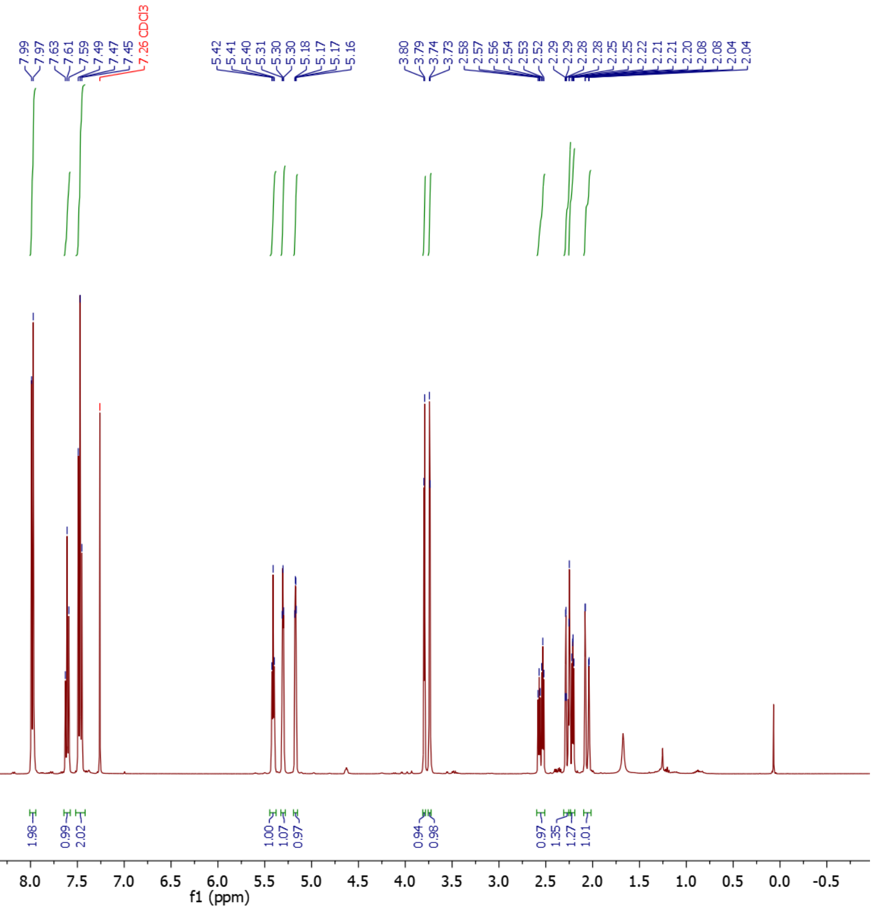

$^{13}$C NMR (400 MHz,$CDCl_3$) of **6** (see procedure)

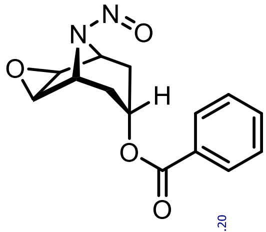


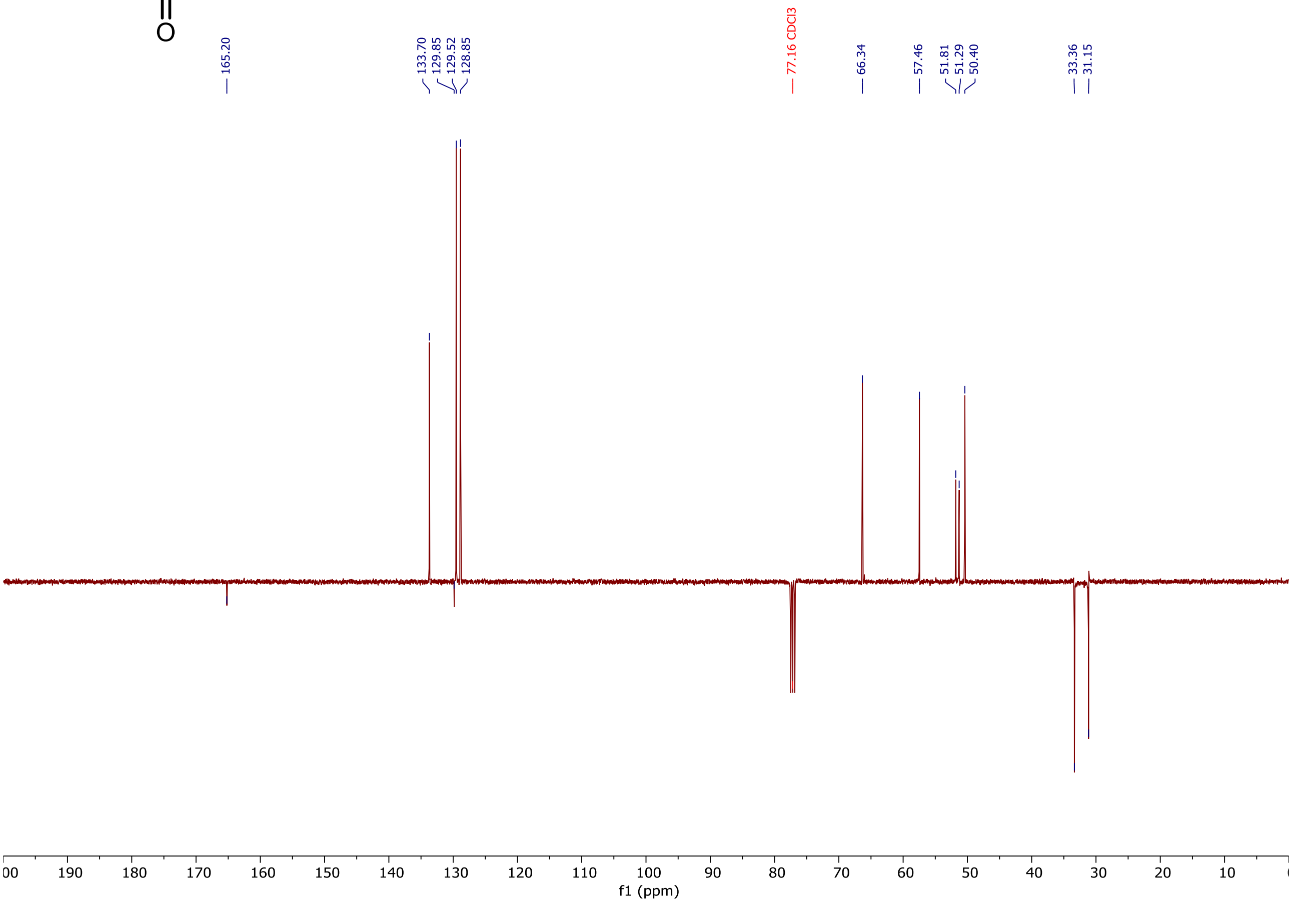

COSY of **6** (see procedure)

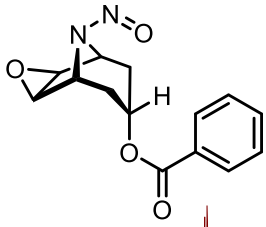

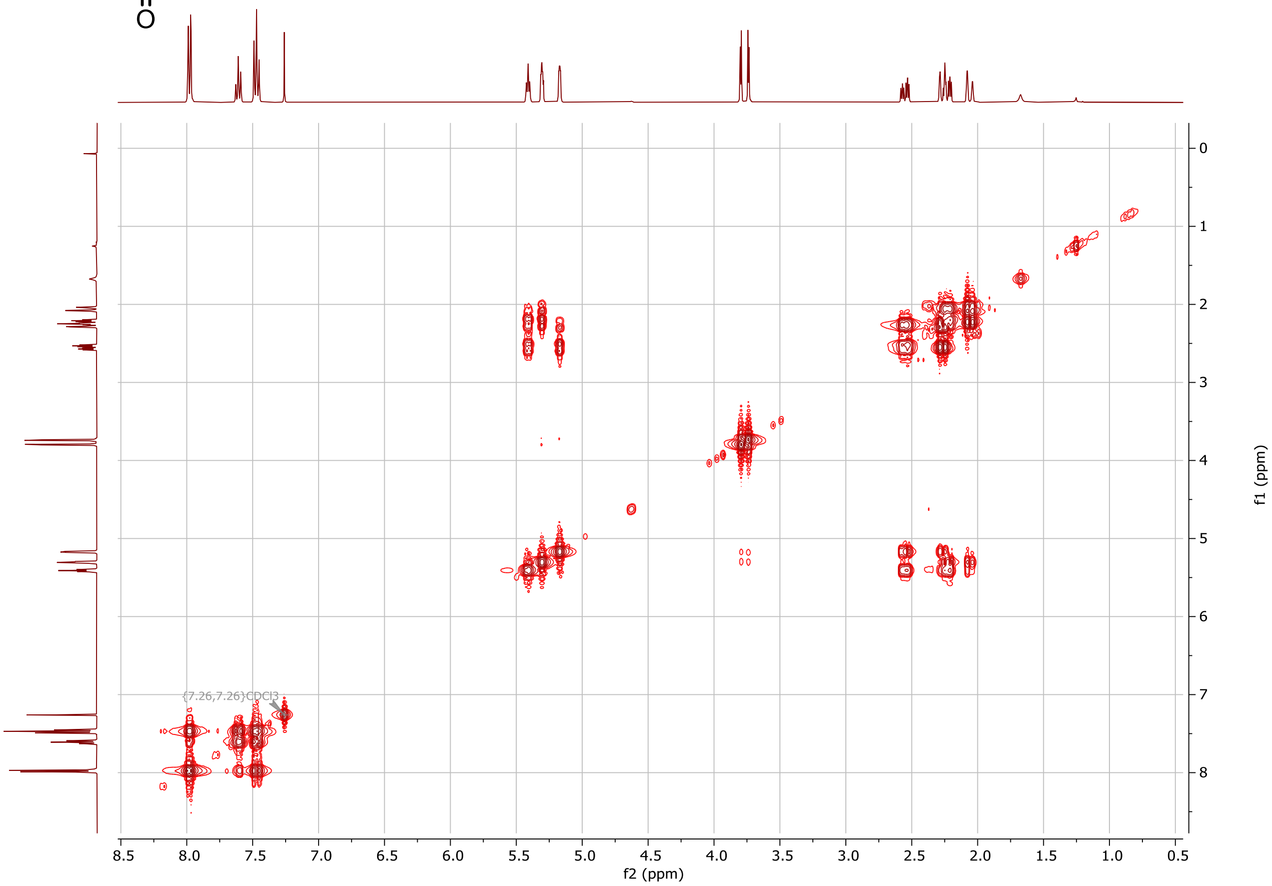

HSQC of **6** (see procedure)

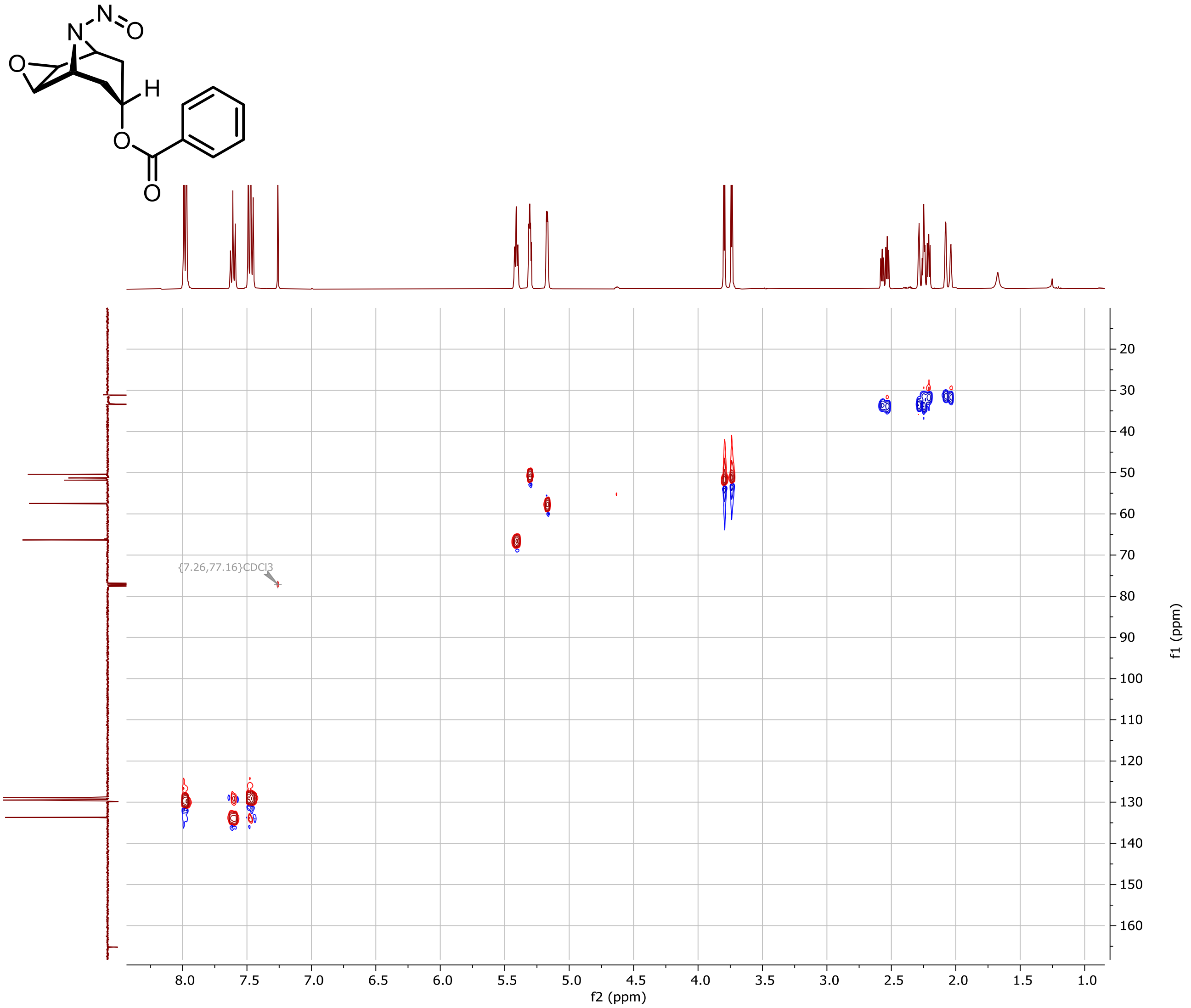

HMBC of **6** (see procedure)

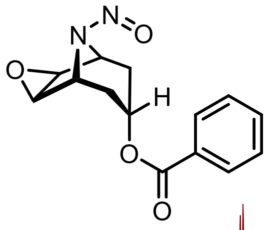

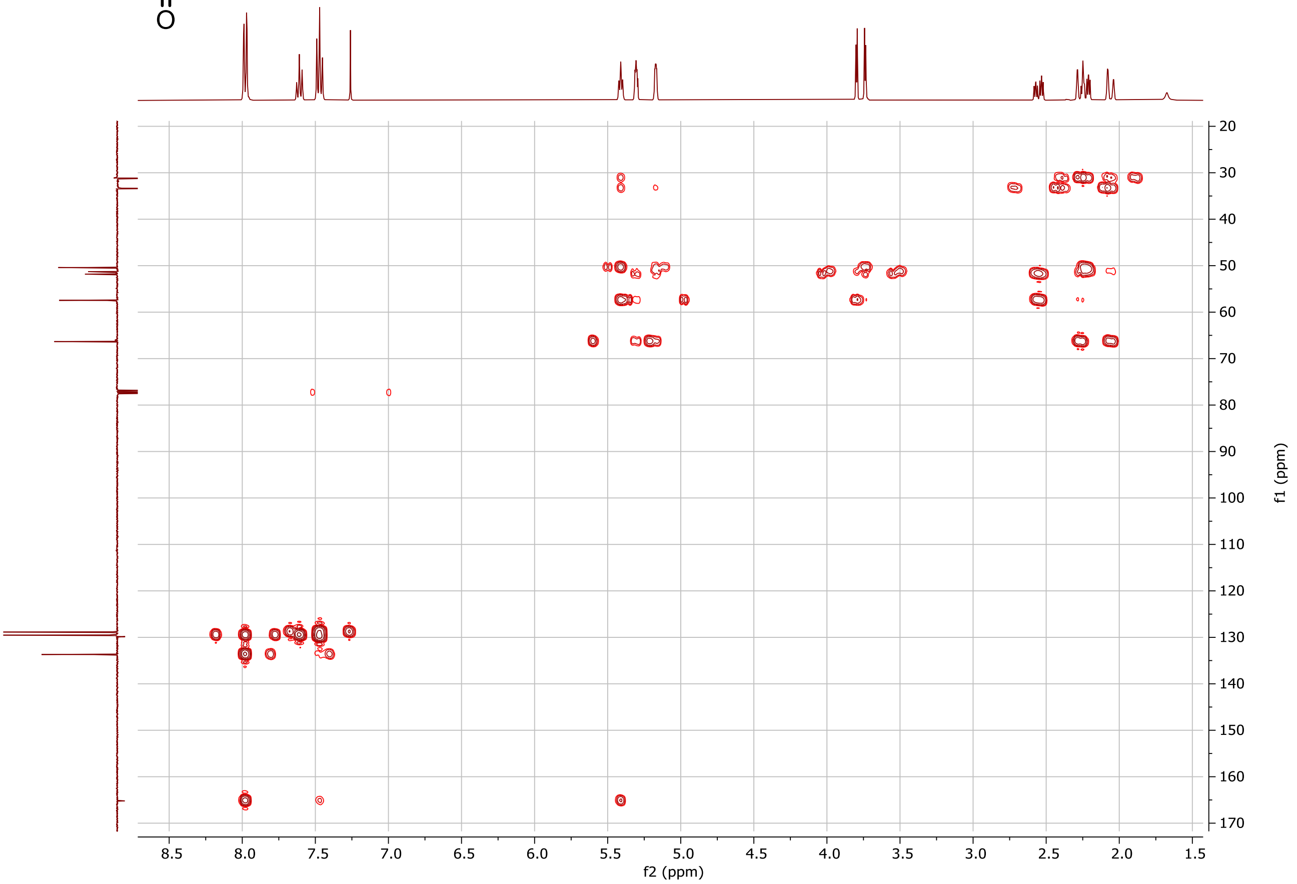

$^{1}$H NMR (400 MHz,CDCl$_3$) of **7** (see procedure)

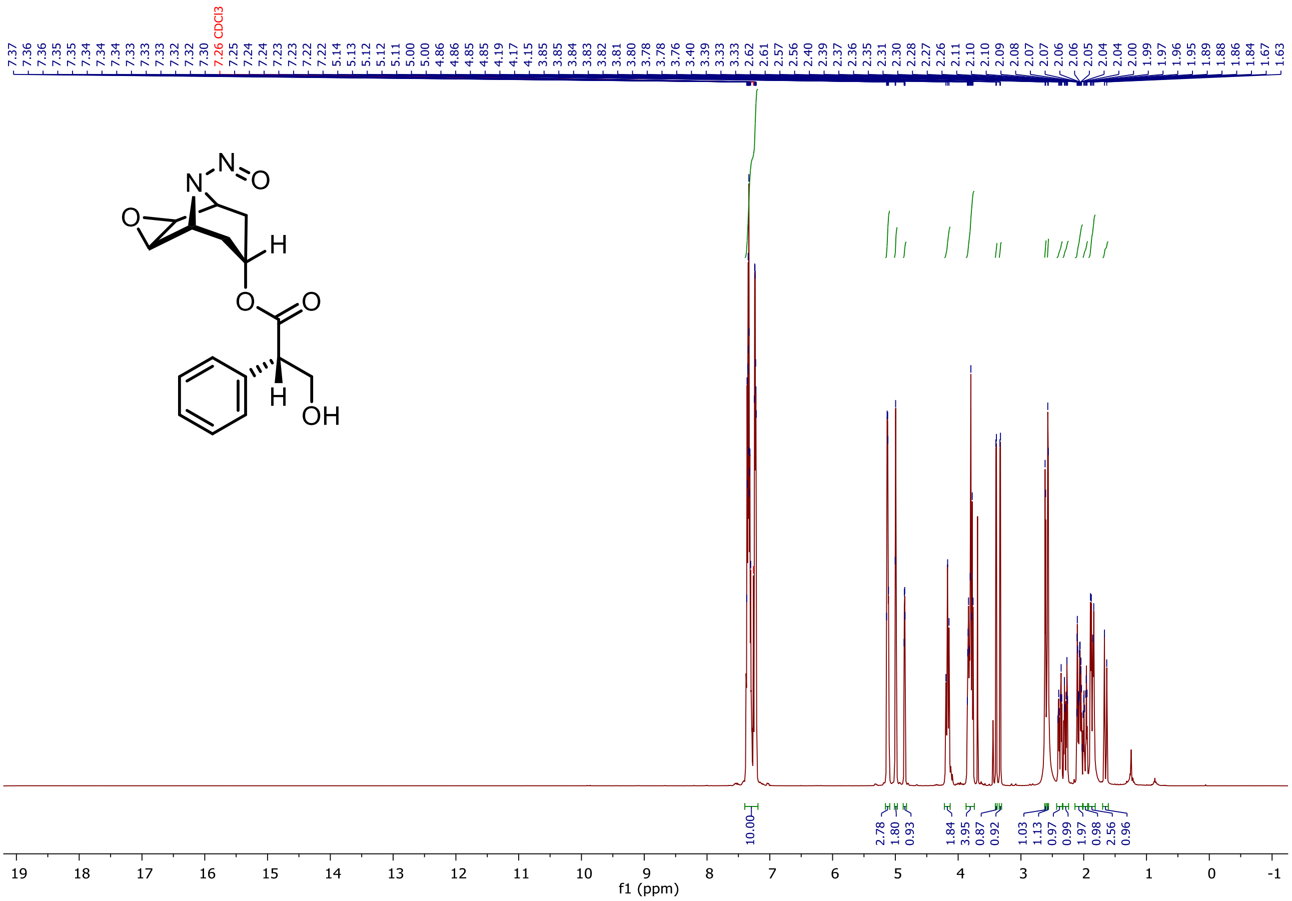

$^{13}C$ NMR (400 MHz,$CDCl_3$) of **7** (see procedure)

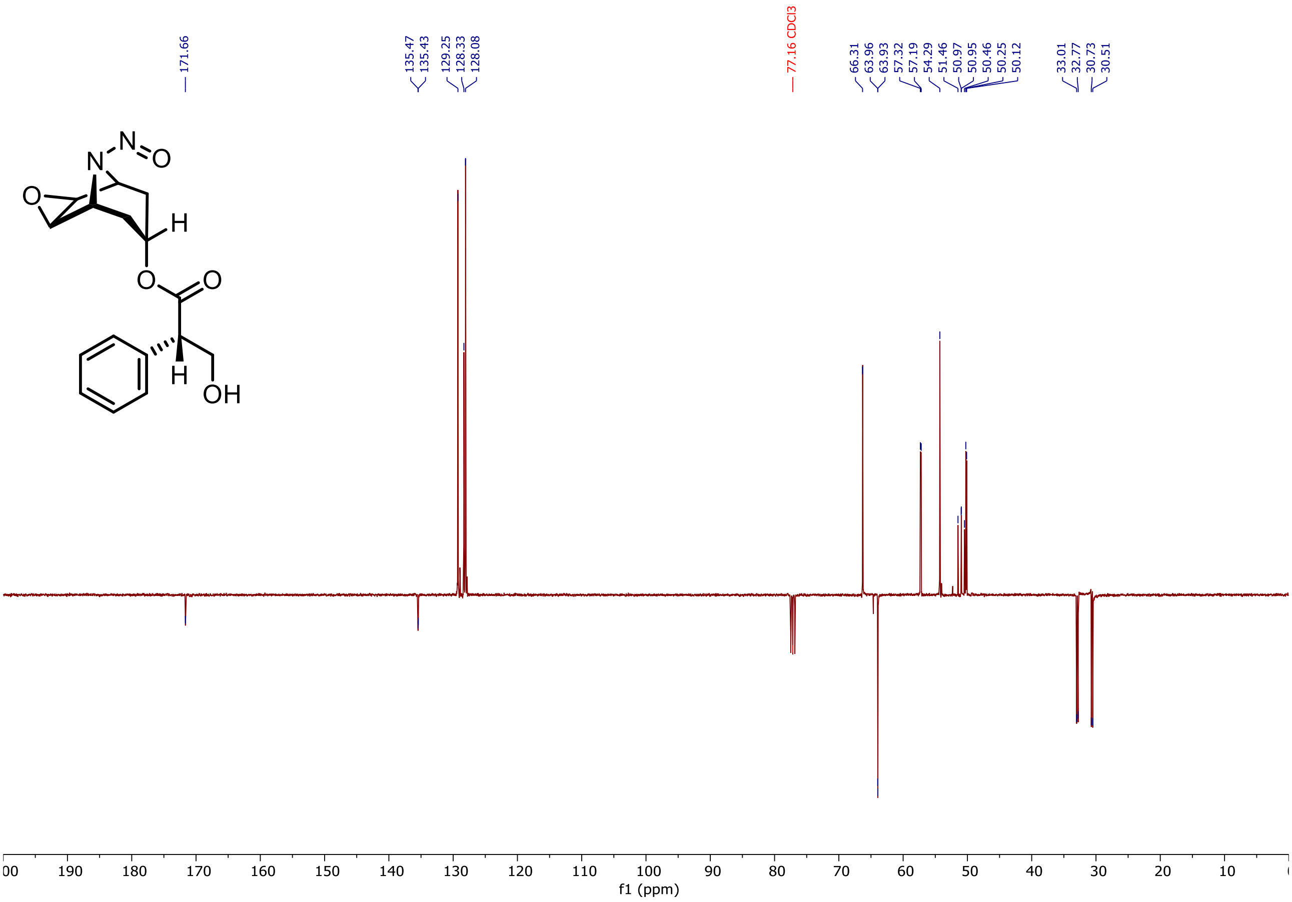

COSY of **7** (see procedure)

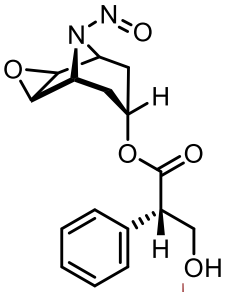

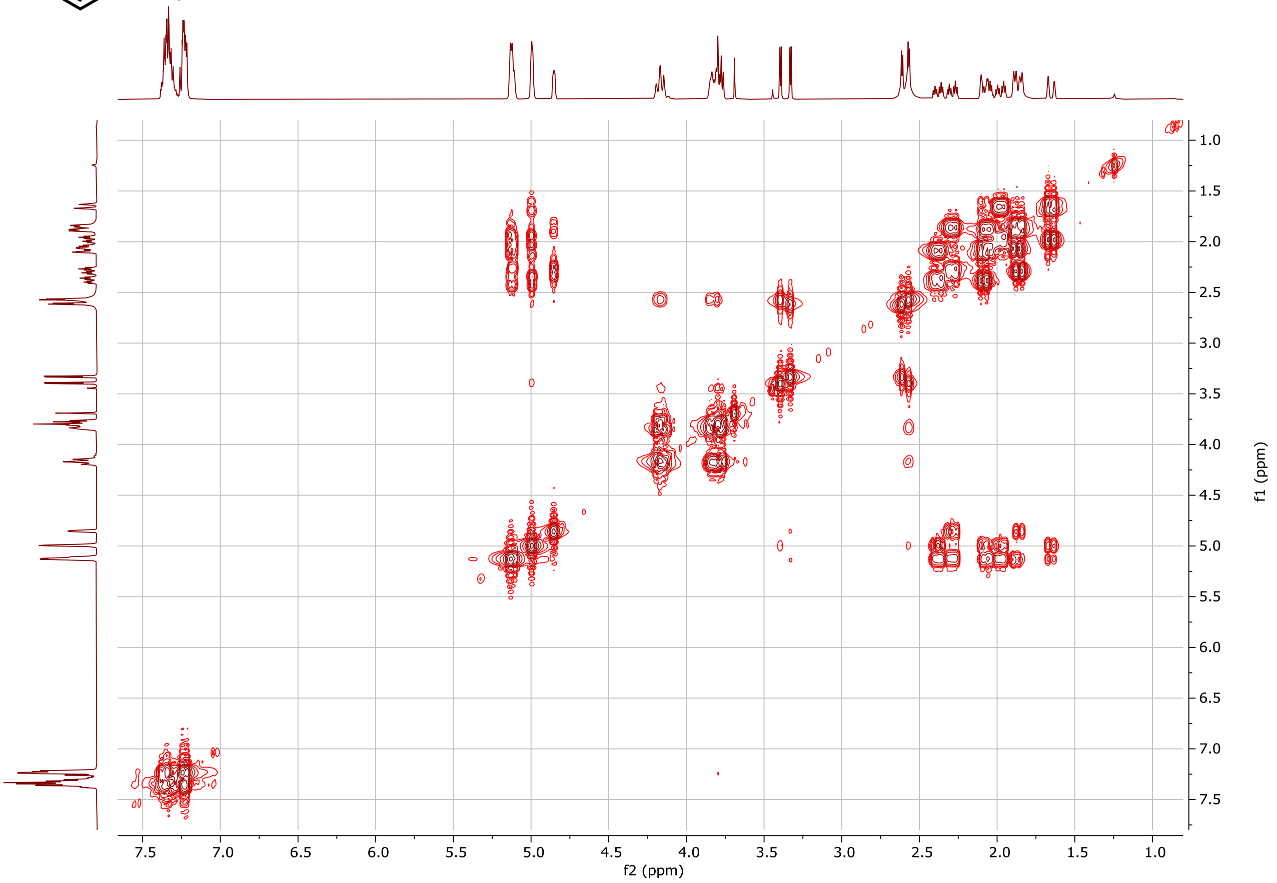

HSQC of **7** (see procedure)

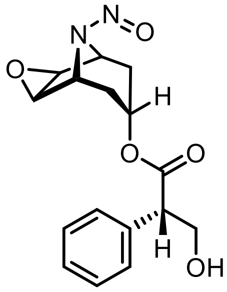


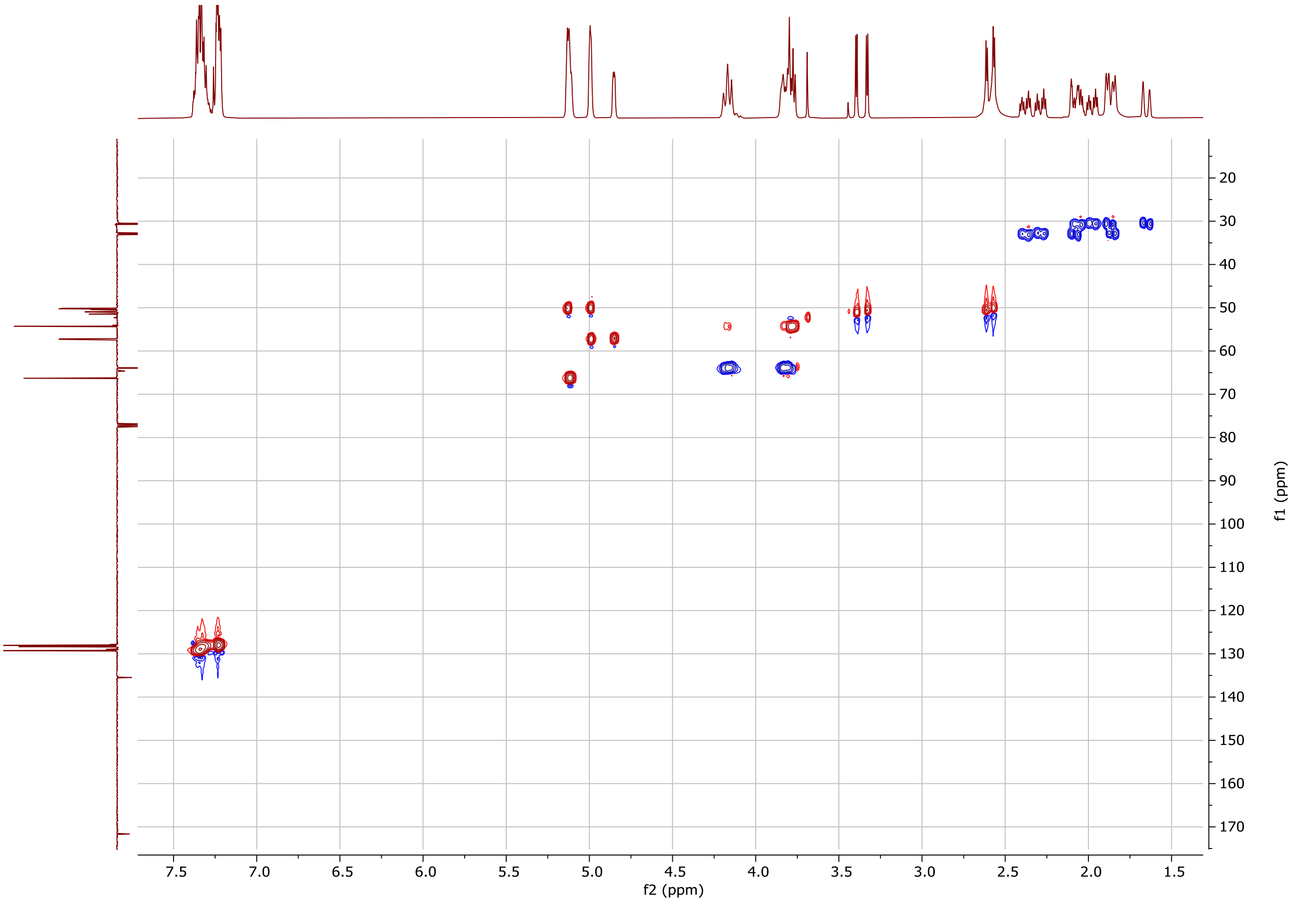

HMBC of **7** (see procedure)

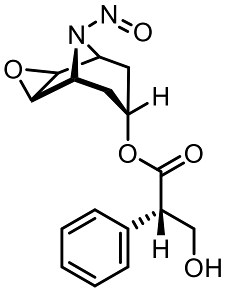


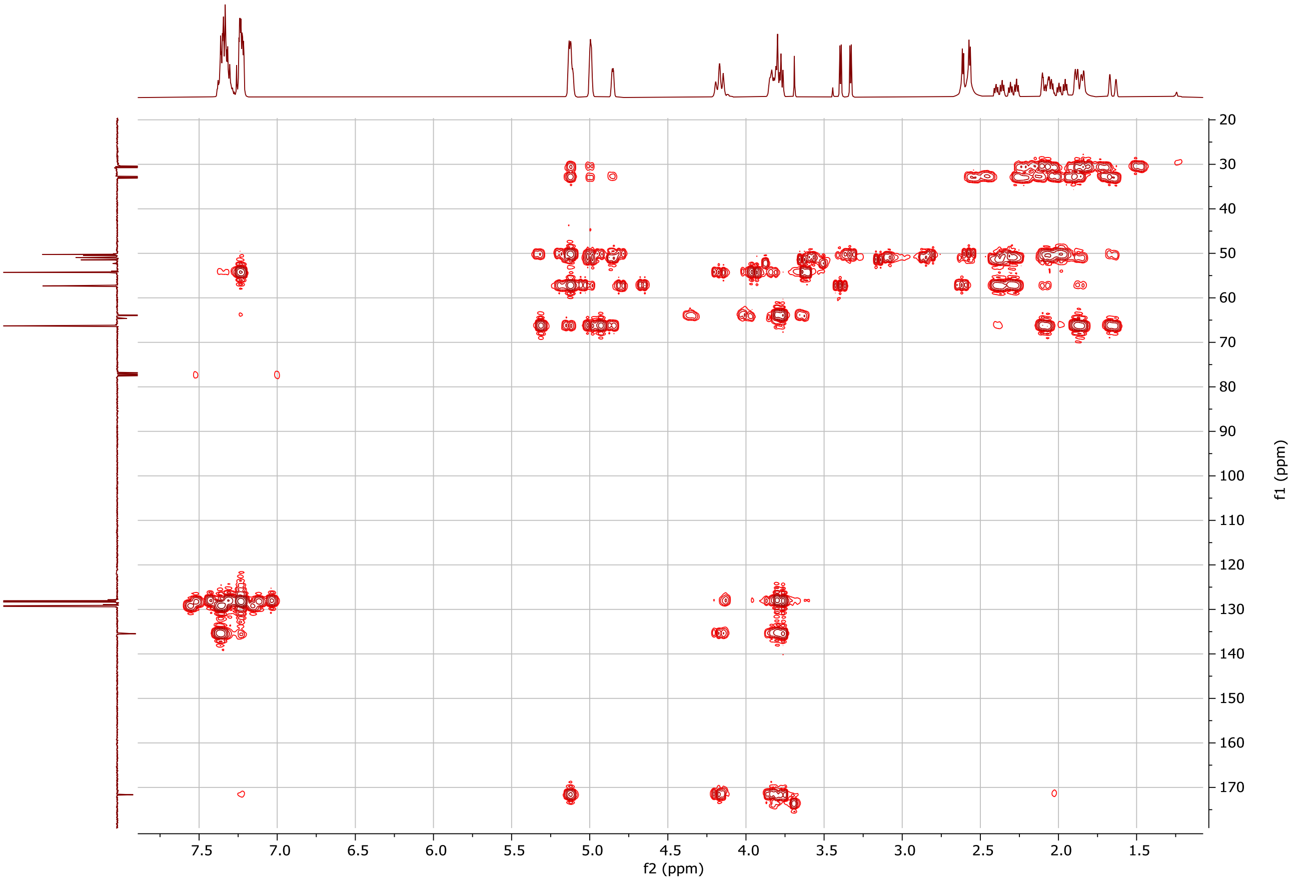

## 4. DFT CALCULATIONS

Calculations were performed using the Gaussian09[5] suite of programmes. Geometries were optimized with the DFT method using M06-2X functional as implemented in Gaussian, using 6-311G+(d,p) as basis set for all the atoms. All calculations were carried out using Polarizable Continuum Model (PCM) to include solvation (solvent=chloroform). All geometry optimizations were full, with no restrictions. All stationary points located in the potential energy hypersurface were characterized as minima (no imaginary frequencies) or as transition states (one and only one imaginary frequency) by vibrational analysis. The analysis also provided zero-point vibrational energy corrections and thermal corrections to various thermodynamic properties. **TS1$_{\mathbf{Ac\text{-}H}}$** (the prototype of transition states discussed in this work) was further investigated by IRC calculation and scan analysis. Full Cartesian coordinates for the optimised geometries are reported at the end of this section.

*Conformational analysis of **Ac-H***

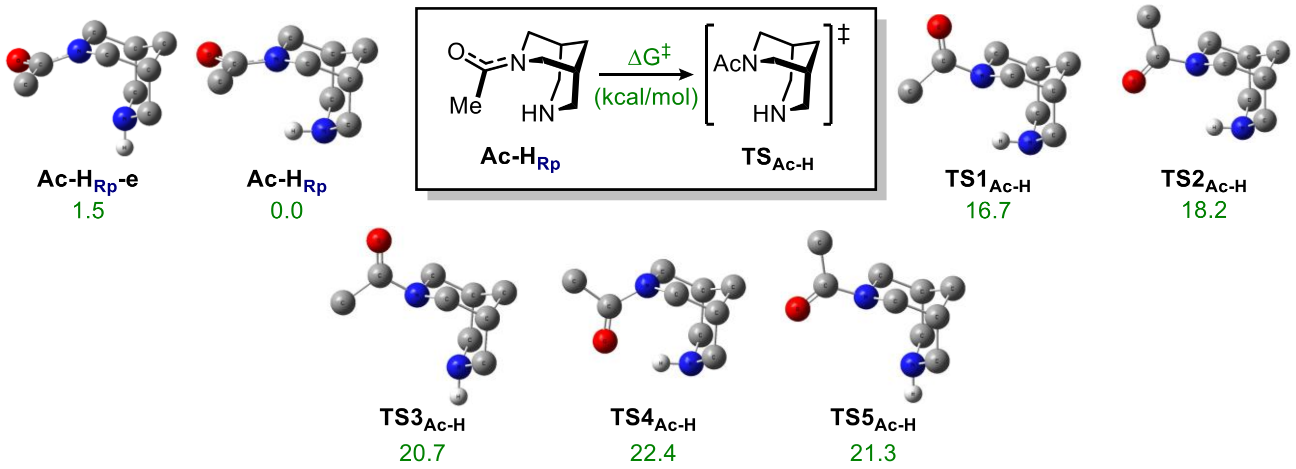


The other three possible transition states having Ac in axial position were not stable and relaxed to one of the previous calculated TSs.

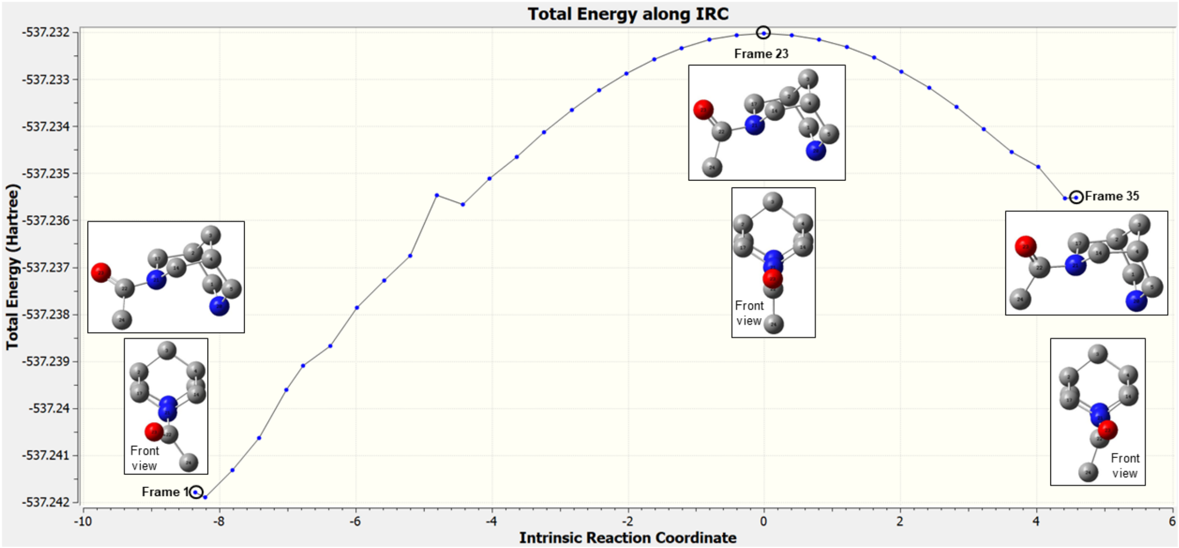


**Figure S3**. IRC analysis of **TS1**$_{\mathbf{Ac\text{-}H}}$.

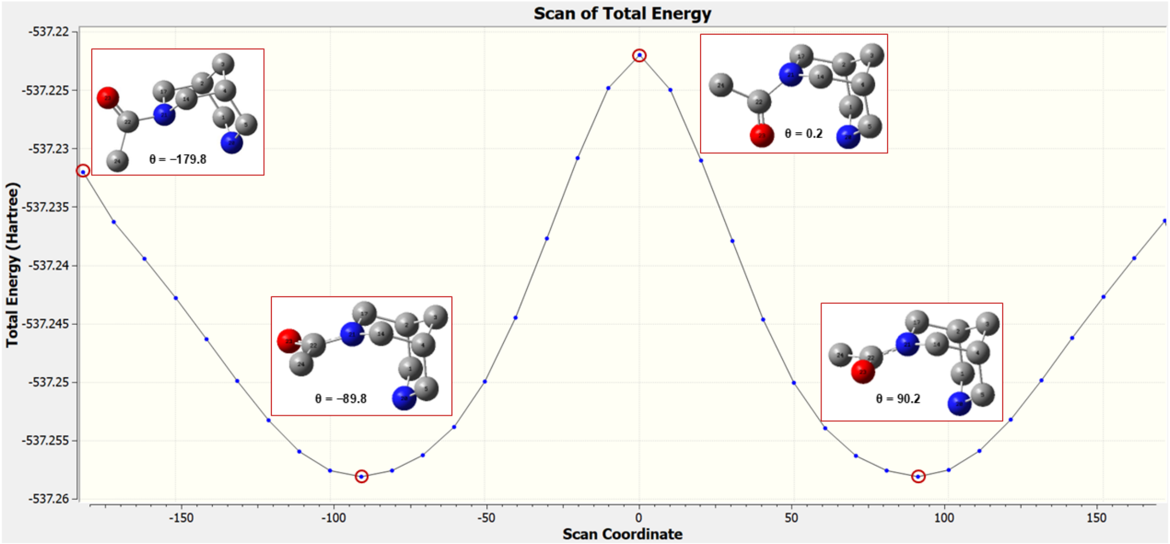


**Figure S4**. Scan analysis starting from **TS1**$_{\mathbf{Ac\text{-}H}}$ (scanning θ (20-21-22-23) every 10° and collecting 36 points).

*Conformational analysis of* ***Ac-Me***

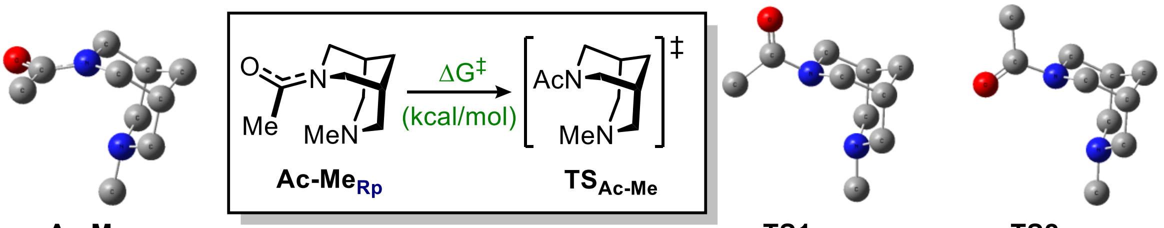


**Ac-Me**$_{\mathbf{Rp}}$ 0.0 **TS1**$_{\mathbf{Ac\text{-}Me}}$ 19.3 **TS2**$_{\mathbf{Ac\text{-}Me}}$ 21.2

The other possible transition states were not stable or much higher in energy.

*Conformational analysis of* ***Boc-R'***

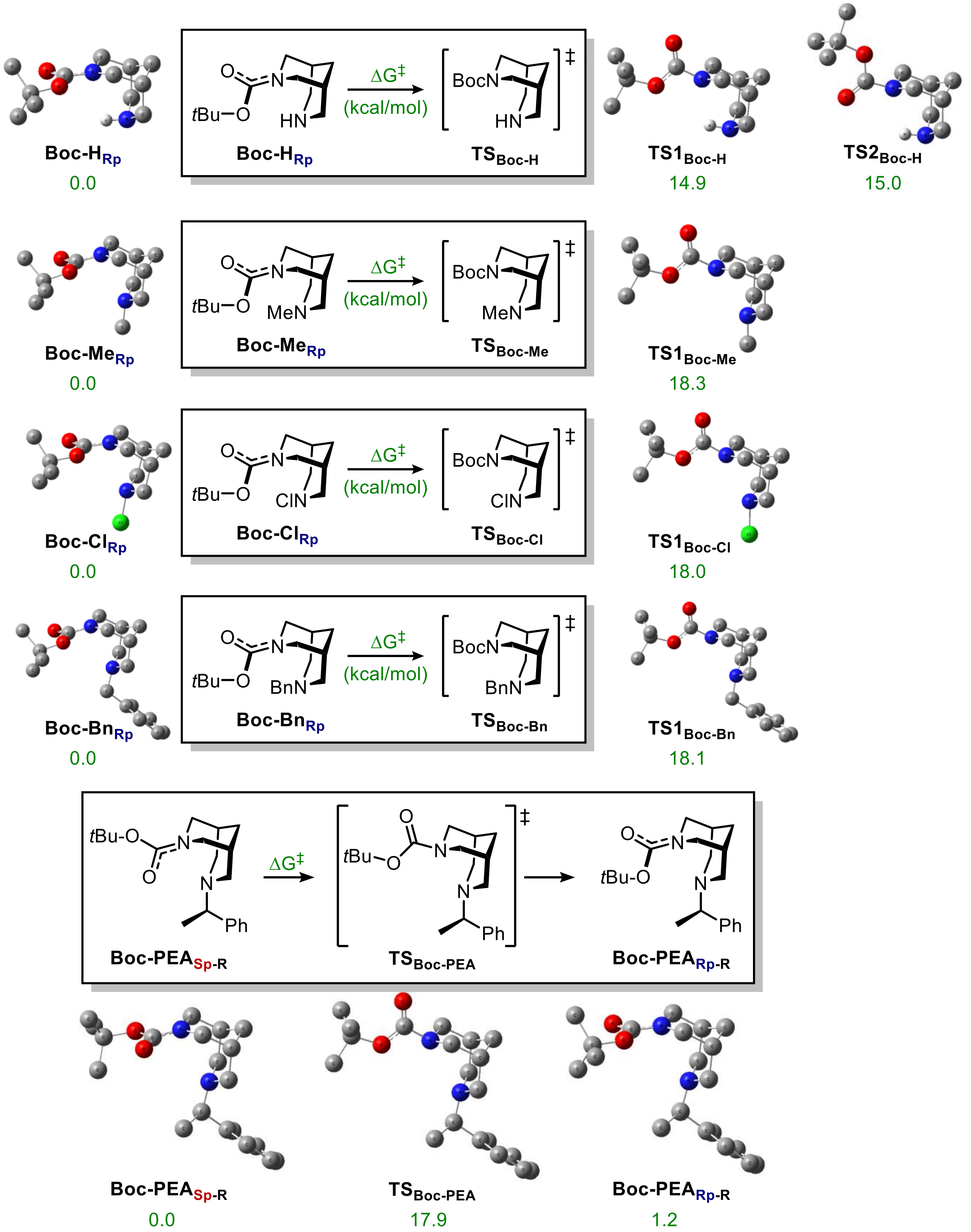

**Ac-H$_{Rp}$-e**

C 1.61020000 0.60982400 1.36359200

C 1.26677700 -0.87191700 1.15070600

C 1.77101600 -1.30641900 -0.22837800

C 1.03958700 -0.44802000 -1.26420400

C 1.42390000 1.01925700 -1.04134700

H 1.76695000 -1.44791400 1.93483500

H 2.70991200 0.69035100 1.40825700

H 1.21041200 0.95491300 2.32047000

H 2.85249200 -1.15785100 -0.30841400

H 1.56735100 -2.36930400 -0.39394900

H 1.34524200 -0.73159600 -2.27570500

H 2.50869300 1.11202300 -1.22287900

H 0.91394800 1.66076200 -1.76635500

C -0.47289600 -0.70923200 -1.15432500

H -1.01807400 -0.09375900 -1.86564100

H -0.65915400 -1.75699900 -1.41975900

C -0.23603300 -1.17431300 1.24413800

H -0.38619200 -2.25450400 1.13127600

H -0.64464800 -0.86682600 2.20359400

N 1.04680100 1.45103600 0.30376200

N -0.99046600 -0.48984600 0.19546700

C -1.76870900 0.57256500 0.54553400

O -2.02739100 0.83321600 1.71509200

C -2.33863400 1.42708400 -0.56636900

H -1.53156900 1.96450700 -1.06906300

H -2.87803100 0.83531300 -1.30723200

H -3.01804900 2.14507500 -0.11416100

H 1.32666200 2.41376100 0.44962700

**E = -537.039571**

**Ac-H$_{Rp}$**

C 1.91194200 0.79563100 1.24439900

C 1.39354300 -0.65272000 1.19059300

C 1.81651200 -1.28416400 -0.13755400

C 1.16455200 -0.46209800 -1.25124100

C 1.67444000 0.98870200 -1.17243100

H 1.83340200 -1.20205100 2.02854000
H 3.00822400 0.76398600 1.24849600
H 1.59802600 1.27860800 2.17315200
H 2.90597500 -1.26594200 -0.23982800
H 1.49479800 -2.32961500 -0.18976300
H 1.43740900 -0.86585000 -2.23109700
H 2.74801200 0.98068300 -1.39629900
H 1.19094700 1.60425800 -1.93603600
C -0.35950800 -0.56637000 -1.13537800
H -0.82397000 0.06967300 -1.88671200
H -0.66291700 -1.60278900 -1.33854800
C -0.12686400 -0.75043900 1.34098100
H -0.41305500 -1.80594200 1.44490000
H -0.46649200 -0.22519100 2.23200300
N 1.49182800 1.64229100 0.12518900
N -0.83938900 -0.17642300 0.19366800
C -2.04555200 0.41861700 0.41010600
O -2.49846100 0.57581900 1.53891500
C -2.81315400 0.91282700 -0.79958800
H -2.23788400 1.65429400 -1.35798300
H -3.05698500 0.09249000 -1.47718100
H -3.73221300 1.36889900 -0.44132500
H 0.51331700 1.87828000 0.24264200

**E = -537.041956**

**TS1$_{Ac-H}$**

C 1.90388500 1.01229400 1.05188100
C 1.33672200 -0.40534900 1.25327400
C 1.81695700 -1.29598800 0.10426000
C 1.25520100 -0.69927200 -1.18898800
C 1.82357000 0.72069400 -1.36738300
H 1.70622700 -0.78857900 2.20970700
H 2.99510200 0.95531900 1.14564300
H 1.54712300 1.68014200 1.84093800
H 2.91078500 -1.31764600 0.07035200
H 1.46658800 -2.32431100 0.23970900
H 1.56353100 -1.30145800 -2.04948400

H 2.90677500 0.63423500 -1.51570900
H 1.41125400 1.18516800 -2.26748500
C -0.27414300 -0.74249000 -1.12786200
H -0.70556500 -0.29957300 -2.03174800
H -0.60290700 -1.79164200 -1.07454400
C -0.19294600 -0.44993000 1.30553000
H -0.51676300 -1.47897700 1.52431800
H -0.56675700 0.19845500 2.10504700
N 1.58899900 1.62938300 -0.24059500
N -0.78001700 0.01793200 0.03329400
C -2.21996400 -0.04917100 0.08918000
O -2.81003000 -1.08846900 0.23761300
C -2.89495500 1.28230400 -0.05109700
H -2.53773600 1.94008000 0.74539000
H -2.58539500 1.73564500 -0.99607600
H -3.97714300 1.18175600 -0.00890300
H 0.59808500 1.84491000 -0.23364400

**E = -537.015269**

**$TS2_{Ac-H}$**

C 1.87683900 1.02829500 1.05636700
C 1.38550900 -0.41889100 1.25002200
C 1.90949800 -1.27572000 0.09415300
C 1.31540900 -0.70234400 -1.19547300
C 1.80729800 0.74741700 -1.36575600
H 1.77682200 -0.78837700 2.20310300
H 2.96931100 1.02544600 1.15457600
H 1.48361900 1.67213800 1.84773300
H 3.00280300 -1.23920800 0.05851200
H 1.61552700 -2.32268700 0.22386800
H 1.65466900 -1.28245100 -2.05938500
H 2.89244900 0.71515100 -1.52236000
H 1.36568800 1.19596700 -2.25976400
C -0.21058400 -0.82777300 -1.13185600
H -0.66739200 -0.40273800 -2.03054500
H -0.46713500 -1.90013500 -1.09290800
C -0.14092200 -0.54614300 1.30322900

H -0.39076600 -1.59767700 1.52474800

H -0.54892000 0.07520100 2.10600800

N 1.53562600 1.63628700 -0.23266600

N -0.73998600 -0.10218200 0.03430700

C -2.17653300 -0.06583900 0.06897200

O -2.75222500 0.98460000 -0.04368700

C -2.91726800 -1.37043700 0.24404900

H -2.67752100 -2.04934100 -0.57729200

H -2.60816700 -1.85938700 1.17022200

H -3.98847300 -1.18616400 0.26534400

H 0.53801500 1.81731900 -0.22478800

**E = -537.01297**

**TS3$_{Ac-H}$**

C 1.91326700 0.74515000 1.24083800

C 1.21753700 -0.62092800 1.21239000

C 1.67876400 -1.38311800 -0.03132700

C 1.21886600 -0.56854700 -1.24186700

C 1.91443400 0.79749100 -1.21110500

H 1.51743900 -1.16015600 2.11603200

H 2.99155200 0.55289900 1.38073100

H 1.56866500 1.32458200 2.10204300

H 2.76797700 -1.48955700 -0.03315500

H 1.24270300 -2.38667700 -0.05294200

H 1.52006500 -1.06863700 -2.16733400

H 2.99288200 0.61166300 -1.35808300

H 1.57049500 1.41328400 -2.04697500

C -0.31148700 -0.51455000 -1.24572000

H -0.66754900 0.10076800 -2.07864300

H -0.68839000 -1.53846400 -1.39820900

C -0.31286900 -0.56776800 1.21590200

H -0.68900800 -1.59771600 1.32290800

H -0.67090100 0.01009400 2.07441000

N 1.64525200 1.52002400 0.03085300

N -0.84905400 0.06001600 -0.00228900

C -2.28722900 0.00485400 -0.00399800

O -2.89592400 -1.03587500 -0.02661600

C -2.94684100 1.35141000 0.02489500

H -2.60368900 1.88864500 0.91240400

H -2.60422100 1.92597300 -0.83913900

H -4.03096000 1.26078900 0.02329200

H 2.18832500 2.37553700 0.04927100

**E = -537.008959**

**TS4$_{Ac\text{-}H}$**

C 1.92582700 1.01029700 -0.00383300

C 2.13110700 0.87328700 -1.52232700

C 1.38287000 -0.37005500 -2.01504100

C 1.97751700 -1.58302900 -1.29147100

C 1.77504600 -1.40948300 0.22366100

H 1.72317300 1.76988900 -2.00105500

H 0.86237500 1.21858700 0.17205100

H 2.48841400 1.86672700 0.37887400

H 0.31501200 -0.28212700 -1.79039100

H 1.48516200 -0.47852000 -3.10065900

H 1.45644500 -2.49560700 -1.59997100

H 0.69617600 -1.44910700 0.42291300

H 2.23230500 -2.24274700 0.76500000

C 3.44463800 -1.74087600 -1.71659900

H 3.93124100 -2.51228200 -1.11144400

H 3.45711400 -2.09089500 -2.75870200

C 3.60163800 0.76728500 -1.95170000

H 3.64572000 0.91431300 -3.04032200

H 4.18636800 1.57108100 -1.49348600

N 2.29638200 -0.16407500 0.78323300

N 4.22893200 -0.51013600 -1.61183800

H 3.30729200 -0.22163500 0.84045700

C 5.37360000 -0.50295600 -0.76679400

O 5.30970700 -0.38729600 0.43404000

C 6.68403800 -0.65280400 -1.48709700

H 6.66387600 -1.58735100 -2.05391600

H 6.77353500 0.15474500 -2.21840800

H 7.52452100 -0.64059700 -0.79646700

**E = -537.006301**

**TS5$_{Ac-H}$**

C -1.97725200 0.60665100 -1.26091000

C -1.11297200 -0.65933500 -1.22786400

C -1.48617100 -1.48165700 0.00674700

C -1.15838000 -0.61901900 1.22658400

C -2.02101600 0.64822800 1.18536300

H -1.32910400 -1.22781800 -2.13730000

H -3.02122500 0.27806400 -1.40958800

H -1.70178300 1.22689900 -2.11818300

H -2.55102200 -1.73302000 -0.00893800

H -0.92087400 -2.41903600 0.03279300

H -1.40957800 -1.15684300 2.14560500

H -3.07014300 0.32543200 1.30817400

H -1.77492300 1.29721200 2.03024800

C 0.35300000 -0.36927400 1.26122400

H 0.60710000 0.29957000 2.08871800

H 0.83953000 -1.34144000 1.45471800

C 0.39841600 -0.40765800 -1.21437000

H 0.89228000 -1.38441200 -1.35994700

H 0.68212400 0.23581400 -2.05225700

N -1.81670900 1.40271300 -0.04776100

N 0.84477700 0.24925300 0.02220400

C 2.26938500 0.40459300 0.04548200

O 2.76625900 1.50042500 0.03714200

C 3.14291900 -0.83715500 0.08101300

H 3.76818900 -0.79599200 0.97448300

H 2.57460000 -1.76556500 0.08130700

H 3.80659800 -0.81957300 -0.78507000

H -2.44563800 2.19667600 -0.07245700

**E = -537.005658**

**Ac-Me$_{Rp}$**

C 1.68272500 0.56304900 1.25716000

C 1.29067700 -0.91110300 1.08896400

C 1.78087100 -1.40655300 -0.27330700

C 1.09024200 -0.54426200 -1.33211300

C 1.53602700 0.90784300 -1.14399300

H 1.76737400 -1.47774800 1.89407200
H 2.78765700 0.62158400 1.31033000
H 1.28639100 0.95290100 2.19949700
H 2.86781700 -1.30618700 -0.35336100
H 1.53142600 -2.46351300 -0.41028700
H 1.38396800 -0.86489500 -2.33594800
H 2.63011000 0.96374200 -1.30997100
H 1.06431000 1.55356400 -1.89288100
C -0.43470800 -0.72669500 -1.21839800
H -0.94836300 -0.10651700 -1.94907800
H -0.67403600 -1.77097900 -1.45113000
C -0.22399300 -1.14875200 1.18405000
H -0.42235400 -2.22149700 1.07905100
H -0.62131400 -0.81425600 2.13921300
N 1.18136400 1.41176800 0.17712200
N -0.94044400 -0.44125900 0.12412300
C -1.59634700 0.71041600 0.45235100
O -1.80094600 1.03284900 1.61641400
C -2.10491800 1.57926000 -0.67775500
H -1.26011600 2.03393900 -1.20014300
H -2.69855200 1.01546200 -1.39861000
H -2.71688000 2.36410200 -0.24044800
C 1.61648800 2.78267100 0.35173600
H 1.18030200 3.41124500 -0.42833700
H 1.28028500 3.15531800 1.32135900
H 2.71575300 2.88188500 0.30234100

**E = -576.313299**

**TS1$_{Ac-Me}$**

C 1.89186900 0.75121900 1.23183200
C 1.21233300 -0.62117600 1.20917500
C 1.67940000 -1.38268200 -0.03136200
C 1.21356900 -0.56881600 -1.23865600
C 1.89211100 0.80374900 -1.20156600
H 1.51898900 -1.15163200 2.11560100
H 2.97935800 0.58231800 1.36621100
H 1.54743400 1.33097000 2.09449000

H 2.76915700 -1.48178400 -0.03301400
H 1.24880500 -2.38838100 -0.05309000
H 1.52194600 -1.05966100 -2.16656600
H 2.97970400 0.64167300 -1.34310100
H 1.54723200 1.42014100 -2.03826900
C -0.31701500 -0.52604200 -1.24467000
H -0.67655300 0.08426700 -2.07964600
H -0.68807100 -1.55256000 -1.39282200
C -0.31831400 -0.57833800 1.21432000
H -0.68912800 -1.61040700 1.31785100
H -0.67934200 -0.00460600 2.07422000
N 1.64089200 1.53931900 0.03172800
N -0.85479900 0.05086900 -0.00276000
C -2.29326100 -0.00225200 -0.00427900
O -2.90283800 -1.04234300 -0.02660000
C -2.95089100 1.34517400 0.02429800
H -2.60661000 1.88246100 0.91133800
H -2.60806300 1.91875200 -0.84032600
H -4.03510200 1.25591800 0.02329400
C 2.40304900 2.77118600 0.05823600
H 2.15566800 3.37980600 -0.81439800
H 2.15584600 3.34150300 0.95641600
H 3.49343700 2.58874000 0.05413400

**E = -576.282525**

**$TS2_{Ac\text{-}Me}$**

C -1.93746500 0.63114900 -1.25866000
C -1.10344500 -0.65292700 -1.22594700
C -1.49278300 -1.46613000 0.00828700
C -1.14906300 -0.60258600 1.22163900
C -1.98192600 0.68204800 1.16998000
H -1.33360600 -1.21429500 -2.13623200
H -2.99655300 0.33632900 -1.40422400
H -1.65251700 1.24555100 -2.11867800
H -2.56198600 -1.69764100 -0.00693400
H -0.94381500 -2.41278800 0.03816600
H -1.41406300 -1.12586400 2.14505000

H -3.04601000 0.39349300 1.28919800
H -1.72750700 1.33200000 2.01329700
C 0.36661800 -0.38070300 1.25727100
H 0.63272800 0.28269700 2.08524100
H 0.83695900 -1.36125800 1.44799000
C 0.41239400 -0.43014300 -1.21397900
H 0.88924800 -1.41686600 -1.34784700
H 0.70901700 0.19988500 -2.05745500
N -1.79086700 1.44188300 -0.05774800
N 0.86348100 0.23150500 0.01772600
C 2.28882000 0.38193500 0.04078900
O 2.78888700 1.47612300 0.02817400
C 3.15712700 -0.86291800 0.08176200
H 3.78086500 -0.82145700 0.97630600
H 2.58476400 -1.78884700 0.08405400
H 3.82255100 -0.85085100 -0.78307000
C -2.67605600 2.58775800 -0.09797200
H -2.50650800 3.21819800 0.77756800
H -2.47414200 3.18106500 -0.99235500
H -3.74158700 2.29292000 -0.11129200
**E = -576.279496**

**Boc-H$_{Rp}$**
C 1.76714700 0.32396900 1.54368100
C 1.45186600 -1.01079200 0.84500800
C 1.90175700 -0.92924800 -0.61565800
C 1.10416000 0.20187700 -1.26871500
C 1.42156300 1.52339400 -0.54575600
H 1.99726900 -1.80369300 1.36596400
H 2.85679200 0.44795700 1.56250400
H 1.42593500 0.30004300 2.58179900
H 2.97460400 -0.72078100 -0.67328500
H 1.71889000 -1.87861600 -1.13006300
H 1.39268500 0.31159300 -2.31870600
H 2.47987500 1.75636500 -0.71515900
H 0.83768600 2.34186400 -0.97512500
C -0.38710800 -0.14568200 -1.25267600

H -0.97472600 0.68864800 -1.62955700

H -0.56211500 -1.00788600 -1.91111500

C -0.03202600 -1.38047100 0.89947400

H -0.16188100 -2.40571300 0.52558600

H -0.40310900 -1.35014300 1.92287400

N 1.19251600 1.50808200 0.90084500

N -0.85584500 -0.46745300 0.10053600

C -2.15292900 -0.29762900 0.48276800

O -2.61380900 -0.71247000 1.53081600

H 0.19372400 1.51901900 1.07363700

O -2.85181300 0.40146300 -0.42924800

C -4.24369100 0.77228100 -0.18971100

C -5.11508300 -0.47439700 -0.07831200

H -4.89334200 -1.03343700 0.82857600

H -6.16491300 -0.17295800 -0.06106800

H -4.95849000 -1.11904800 -0.94637600

C -4.35194600 1.66662700 1.04101600

H -3.66038800 2.50816100 0.95445100

H -5.36752800 2.06426600 1.10222200

H -4.13411700 1.11494500 1.95350100

C -4.59906400 1.55663700 -1.44702600

H -5.63540200 1.89462400 -1.39126100

H -3.95090300 2.42958000 -1.54666100

H -4.48231900 0.92871100 -2.33249500

**E = -730.106695**

**TS1$_{\text{Boc-H}}$**

C 1.78341400 1.08825000 1.04944800

C 1.34318400 -0.37344400 1.25339600

C 1.88849500 -1.21838300 0.09889900

C 1.26599300 -0.67162200 -1.18869900

C 1.70663300 0.79297100 -1.36968200

H 1.75246500 -0.72419200 2.20602200

H 2.87631700 1.12493900 1.13447100

H 1.37676900 1.72202400 1.84227200

H 2.97970000 -1.14469400 0.05546700

H 1.63002200 -2.27338900 0.23586000

H 1.61827300 -1.24398600 -2.05257400
H 2.79174900 0.79996700 -1.52929800
H 1.24621400 1.22078500 -2.26445700
C -0.25260800 -0.84790300 -1.11620000
H -0.72891900 -0.43976900 -2.01298900
H -0.49001200 -1.92147500 -1.06132000
C -0.17573800 -0.54975100 1.31992000
H -0.40806300 -1.60311800 1.53989900
H -0.59813000 0.06605000 2.11997700
N 1.40658700 1.67685600 -0.23923200
N -0.80918600 -0.13561600 0.05165700
C -2.22051100 -0.32510800 0.11960000
O -2.75811700 -1.39456000 0.26376000
H 0.40233000 1.81706500 -0.22442600
O -2.84446100 0.83982000 0.00146000
C -4.30991000 0.92581700 0.03699000
C -4.90622100 0.14779400 -1.13056700
H -4.76601700 -0.92513100 -1.01178200
H -5.97677600 0.35710000 -1.18312500
H -4.44890400 0.47038700 -2.06879900
C -4.82851300 0.44346100 1.38695700
H -4.31719100 0.97102600 2.19548700
H -5.89530000 0.66720800 1.45467600
H -4.68811200 -0.62884900 1.51104000
C -4.55940700 2.41853200 -0.13073300
H -5.63276400 2.61589400 -0.12036800
H -4.09210600 2.97594600 0.68312000
H -4.14725700 2.76878800 -1.07884600

**E = -730.082886**

**TS2$_{Boc-H}$**

C 1.91664800 0.97222900 1.05839600
C 1.30669600 -0.42756500 1.26236300
C 1.76605900 -1.33676100 0.11900300
C 1.23074500 -0.72908700 -1.18064300
C 1.84173600 0.67332400 -1.36074500
H 1.66036700 -0.81791300 2.22183800

H 3.00483900 0.88159000 1.16213900
H 1.57360400 1.65403900 1.84131100
H 2.85891500 -1.39121300 0.09175000
H 1.38466400 -2.35388800 0.25650100
H 1.52757500 -1.34358700 -2.03640100
H 2.92228800 0.55206200 -1.50517700
H 1.44664900 1.14694800 -2.26357200
C -0.29954200 -0.72829500 -1.13120600
H -0.71197600 -0.26904900 -2.03467500
H -0.65778900 -1.76790500 -1.08051900
C -0.22374500 -0.42799100 1.30819700
H -0.57610800 -1.44646500 1.53253200
H -0.58375000 0.23910700 2.09726000
N 1.63250000 1.59269800 -0.23865700
N -0.78147900 0.04780500 0.02760800
C -2.20115600 0.13140600 0.06152700
O -2.80217500 1.16726600 -0.04710100
H 0.65317900 1.85561900 -0.24063300
O -2.76289600 -1.06700600 0.22646000
C -4.21799900 -1.23873600 0.29467300
C -4.77274700 -0.48793000 1.49961500
H -4.69113400 0.59009700 1.37149600
H -5.82648900 -0.74703600 1.62312100
H -4.23905700 -0.78538900 2.40530100
C -4.85579600 -0.79261100 -1.01590500
H -4.37264800 -1.29380000 -1.85791700
H -5.91067600 -1.07521600 -1.00968200
H -4.78409200 0.28535200 -1.14966100
C -4.36967700 -2.74203400 0.48153300
H -5.42781300 -3.00055300 0.55012000
H -3.93255900 -3.27636000 -0.36417100
H -3.87024600 -3.06357600 1.39748300

**E = -730.082856**

**Boc-Me$_{Rp}$**

C 1.24015000 0.30093900 1.51395300
C 1.45436700 -1.03460900 0.79340100

C 2.06329800 -0.76329200 -0.58427800
C 1.05521500 0.09277300 -1.35468300
C 0.86928200 1.42035600 -0.61412700
H 2.13776200 -1.63650900 1.39970600
H 2.23390200 0.75153200 1.70608400
H 0.76132900 0.13612800 2.48427000
H 3.01646400 -0.23425000 -0.48555700
H 2.25535100 -1.70295300 -1.11219100
H 1.43344600 0.31530300 -2.35694900
H 1.83525900 1.96318700 -0.62601500
H 0.13585600 2.04585200 -1.13371400
C -0.25345500 -0.69714500 -1.52130100
H -1.00473700 -0.10903300 -2.04069500
H -0.04425100 -1.58967100 -2.12249900
C 0.15896300 -1.83972700 0.61603600
H 0.40149000 -2.79972700 0.14612700
H -0.31999500 -2.03639000 1.57253200
N 0.40158300 1.22738600 0.75508300
N -0.80015300 -1.14467900 -0.23937600
C -1.84543800 -0.51412800 0.37223500
O -2.17020600 -0.69835000 1.53020200
O -2.51032300 0.28545600 -0.47993100
C -3.70352200 1.00494300 -0.05594500
C -4.79882400 0.02965200 0.36493400
H -4.54511700 -0.47386000 1.29549400
H -5.73222200 0.58066100 0.50162500
H -4.95398400 -0.71757700 -0.41702000
C -3.35887300 2.00035800 1.04626900
H -2.55923800 2.66284200 0.70924100
H -4.24003100 2.60721400 1.26825300
H -3.03974500 1.49017100 1.95327200
C -4.11520800 1.74409500 -1.32395900
H -5.00459900 2.34688800 -1.13079700
H -3.31024900 2.40337700 -1.65565900
H -4.33939300 1.03415100 -2.12271700
C 0.29745700 2.49506900 1.45003000

H -0.32585800 3.18347200 0.87601700
H -0.16456400 2.34428900 2.42850000
H 1.28465300 2.96883700 1.59815500
**E = -769.378688**

**TS1$_{Boc\text{-}Me}$**

C 1.85376400 1.07641300 1.04751300
C 1.33642300 -0.35161200 1.24441500
C 1.85082600 -1.22561900 0.10047200
C 1.26088400 -0.64568800 -1.18516400
C 1.77679600 0.78511600 -1.36576300
H 1.72779600 -0.71193500 2.20039900
H 2.95737200 1.04850300 1.15131100
H 1.47231700 1.73025000 1.83840300
H 2.94421600 -1.20484100 0.06411200
H 1.53702900 -2.26514400 0.23588000
H 1.59687600 -1.22549000 -2.04991400
H 2.87185900 0.72624800 -1.52955300
H 1.34048200 1.23478200 -2.26357300
C -0.26367400 -0.77346400 -1.13050700
H -0.71531100 -0.32672300 -2.02138500
H -0.51839900 -1.84521000 -1.12069600
C -0.18792400 -0.47512200 1.31456300
H -0.43555500 -1.51576800 1.57795300
H -0.58692700 0.17619100 2.09794000
N 1.47400700 1.65370900 -0.23542000
N -0.82738700 -0.09288000 0.04569800
C -2.23206700 -0.30767800 0.11603800
O -2.75383900 -1.38649900 0.25999500
O -2.88119900 0.84478800 0.00081400
C -4.34600700 0.90143800 0.04088800
C -4.93291800 0.11147600 -1.12377200
H -4.77343000 -0.95863000 -1.00402200
H -6.00711100 0.30232500 -1.17376700
H -4.48347700 0.44061300 -2.06358000
C -4.85196600 0.41103300 1.39302500
H -4.34919700 0.95053500 2.19907800

H -5.92304900 0.61266400 1.46338000

H -4.68840500 -0.65783900 1.51848400

C -4.62629600 2.38894200 -0.12737900

H -5.70333000 2.56534500 -0.11198000

H -4.16523200 2.95613900 0.68323100

H -4.22490600 2.74592700 -1.07760000

C 2.07986300 2.95747800 -0.41245500

H 1.73492800 3.40293200 -1.34813500

H 1.78964400 3.61618500 0.40901700

H 3.18395500 2.90538400 -0.44053400

**E = -769.349499**

**Boc-Cl$_{Rp}$**

C 1.07115200 0.34532700 1.59084700

C 1.34829500 -0.96345300 0.83819300

C 1.97531500 -0.63416400 -0.51811100

C 0.94431100 0.18544800 -1.29678600

C 0.67647000 1.49558500 -0.54371700

H 2.04431800 -1.54267700 1.45150600

H 2.02770800 0.85405900 1.78392800

H 0.58255400 0.14728700 2.54683000

H 2.90050200 -0.06498400 -0.38744000

H 2.22127600 -1.55269500 -1.05920100

H 1.33541700 0.45625300 -2.28165400

H 1.60868800 2.07885200 -0.49887200

H -0.08176500 2.09152600 -1.05693300

C -0.31791200 -0.66431100 -1.51307200

H -1.08265300 -0.10687200 -2.04591500

H -0.04586300 -1.53526500 -2.11993700

C 0.09122200 -1.81886400 0.62025000

H 0.38813800 -2.76184500 0.14812800

H -0.40225800 -2.04617100 1.56237600

N 0.17239500 1.20973100 0.80804600

N -0.87376200 -1.15373400 -0.25144400

C -1.97162800 -0.58643000 0.33615500

O -2.31945200 -0.80601500 1.47995300

O -2.64014800 0.19721300 -0.52440300

C -3.85821400 0.89073700 -0.12108100

C -4.94214100 -0.11101700 0.26378900

H -4.69381900 -0.62740000 1.18873300

H -5.88656600 0.42169000 0.39685200

H -5.07327400 -0.84586800 -0.53416800

C -3.55287000 1.88004100 0.99715400

H -2.74130100 2.54492700 0.69449600

H -4.44282700 2.48315200 1.19278000

H -3.26307600 1.36671300 1.91237600

C -4.25284600 1.63415400 -1.39171800

H -5.16160700 2.21271400 -1.21579400

H -3.45604400 2.31730700 -1.69336500

H -4.43829800 0.92857400 -2.20418100

Cl 0.00355200 2.73344000 1.66626800

**E = -1189.685215**

**TS1$_{Boc-Cl}$**

C 1.84845300 1.07307500 1.05698900

C 1.33954400 -0.36236800 1.24529900

C 1.86253700 -1.22977000 0.10078400

C 1.26248900 -0.65813200 -1.18339200

C 1.77144700 0.77654800 -1.37735000

H 1.73995700 -0.71101400 2.20138900

H 2.94709900 1.06014400 1.12379300

H 1.46449200 1.72762800 1.84211800

H 2.95515800 -1.20079300 0.06262300

H 1.55671300 -2.27081600 0.23719800

H 1.60485300 -1.22902500 -2.05103100

H 2.86388300 0.73950200 -1.50747700

H 1.33433300 1.22650500 -2.27118700

C -0.26042300 -0.79529100 -1.12625900

H -0.71668200 -0.35319500 -2.01658700

H -0.50747800 -1.86824700 -1.11301300

C -0.18287100 -0.49728000 1.31851300

H -0.42179300 -1.54030100 1.57831600

H -0.58552300 0.14876200 2.10383000

N 1.41016900 1.62371300 -0.23273800

N -0.81965400 -0.11277900 0.05006000

C -2.22809100 -0.31544700 0.11933000

O -2.75437600 -1.39162900 0.26316500

O -2.86541200 0.84112700 0.00304500

C -4.33091200 0.91137800 0.03954300

C -4.92024000 0.12732500 -1.12764200

H -4.76958700 -0.94420200 -1.00870700

H -5.99277700 0.32638200 -1.18009600

H -4.46605000 0.45430300 -2.06587800

C -4.84374400 0.42350400 1.38980900

H -4.33787800 0.95711500 2.19779800

H -5.91298000 0.63519600 1.45803100

H -4.69097400 -0.64714800 1.51397100

C -4.59619400 2.40149900 -0.12788500

H -5.67157100 2.58778500 -0.11577900

H -4.13251000 2.96345300 0.68481400

H -4.18837300 2.75579900 -1.07630200

Cl 2.18649800 3.19104900 -0.44776400

**E = -1189.656547**

**Boc-Bn$_{Rp}$**

C 1.64672400 -0.30005600 1.18047500

C 1.45381200 -1.67427200 0.52805000

C 1.93018900 -1.60278400 -0.92463900

C 1.04994400 -0.56954200 -1.63197900

C 1.26284300 0.79463100 -0.96675500

H 2.05346600 -2.39756000 1.08868500

H 2.73187500 -0.09936100 1.23003100

H 1.26600700 -0.31045900 2.20763000

H 2.98171900 -1.30181800 -0.96878400

H 1.84153500 -2.58118200 -1.40783300

H 1.34064800 -0.48028800 -2.68308100

H 2.31133000 1.09685900 -1.14123800

H 0.61963100 1.54960800 -1.43308200

C -0.41177200 -1.04516400 -1.59861400

H -1.07182700 -0.32262100 -2.07010500

H -0.48156900 -1.98547500 -2.15808800

C -0.00197700 -2.16192600 0.55320600

H -0.04167100 -3.17164300 0.12844000

H -0.38979600 -2.20156100 1.56855900

N 0.93962600 0.75296100 0.45556700

N -0.88237100 -1.30386700 -0.23686000

C -1.68755500 -0.43436100 0.43941700

O -1.90152200 -0.49093600 1.63622800

O -2.26355500 0.45756000 -0.38722700

C -3.29271500 1.37024300 0.09365900

C -4.47902900 0.59341500 0.65748900

H -4.22766500 0.11134700 1.60001700

H -5.30935100 1.28388600 0.82237900

H -4.80261200 -0.16545500 -0.05899700

C -2.70861000 2.34770800 1.10723300

H -1.87537700 2.89366600 0.66058500

H -3.47792800 3.06977300 1.39138700

H -2.36069800 1.82911300 1.99863100

C -3.70313200 2.10893300 -1.17498500

H -4.46254300 2.85693200 -0.93951000

H -2.84010400 2.61283800 -1.61520500

H -4.11362300 1.41057900 -1.90715800

C 1.02322400 2.05633200 1.10925500

H 0.39186900 2.74576200 0.54187600

H 0.58523400 1.95134400 2.10702800

C 2.41782500 2.64560400 1.22632700

C 3.21308000 2.38396000 2.34411100

C 2.94651000 3.43538400 0.20283000

C 4.50744800 2.88766500 2.43411100

H 2.80963400 1.78381100 3.15411200

C 4.24046700 3.94168000 0.28623200

H 2.33354800 3.66205200 -0.66435900

C 5.02541300 3.66605500 1.40245700

H 5.10980600 2.67718700 3.31026200

H 4.63374500 4.55533900 -0.51599000

H 6.03192400 4.06163500 1.47143300

**E = -1000.317616**

**TS1$_{Boc-Bn}$**

C 1.91136200 0.89099200 1.06738100

C 1.31188400 -0.50617600 1.26203900

C 1.78573300 -1.40856000 0.12254200

C 1.23328200 -0.80352100 -1.16825700

C 1.83508300 0.59360500 -1.35499700

H 1.67754700 -0.88667500 2.22045500

H 3.00548000 0.79743400 1.19580700

H 1.54979600 1.57072400 1.84733600

H 2.87899600 -1.44347700 0.09143300

H 1.41864800 -2.43030900 0.25962400

H 1.53896000 -1.40731400 -2.02792600

H 2.91978500 0.46173700 -1.52373300

H 1.42206900 1.06809800 -2.25241100

C -0.29635100 -0.84594900 -1.12063000

H -0.71814700 -0.37614600 -2.01433500

H -0.60735100 -1.90260500 -1.11305100

C -0.21714600 -0.54867200 1.32455800

H -0.51861000 -1.57461200 1.58934000

H -0.58508200 0.12372400 2.10541100

N 1.56530100 1.46861100 -0.22336400

N -0.83508400 -0.13800300 0.05275800

C -2.24745600 -0.30429600 0.11883500

O -2.80621400 -1.36438500 0.26251900

O -2.85736500 0.86916100 0.00005700

C -4.31994400 0.97331900 0.03447300

C -4.92734900 0.20377500 -1.13336500

H -4.80256000 -0.87096400 -1.01417900

H -5.99472100 0.42883300 -1.18705000

H -4.46418300 0.51933900 -2.07115500

C -4.84773000 0.49870000 1.38394400

H -4.32896100 1.01847000 2.19282600

H -5.91114300 0.73851900 1.45080800

H -4.72321700 -0.57551900 1.50812800

C -4.55100100 2.46921500 -0.13356900

H -5.62178000 2.68051400 -0.12399400

H -4.07652300 3.02045100 0.68031500

H -4.13310500 2.81391900 -1.08118000

C 2.05740200 2.82984100 -0.40558900

H 1.61962600 3.21661500 -1.33095000

H 1.66840100 3.43350900 0.42021300

C 3.56754700 2.97994300 -0.46559600

C 4.31130500 3.18164300 0.69918500

C 4.24805200 2.88231700 -1.68140000

C 5.69949300 3.27312700 0.65461900

H 3.79451100 3.27578400 1.64956200

C 5.63616200 2.97276900 -1.73308300

H 3.68181000 2.74201000 -2.59731300

C 6.36571700 3.16583600 -0.56316700

H 6.26056000 3.43323400 1.56807800

H 6.14780900 2.89830300 -2.68571400

H 7.44616800 3.23982200 -0.60108400

**E = -1000.288795**

**Boc-PEA$_{Sp\text{-}R}$**

C 1.73233200 -0.34556000 1.11127500

C 1.47884000 -1.67513400 0.39121500

C 1.90828700 -1.53899100 -1.07086000

C 1.04090200 -0.43823100 -1.68397600

C 1.32119000 0.87810200 -0.94766300

H 2.07258300 -2.44619700 0.89149500

H 2.82375000 -0.17305400 1.13336800

H 1.39069800 -0.41019200 2.15058000

H 2.96728200 -1.26983700 -1.13593600

H 1.76988100 -2.48562300 -1.60335700

H 1.30035200 -0.29326600 -2.73703200

H 2.37915100 1.14317000 -1.12590900

H 0.70074900 1.67221900 -1.37004400

C -0.43522600 -0.86446200 -1.63004300

H -1.08656800 -0.08107200 -2.01166300

H -0.56462500 -1.75527400 -2.25566100

C 0.01015000 -2.12135800 0.43812000

H -0.07072900 -3.09932000 -0.05121700

H -0.33835600 -2.22613400 1.46139300
N 1.02518800 0.76422900 0.47770000
N -0.87034900 -1.19488200 -0.27377800
C -1.67652200 -0.30521800 0.37161000
O -2.28521900 0.59368500 -0.17981500
O -1.75702600 -0.56882400 1.68883300
C -2.75561100 0.07863000 2.52936800
C -2.53372000 1.58634000 2.59254900
H -2.79156200 2.06572600 1.65080800
H -3.15739800 2.00209100 3.38770700
H -1.48867600 1.80196200 2.82816100
C -4.15682600 -0.27291100 2.04030000
H -4.27098500 -1.35749500 1.97551300
H -4.89161000 0.10798500 2.75326000
H -4.35466500 0.16833600 1.06451200
C -2.49396700 -0.54778400 3.89418000
H -3.20077400 -0.15364400 4.62665900
H -2.60936100 -1.63225800 3.84303600
H -1.47942300 -0.31778900 4.22720200
C 1.12636600 2.01220900 1.24822400
H 0.78014700 1.74235000 2.25278000
C 2.55523100 2.52665400 1.40824300
C 3.26578900 2.24171600 2.57640800
C 3.20644900 3.24241200 0.39893800
C 4.58528600 2.65459000 2.73871900
H 2.77454300 1.68875700 3.37162300
C 4.52734100 3.65407700 0.55291000
H 2.68163600 3.48945600 -0.51696200
C 5.22169700 3.36177600 1.72352600
H 5.11429500 2.42545500 3.65662800
H 5.01349700 4.20870800 -0.24146200
H 6.24837300 3.68668900 1.84442100
C 0.17113400 3.07600100 0.70919600
H 0.53186500 3.53884400 -0.21036800
H -0.80043100 2.62131300 0.50751500
H 0.05195000 3.86916000 1.45035500

**E = -1039.595083**

**Boc-PEA$_{Rp-R}$**

C 1.77445800 -0.15526000 1.03224200
C 1.51825400 -1.56065600 0.47048200
C 1.89987200 -1.58954600 -1.01052700
C 1.01971300 -0.55354900 -1.71172700
C 1.34921500 0.83110200 -1.14437000
H 2.13605000 -2.26409400 1.03658500
H 2.86556000 0.01943000 1.02488100
H 1.44285500 -0.10223600 2.07515000
H 2.95688000 -1.33793600 -1.14280200
H 1.73843900 -2.58837700 -1.42902100
H 1.23325700 -0.53862700 -2.78481800
H 2.41116000 1.04510300 -1.36491700
H 0.74311800 1.58513000 -1.65146900
C -0.46237400 -0.93498100 -1.54860800
H -1.10950600 -0.17343900 -1.97572100
H -0.63605700 -1.87298000 -2.08968900
C 0.05853100 -2.01151200 0.60722700
H -0.03304100 -3.03386800 0.22184200
H -0.26515700 -2.00650600 1.64517800
N 1.06493700 0.88070000 0.28446000
N -0.84519200 -1.15122500 -0.15233300
C -1.64636800 -0.30473600 0.55011200
O -1.84041300 -0.39113000 1.74988800
O -2.23607900 0.61665500 -0.23554700
C -3.43290800 1.31812100 0.22017700
C -4.54533400 0.30445900 0.46717900
H -4.29778400 -0.34947500 1.30331500
H -5.47164300 0.83340900 0.70202600
H -4.71033100 -0.30023000 -0.42757100
C -3.16821600 2.17537900 1.45633700
H -2.28820000 2.80327700 1.30720300
H -4.02922300 2.82985800 1.61402600
H -3.01791500 1.56338500 2.34193300
C -3.77009000 2.20609200 -0.97170100

H -4.68456900 2.76721200 -0.77067500

H -2.95998100 2.91479700 -1.15827200

H -3.91937900 1.60001400 -1.86741700

C 1.15678200 2.20307100 0.91953900

H 0.71206300 2.06401900 1.91177300

C 2.58796100 2.68629000 1.14315600

C 3.16623900 2.57492000 2.40912900

C 3.36990300 3.19851700 0.10303400

C 4.48420800 2.96083000 2.63732900

H 2.57267200 2.17946400 3.22791500

C 4.69040100 3.57999800 0.32330600

H 2.94895500 3.30968200 -0.88981500

C 5.25236900 3.46294200 1.59164100

H 4.90971300 2.86955300 3.63005200

H 5.27988100 3.97475900 -0.49625800

H 6.27884500 3.76470600 1.76349300

C 0.30674600 3.23399500 0.17761900

H 0.79014400 3.60408800 -0.72762800

H -0.64641200 2.78199200 -0.10327900

H 0.12182500 4.09205500 0.82659700

**E = -1039.59319**

**TS1$_{\text{Boc-PEA}}$**

C 1.92568000 0.87450000 1.03218900

C 1.32212400 -0.51718000 1.25187900

C 1.78631600 -1.43989600 0.12564400

C 1.23772000 -0.84422100 -1.17019100

C 1.84829300 0.54793800 -1.37574100

H 1.68988100 -0.88284900 2.21534900

H 3.02151900 0.77987600 1.14967400

H 1.57473100 1.56127000 1.81019200

H 2.87912800 -1.48478600 0.09332400

H 1.41012300 -2.45647700 0.27643500

H 1.53901900 -1.45832300 -2.02420200

H 2.93457900 0.40617300 -1.52506800

H 1.44550600 0.98988700 -2.29087600

C -0.29219300 -0.87605100 -1.12260300

H -0.71028200 -0.40865200 -2.01948600

H -0.60989800 -1.93063200 -1.10920400

C -0.20701700 -0.55104200 1.32108600

H -0.51233700 -1.57102200 1.60394700

H -0.56844800 0.13581500 2.09244300

N 1.56863100 1.44284800 -0.26093300

N -0.82785300 -0.15741600 0.04550500

C -2.24004500 -0.31873600 0.11574200

O -2.80269900 -1.37559300 0.26816100

O -2.84633100 0.85618100 -0.01078000

C -4.30829800 0.96583000 0.02404000

C -4.91989400 0.18821800 -1.13628800

H -4.79972200 -0.88590600 -1.00732700

H -5.98629600 0.41750700 -1.19140600

H -4.45592700 0.49306800 -2.07723400

C -4.83649600 0.50524100 1.37818600

H -4.31569100 1.03100300 2.18186900

H -5.89922600 0.74849000 1.44361300

H -4.71480500 -0.56814200 1.51211900

C -4.53402900 2.46102200 -0.15729700

H -5.60395500 2.67662400 -0.14843100

H -4.05641600 3.01783200 0.65095700

H -4.11595500 2.79545100 -1.10856500

C 2.03516200 2.82572800 -0.42391100

H 1.72399700 3.33534800 0.49435300

C 3.55417100 2.95942000 -0.50038900

C 4.28203900 3.24675600 0.65666600

C 4.26010900 2.75837500 -1.69033300

C 5.67155600 3.32956000 0.63383400

H 3.74939800 3.41270500 1.58837300

C 5.64956800 2.83707400 -1.71951400

H 3.72488600 2.54166700 -2.60818700

C 6.36051000 3.12273700 -0.55710400

H 6.21428600 3.55880300 1.54367100

H 6.17768700 2.67947400 -2.65292400

H 7.44190900 3.18805700 -0.58109000

C 1.31975700 3.52307500 -1.58010200

H 0.24146100 3.39678800 -1.46765700

H 1.55666600 4.58880300 -1.56884800

H 1.60953700 3.13330300 -2.55672300

**E = -1039.566481**

# 5. CRYSTALLOGRAPHIC DATA

CCDC 2455164-2455165 contain the supplementary crystallographic data for this paper. The data is available from the Cambridge Crystallographic Data Centre via www.ccdc.cam.ac.uk/structures.

**X-Ray Diffraction of NO-Bn** (CCDC 2455165)

*Sample specs*:

Sample description: prism, transparent, colorless with dimensions ca. 0.50 x 0.30 x 0.25 mm.

Mounting: on a glass fiber, with epoxy bicomponent glue

Comments: The sample shows strong pleochroism under polarized light. It changes from white-transparent to gray. The dimensions of the sample analyzed are generous, and it was cut from a larger prism-shaped crystal with a spade. The obtained fragments were taken, polished by mechanical ablation in a drop of perfluorinated oil and one promising sample selected for diffraction.

*Instrumental specs*:

Device: Rigaku XtaLAB Synergy-S 4-circle diffractometer

Source: microfocus sealed tube

Detector: Hybrid Photon Counting (HPC)

Experiment temperature: 296(2) K

Cryostat: not used

Wavelength: Cu $K_\alpha$ (1.54184 Å).

Data collection extent: full sphere within sin $\vartheta/\lambda$ = 0.60 $Å^{-1}$

Data collections specs: Detector-to-sample distance: variable, several ω-scan run.

Measured reflections: 61942, 868 independents

Maximum resolution ($\vartheta$): 80.360 °

Completeness: 99.6 % (at full sphere resolution)

*Data reduction programs*:

Integration: CrysalisPro

Reduction: CrystalsPro

Structure solution and refinement: Shelxs2018, Shelxl 2018

*Unit cell, lattice and crystal system*:

Bravais lattice: Monoclinic, Primitive

Space group: P $2_1$/n, n ° 14

Point group: 2/m

Laue group: 2/m, number 2

Unit cell (Å, deg, $Å^3$): a = 6.99605(5), b = 8.15120(10), c = 23.0869(2), α = 90.0, β = 91.9090(10), γ = 90.0, V = 1315.84(2) as estimated from 36832 intense reflections among 3.8260 e 79.4900 deg of $\vartheta$ (final integration result).

Formula units in cell (Z): 4

Formula units in the asymmetric unit (Z'): 1

Number of electrons in cell ($F_{000}$): 528

<u>Computed density</u>: 1.238 g/cm$^3$

<u>Linear absorption coefficient</u> (μ): 0.637 mm$^{-1}$

*Main statistical results*:

Final stats for the spherical atom model (Shelxl):

Scale factor: 8.70 (3)

BASF parameter: //

Secondary extinction coefficient: none

$<\Delta/\sigma> = 0.000$

R1(F) = 0.0470 for 2667 > 4$\sigma(F_o)$, 0.0490 for all the 2868 independent data

wR($F^2$) = 0.1346 for all the measured data

Goodness-of-fit: 1.049

Flack's parameter: //

$\Delta\rho_{MAX/MIN}$ = +0.224 e/Å$^3$ at ~ 0.75 Å from the O1B oxygen, –0.184 e/Å$^3$ at ~ 0.62 Å from the N3B nitrogen.

The least squares statistical analysis indicates that the overall quality of the collected data is high, as the R1 agreement parameter is below 5% for all the collected independent data. Small residual electron densities are observed around the N-nitroso group. Besides, the same group shows a structural disorder, where at least two possible conformations of the N-N-O bond are possible. Further details can be found in the next sections.

In general, refined geometry is reliable, both in terms of chemical connectivity and absolute stereochemistry.

*Molecular schemes*

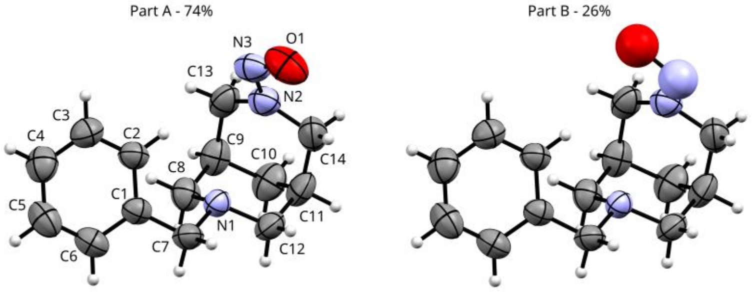


**Figure S5**. The two conformers of **NO-Bn** at 296 K, with population distribution of 74% (left) and 26% (right). Labels for non-H atoms are depicted only on the first conformer. Thermal ellipsoids of non-H atoms were drawn at the 50% probability level. The color code employed for atoms is grey: C; white: H; blue: N; red: O.

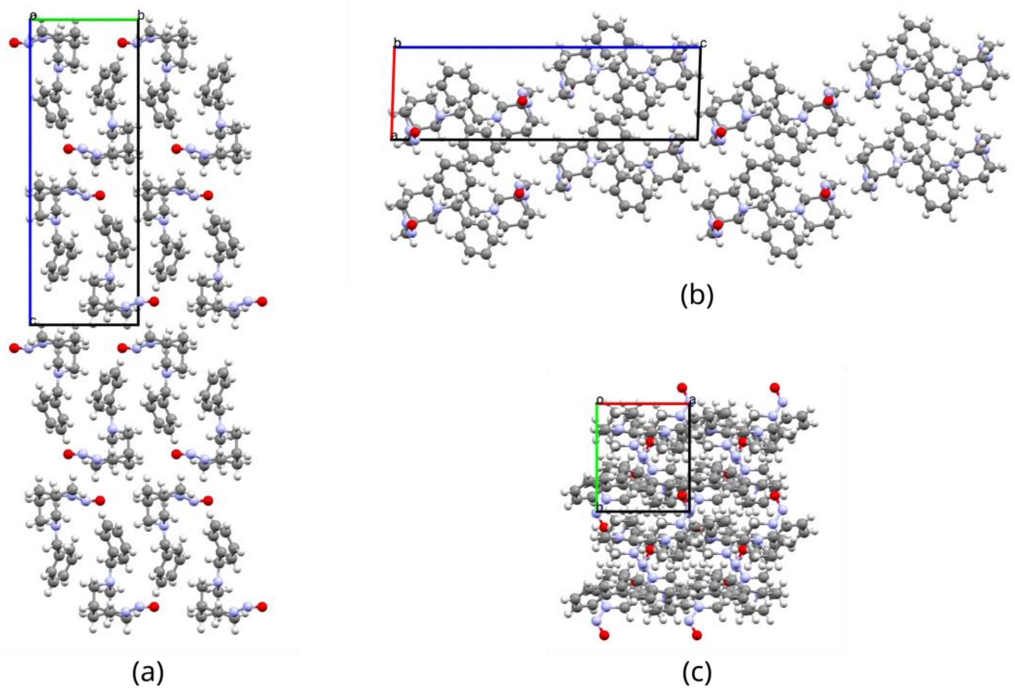


**Figure S6.** Crystal packing of **NO-Bn** at 296 K, as seen (a) along the *a* cell axis; (b) the *b* cell axis; (c) the *c* cell axis. Color code as in Figure 1.

*Discussion and conclusions*:

(1) The compound crystallizes in the monoclinic, achiral, centrosymmetric space group $P2_{1/n}$ (N. 14). The bispidine scaffold assumes a chair-chair conformation, with the benzyl group rotated by almost 70° with respect to a ring-ring planar conformation, assuming the plane of the bispidine as passing through atoms C8-C12-C13-C14 (see Figure S5). The terminal N-nitroso group is structurally disordered, with two possible orientations observed in the crystal. The first orientation (Figure S5, left) is the more populated, with a refined occupation factor of 0.74 (74%). The N-N bond length is 1.332 Å, and the C-N-N angles fall within the range 113-125°, values that agree with the Cambridge Structural Database (CSD) distribution. The second conformer (Figure S5, right) has a residual occupation factor of 0.26 (26%), a value that is large enough, even at room temperature, to consider the refined model accurate. The N-N bond length in this conformer is 1.329 Å, consistent with the major conformer. In contrast, the two C-N-N angles are more unusual, measuring 97° and 142°, respectively. This deviation from ideality is not considered problematic. A search of analogous structures in the CSD revealed that, in many cases, residual electron density peaks are observed in the same position as this second refined conformer. Moreover, the statistical comparison between model 1 (m1), which does not include any disorder, and model 2 (m2), where the disordered NO atoms are treated isotropically, clearly indicates that accounting for disorder is necessary.

Model m1 yields an R1 value of about 7% for strong reflections, with residual electron densities of +0.587/-0.372 e/Å$^3$ near the NO group. Introducing isotropic disorder in the model m2 improves the fit, reducing the R1 value of about 2% (R1=4.70%) and halving the residual electron density to +0.224/-0.184 e/Å$^3$.

A third model (m3), in which anisotropic displacement parameters were applied to the NO atoms in both conformers, was also tested. Although this model provided an even better agreement factor (R1=4.26%), the positions of the N and O atoms were found to be split during the refinement, resulting in elongated thermal ellipsoid.

Despite the improved agreement statistics, model m2 was retained as the final model due to its greater refinement stability and chemical soundness. The overall quality of the model is not affected by the absence of anisotropic displacement parameters for the N and O atoms.

Figure S7 illustrates the main packing motif of NO-Bn. No strong intermolecular hydrogen bonds are formed between molecules. The main packing motif is defined by weak dispersion/repulsion interactions, especially C-H···O contacts. Table S1 shows the shortest intermolecular contact between the molecules.

**Table S1**: C-H···O short contacts

| D–H···A | $d_{D–H}$, Å | $d_{H···A}$, Å | $d_{D···A}$, Å | $a_{DHA}$, ° | Symmetry operation |
|---|---|---|---|---|---|
| C3–H3···O1B | 0.93 | 2.45 | 3.084(10) | 125 | 5/2-x,1/2+y,1/2-z |
| C13–H13A···O1A | 0.97 | 2.51 | 3.319(3) | 141 | 2-x,-y,-z |
| C14–H14A···O1B | 0.97 | 2.41 | 3.201(10) | 139 | 2-x,-y,-z |

Inversion-related molecules are arranged with their N-nitroso groups facing each other, suggesting a possible directional preference in the packing. This configuration may support weak dipole–dipole interactions between the NNO groups, which could contribute to the stabilization of the crystal structure alongside dispersion forces. However, further computational analysis would be required to confirm the presence and significance of such interactions.

**X-Ray Diffraction of 7** (CCDC 2455164)

*Sample specs*:

Sample description: prism, transparent, colorless with dimensions ca. 0.40 x 0.15 x 0.10 mm.

Mounting: on a glass fiber, with perfluorinated oil

Comments: The sample does not show strong pleochroism under polarized light. It slightly changes from transparent to light gray, but the phenomenon is quite quenched. It was cut from a larger agglomerate using a spade and polished by mechanical ablation in a drop of perfluorinated oil.

*Instrumental specs*:

Device: Rigaku XtaLAB Synergy-S 4-circle diffractometer

Source: microfocus sealed tube

Detector: Hybrid Photon Counting (HPC)

Experiment temperature: 297.82(1) K

Cryostat: not used

Wavelength: Cu $K_a$ (1.54184 Å).

Data collection extent: full sphere within sin $\vartheta/\lambda = 0.64$ $Å^{-1}$

Data collections specs: Detector-to-sample distance: variable, several ω-scan run.

Measured reflections: 61572, 3322 independents

Maximum resolution (J): 80.210 °

Completeness: 99.5 % (at full sphere resolution)

*Data reduction programs*:

Integration: CrysalisPro

Reduction: CrystalsPro

Structure solution and refinement: Shelxs2018, Shelxl 2018

*Unit cell, lattice and crystal system*:

Bravais lattice: Monoclinic, Primitive

Space group: P $2_1$, n º 4

Point group: 2

Laue group: 2/m, number 2

Unit cell (Å, deg, $Å^3$): a = 7.13676(4), b = 10.09130(10), c = 10.67620(10), α = 90.0, β = 92.8890(10), γ = 90.0, V = 767.914(7) as estimated from 39696 intense reflections among 4.1430 e 79.6210 deg of ϑ (final integration result).

Formula units in cell (Z): 2

Formula units in the asymmetric unit (Z'): 1

Number of electrons in cell ($F_{000}$): 336

Computed density: 1.377 g/$cm^3$

Linear absorption coefficient (m): 0.864 $mm^{-1}$

*Main statistical results*:

Final stats for the spherical atom model (Shelxl):

Scale factor: 14.43 (3)

BASF parameter: //

Secondary extinction coefficient: none

$\langle\Delta/\sigma\rangle$ = 0.000

R1(F) = 0.0319 for 3221 $F_o > 4s(F_o)$, 0.0327 for all the 3322 independent data

wR($F^2$) = 0.0832 for all the measured data

Goodness-of-fit: 1.054

Flack's parameter: 0.00(22) by classical fit, 0.00(7) from 1451 selected quotients (Parsons's method)

$Dr_{MAX/MIN}$ = +0.15 e/$Å^3$ at ~ 0.99 Å from the O16 oxygen, –0.23 e/$Å^3$ at ~ 0.81 Å from the O16 oxygen.

The least squares statistical analysis indicates that the overall quality of the collected data is high, as the R1 agreement parameter is far below 5% for all the collected independent data. Small residual electron densities are observed around the nitrosamine oxygen atom. Besides, the same atom has a large thermal ellipsoid, indicating the possible presence of conformational disorder. In general, the refined geometry is reliable, both in terms of chemical connectivity and absolute stereochemistry, as also indicated by the refined Flack parameter.

*Molecular schemes*

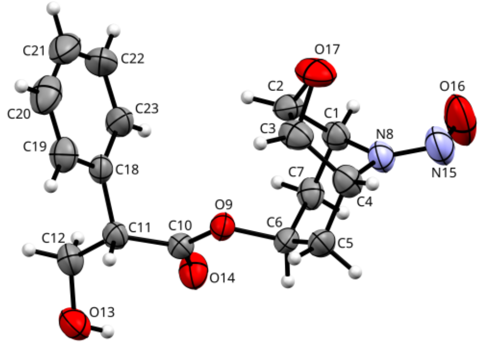


**Figure S8**. Asymmetric unit of **7** at 298 K, with the non-H atom-numbering scheme. Thermal ellipsoids of non-H atoms were drawn at the 50% probability level. The color code employed for atoms is grey: C; white: H; blue: N; red: O.

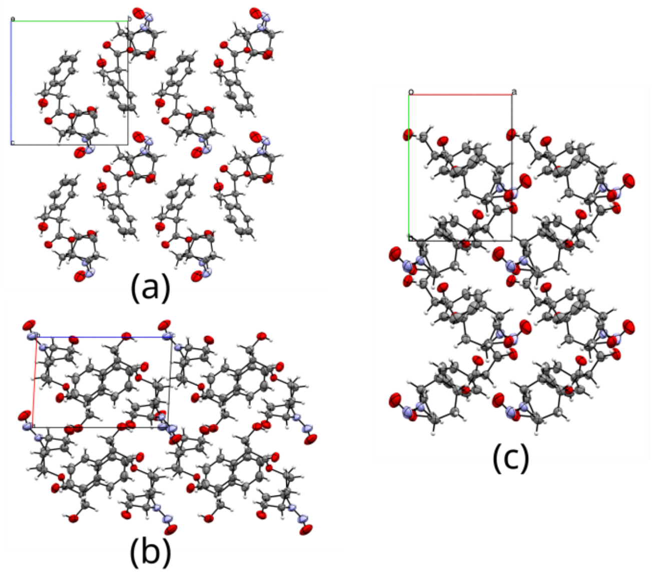


**Figure S9.** Crystal packing of **7** at 298 K, as seen (a) along the *a* cell axis; (b) the *b* cell axis; (c) the *c* cell axis. Color code as in Figure 1.

*Discussion and conclusions*:

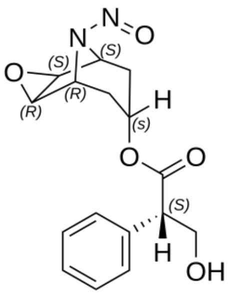


**Figure S10**. Molecular structure of **7**, with the CIP descriptors in parentheses.

The compound crystallizes in the monoclinic, achiral, non-centrosymmetric space group $P2_1$ (N. 4). The X-ray data confirm that the C11 stereocenter has an (S) configuration according to the CIP rules.

The thermal ellipsoid of the nitrosamine oxygen O16 is quite large. This is partially due to its terminal position on the functional group. However, the presence of small residual electron density around the

same oxygen is indicative of possible structural disorder, due to the lack of strong intermolecular interactions. We attempted to model the disorder into SHELX, which led to two possible positions for the O atom, with the site occupancy factor of the most probable one being 0.61. The two disordered positions are only 0.44 Å apart, a value consistent with movement of terminal groups when strong intermolecular interactions are absent. The disordered model results in a slight improvement of the R1 agreement factor, decreasing it by 0.016. Given the minimal improvement, we opted for the simpler model, as it does not significantly impact on the overall quality of the results. In any case, the disordered model at room temperature is not compatible with the presence of different conformations of the nitrosamine group due to its rotation. Only a single structural conformation of the title functional group is observed.

Figure S9 illustrates the main packing motif of **7**. No strong intermolecular hydrogen bonds are formed between molecules. Conversely, a cyclic intramolecular interaction is established between the hydroxyl group (O13-H13) and O14, as can be seen from Figure 1.

The main packing motif is defined by weak dispersion/repulsion interactions, especially C-H···O contacts. Table S2 shows the shortest intermolecular contact between the molecules.

**Table S2**: Intramolecular and C-H···O short contacts

| D–H···A | $d_{D–H}$, Å | $d_{H···A}$, Å | $d_{D···A}$, Å | $a_{DHA}$, ° | Symmetry operation |
|---|---|---|---|---|---|
| O13–H13···O14 | 0.82 | 2.30 | 2.845(2) | 124 | Intramolecular |
| C5–H5B···O14 | 0.97 | 2.48 | 3.276(3) | 139 | 1-x,1/2+y,2-z |
| C12–H12A···O17 | 0.97 | 2.58 | 3.468(3) | 152 | 1-x,-1/2+y,1-z |

## 6. REFERENCES

1) D. Stead, P. O'Brien, A. J. Sanderson, *Org. Lett*. **2005**, *7*, 4459.

2) A. Roy, D. Saha, P. S. Mandal, A. Mukherjee, P. Talukdar, *Chem. Eur. J*. **2017**, *23*, 1241.

3) P.-W. Phuan, J. C. Ianni, M. C. Kozlowski, *J. Am. Chem. Soc*. **2004**, *126*, 15473.

4) A. Misra, K.S. A. Kumar, M. Jain, K. Bajaj, S. Shandilya, S. Srivastava, P. Shukla, M. K. Barthwal, M. Dikshit, D. K. Dikshit, *Eur. J. Med. Chem*. **2016**, *110*, 1.

5) Gaussian 09, Revision C1, Frisch, M. J.; Trucks, G. W.; Schlegel, H. B.; Scuseria, G. E.; Robb, M. A.; Cheeseman, J. R.; Scalmani, G.; Barone, V.; Mennucci, B.; Petersson, G. A.; Nakatsuji, H.; Caricato, M.; Li, X.; Hratchian, H. P.; Izmaylov, A. F.; Bloino, J.; Zheng, G.; Sonnenberg, J. L.; Hada, M.; Ehara, M.; Toyota, K.; Fukuda, R.; Hasegawa, J.; Ishida, M.; Nakajima, T.; Honda, Y.; Kitao, O.; Nakai, H.; Vreven, T.; Montgomery, Jr., J. A.; Peralta, J. E.; Ogliaro, F.; Bearpark, M.; Heyd, J. J.; Brothers, E.; Kudin, K. N.; Staroverov, V. N.; Kobayashi, R.; Normand, J.; Raghavachari, K.; Rendell, A.; Burant, J. C.; Iyengar, S. S.; Tomasi, J.; Cossi, M.; Rega, N.; Millam, J. M.; Klene, M.; Knox, J. E.; Cross, J. B.; Bakken, V.; Adamo, C.; Jaramillo, J.; Gomperts, R.; Stratmann, R. E.; Yazyev, O.; Austin, A. J.; Cammi, R.; Pomelli, C.; Ochterski, J. W.; Martin, R. L.; Morokuma, K.; Zakrzewski, V. G.; Voth, G. A.; Salvador, P.; Dannenberg, J. J.; Dapprich, S.; Daniels, A. D.; Farkas, Ö.; Foresman, J. B.; Ortiz, J. V.; Cioslowski, J.; Fox, D. J. Gaussian, Inc., Wallingford CT, 2009.
